# Mesoscale simulations of membrane-tethered reactions to parameterize cell-scale models of signaling

**DOI:** 10.1016/j.bpj.2026.04.015

**Published:** 2026-04-25

**Authors:** Kelvin J. Peterson, Boris M. Slepchenko, Leslie M. Loew

**Affiliations:** 1 R. D. Berlin Center for Cell Analysis and Modeling, University of Connecticut School of Medicine, Farmington, CT, USA

## Abstract

Biochemical interactions at membranes are starting points for cell signaling. But reaction kinetics are difficult to measure on two-dimensional (2D) membranes and are usually measured in volumetric assays. Membrane tethering produces confinement and steric effects that will significantly impact binding rates; these cannot be determined by volumetric measurements. Additionally, because of the properties of 2D diffusion, bimolecular reactions may not conform to simple mass action kinetics. Here, we show how simulations using the SpringSaLaD software can be used to estimate a 2D rate constant based on a known 3D rate constant and coarse-grained molecular structures for the reactants; this approach accounts for confinement of the reaction to the near-membrane space as well as the steric environment and flexibility of the membrane-anchored binding sites. The approach is validated using theoretical solutions for dimerization in an idealized system containing a binding site at the end of a single stiff membrane anchor. With this ideal system, we also assess whether simple mass action rate constants can correctly describe the reaction rate, considering the diffusivity of the membrane anchors, the initial membrane densities of the reactants, and the desired level of completion of the reaction. We explore how factors such as molecular reach, steric effects, disordered domains, and diffusion affect the kinetics. We then apply our approach to epidermal growth factor receptor (EGFR)-mediated activation of the membrane-bound small GTPase Ras. The analysis reveals how binding of Ras to the allosteric site of SOS, a guanine nucleotide exchange factor that is recruited to EGFR, significantly accelerates Ras binding to the SOS catalytic site. A biochemical network model parametrized with the derived 2D rate constants demonstrates how recruitment of SOS via EGFR can significantly enhance Ras activation. Thus, we offer a novel method to more rigorously parameterize receptor-mediated steps in cell signaling.

## Significance

In cell signaling, the activation of a surface receptor leads to a cascade of intracellular biochemical events. Many protein interactions occur near the inner plasma membrane surface. However, accurate rate parameters for these steps in models of signaling are rarely available because membrane-tethered reaction kinetics are difficult to experimentally measure. Here, we use a coarse-grained molecular simulator to model the kinetics of reactions between binding sites that are tethered to a membrane. We can fit these simulation outputs with two-dimensional rate laws to obtain rate constants that can be used to build complex models of cell signaling. The derived rate constants can also be analyzed to understand the key biophysical features controlling the kinetics of bimolecular membrane reactions.

## Introduction

The cell membrane responds to and integrates electrical, mechanical, and chemical signals from the extracellular environment. For chemical signals, the initial step is binding of a ligand to an external binding site on a membrane receptor protein. This triggers a chain of events that typically involves a change of state of the cytoplasmic receptor domain and subsequent recruitment of adapter proteins, enzymes, and/or cytoskeletal regulators to further evoke a cell biological response. Mathematical modeling of signaling pathways is a powerful tool to systematically organize the experimental knowledge we have about these complex systems and then develop predictions, through simulations, to inspire new experiments.^1^^,^^2^^,^^3^^,^^4^^,^^5^

A challenge in developing cell signaling models is the acquisition of the appropriate experimentally grounded input parameters. Often, kinetic data are available from *in vitro* biochemistry, and this has served the mature field of metabolic modeling very well. However, rate parameters are less available and more difficult to measure for signaling pathways and networks. Among the key challenges is that many of the essential steps are associated with the plasma membrane, where multiple molecules are recruited before a messenger ultimately diffuses to an intracellular target (e.g., the nucleus). It is experimentally difficult to measure reaction rates on membranes because complex reconstituted bilayer methods must be used,^6^^,^^7^ so available data are commonly derived from volumetric measurements. While such quantitative data are useful, it can be challenging to translate rate parameters derived from 3D solution to the very different biophysical environment of a 2D membrane.

Indeed, the biophysics of membrane-associated reactions has a long scientific history. An early focus of investigation, initiated with a classic paper by Adam and Delbruck,^8^ was the difference between 2D and 3D diffusion-limited reactions; they argued that the 2-step process of absorbing a cytosolic molecule to the membrane and subsequent 2D search for an enzyme or binding partner might offer a kinetic advantage over a fully 3D search. This argument emerges from the fundamental difference between 2D and 3D diffusion: a diffusing molecule in 2D is guaranteed to eventually find its target, while no such guarantee can be made in 3D.^9^

An additional consequence of the difference between 2D and 3D diffusion is that bimolecular reactions on a surface cannot be fundamentally described by mass action kinetics.^10^^,^^11^^,^^12^^,^^13^^,^^14^^,^^15^^,^^16^ With regard to cell signaling, theoretical analyses of reactions at membranes have been extended and elaborated to consider both diffusion-limited and reaction-limited bimolecular kinetics.^10^^,^^12^^,^^13^^,^^17^ Intuitively, for reaction-limited cases (where diffusion is fast compared with the intrinsic reaction rate upon encounter) the Adam and Delbruck idea is not pertinent, and the reaction can be well described by simple mass action kinetics. In particular, Yogurtcu and Johnson^12^ developed the theory to determine where the transition from reaction-limited to diffusion-limited 2D kinetics occurs—i.e., when mass action in 2D is an adequate description or when a more complex time-dependent parameterization of the rate is required.

These pioneering studies treated membrane reactions as strictly 2D surface events. However, most biological membrane-associated reactions actually occur in the immediately adjacent cytosol, with interacting sites tethered to the membrane through lipid or protein anchors. A well-known feature of anchoring bimolecular reactions to a membrane is the effect of locally increased effective concentration: compared with the same reaction by the same number of molecules within the cell volume, anchoring the reaction to the membrane generally increases concentration by confining the reaction volume to a thin layer above the membrane.^18^^,^^19^ The thickness of that layer is often parametrized as *h,* sometimes called the “confinement length.”^20^^,^^21^ Essentially, *h* is related to the distance the binding sites can sample above the membrane surface.^21^^,^^22^^,^^23^^,^^24^ The smaller *h*, the greater is the effective concentrations of binding sites and the greater is the effective 2D affinity of the binding reaction. Estimates of *h*, and generally the interactions between membrane-bound reactants, can be derived by analyzing detailed molecular dynamics simulations of the flexibility and motions of binding domains tethered to the membrane.^21^^,^^25^^,^^26^

Recently, the concept of *molecular reach* was introduced as a more general framework for assessing how molecular structure influences the steady-state phosphorylated fraction of a membrane-bound substrate interacting with a tethered kinase.^27^ The *reach* is defined as the distance of the kinase site from the membrane anchor and is directly related to *h* when the anchor diffuses freely in the membrane. However, when lateral diffusion is restricted (e.g., within large signaling clusters such as the immune synapse), binding sites with longer *reach* may have an advantage in being able to find more binding partners; for such diffusion-limited scenarios, this increase in reach can outweigh the decrease in local concentration associated with increased *h*.^27^

Thus, it is clear from these foundational studies that the membrane diffusion and the structural features of interacting membrane-bound molecules must be considered when converting a measured 3D on rate to a 2D on rate suitable for cell-scale continuum models based on ordinary or partial differential equations (ODEs or PDEs). While detailed molecular dynamics calculations can be utilized to determine *h* or even rate constants,^21^^,^^25^^,^^26^ such intensive calculations are not always feasible to parameterize a large signaling model. In this work, we show how this can be done using simulations from SpringSaLaD^28^ to derive 2D rate constants. Importantly, we are treating tethered binding sites. This is in fact the more general situation in cell signaling where a more-or-less flexible protein domain containing the binding site extends into the cytosol a significant distance from the actual membrane—typically much longer than the actual thickness of the lipid bilayer. Because our method accounts for the structure of the protein (albeit very coarsely), the distance of the binding sites from the membrane as well as other biophysical features are accounted for in the estimation of the 2D on-rate constant.

SpringSaLaD uses a series of variously sized spherical sites linked together with stiff springs to coarsely model the key structural features of macromolecules such as flexibility, excluded volume, and binding site localization. Each sphere within the molecule can be assigned its own diffusion coefficient, and Brownian diffusion is simulated via a Langevin dynamics algorithm. The molecule can be tethered to a surface, representing a membrane, via a specialized anchoring sphere that has a lateral diffusion coefficient; the rest of the molecule, including the spheres designated as binding sites, is free to explore the volume above the membrane within its reach. Naturally therefore (and particularly advantageous for the purpose of this work), volumetric rate constants are used for bimolecular rate expressions even for membrane-bound molecules. In previous work, this feature was used to show how multivalent clustering is enhanced when one of the interacting molecules is tethered to a membrane.^29^

To determine 2D rate constants, we fit the stochastic kinetics simulated with SpringSaLaD to a deterministic mass action rate law based on the corresponding surface densities of the binding partners. Using idealized structures, we validate this procedure against theoretical calculations. We then explore how molecular structural features (e.g., tether length and stiffness, steric access), surface density, and lateral diffusion affect binding kinetics. Additionally, we develop a theory for membrane-tethered reactions, similar to that of Yogurtcu and Johnson for purely 2D reactions,^12^ to assess the appropriateness of a mass action rate constant for varying tether lengths, anchor diffusion coefficients, reaction rates, initial surface densities, and the desired level of completion of the reaction. Then, as a biologically relevant example, we apply this approach to recruitment of SOS to the epidermal growth factor receptor (EGFR). SOS is the G-protein exchange factor (GEF) for Ras,^30^ and a better understanding of SOS activation of Ras emerges from this analysis. Thus, we offer a procedure for parameterizing membrane kinetics that serves to bridge molecular to cell-scale simulations of cell signaling.

## Material and methods

**Figure 1:**
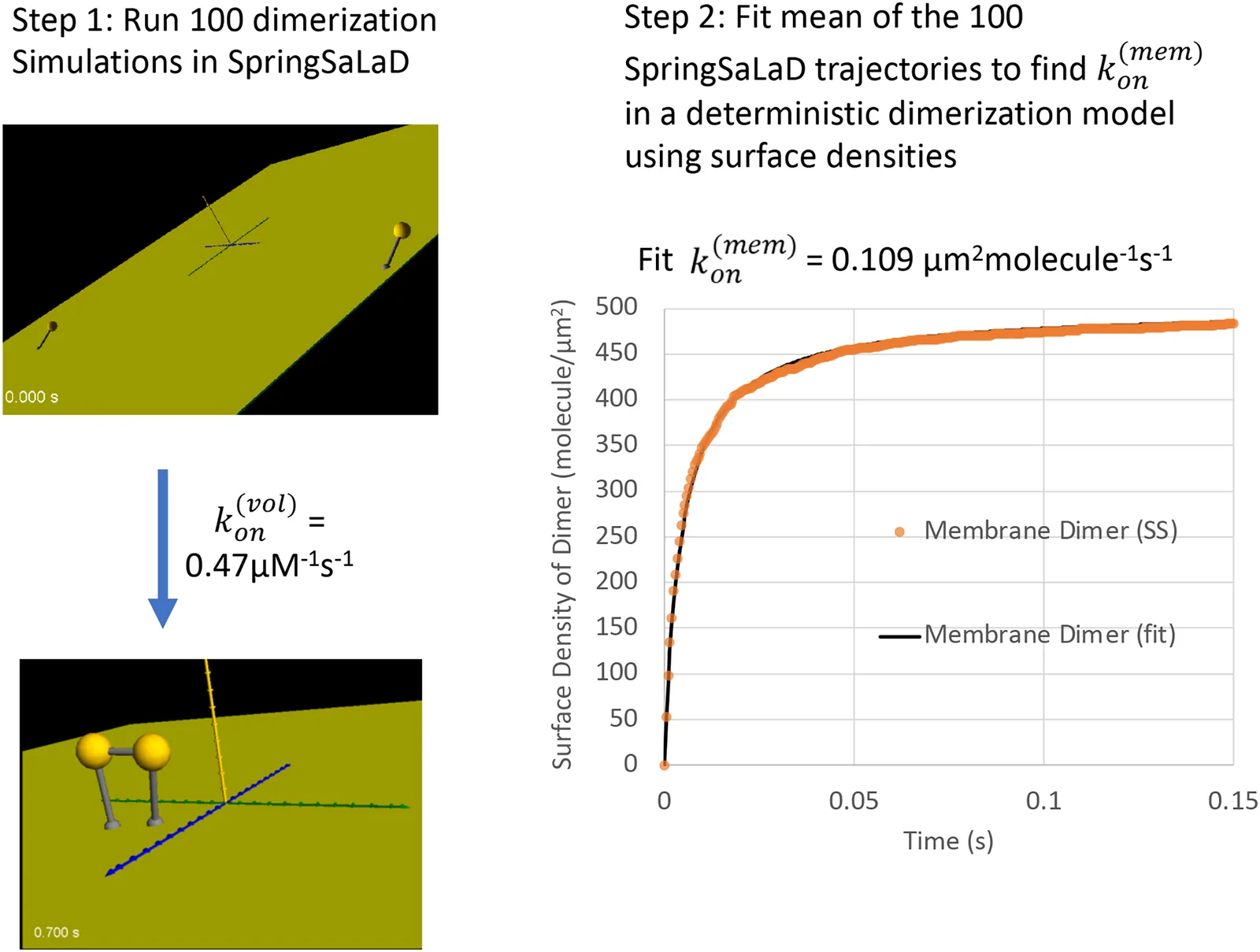
Workflow for finding membrane bimolecular binding rate constants ($k_{on}^{\left(mem\right)}$) in terms of surface densities. Step 1 is to run 100 SpringSaLaD trajectories with the coarse-grained model of the binding partners. Illustrated are a pair of simple monomers (*top*) consisting of 2-nm-diameter spheres (yellow) tethered to a 1-nm membrane anchor sphere (gray) with a 5-nm link; at the bottom, the product dimer is depicted. Step 2 consists of fitting the average of 100 outputs from stochastic SpringSaLaD simulations to a deterministic (ODE) nonspatial model of surface-bound dimerization.

**Figure 2:**
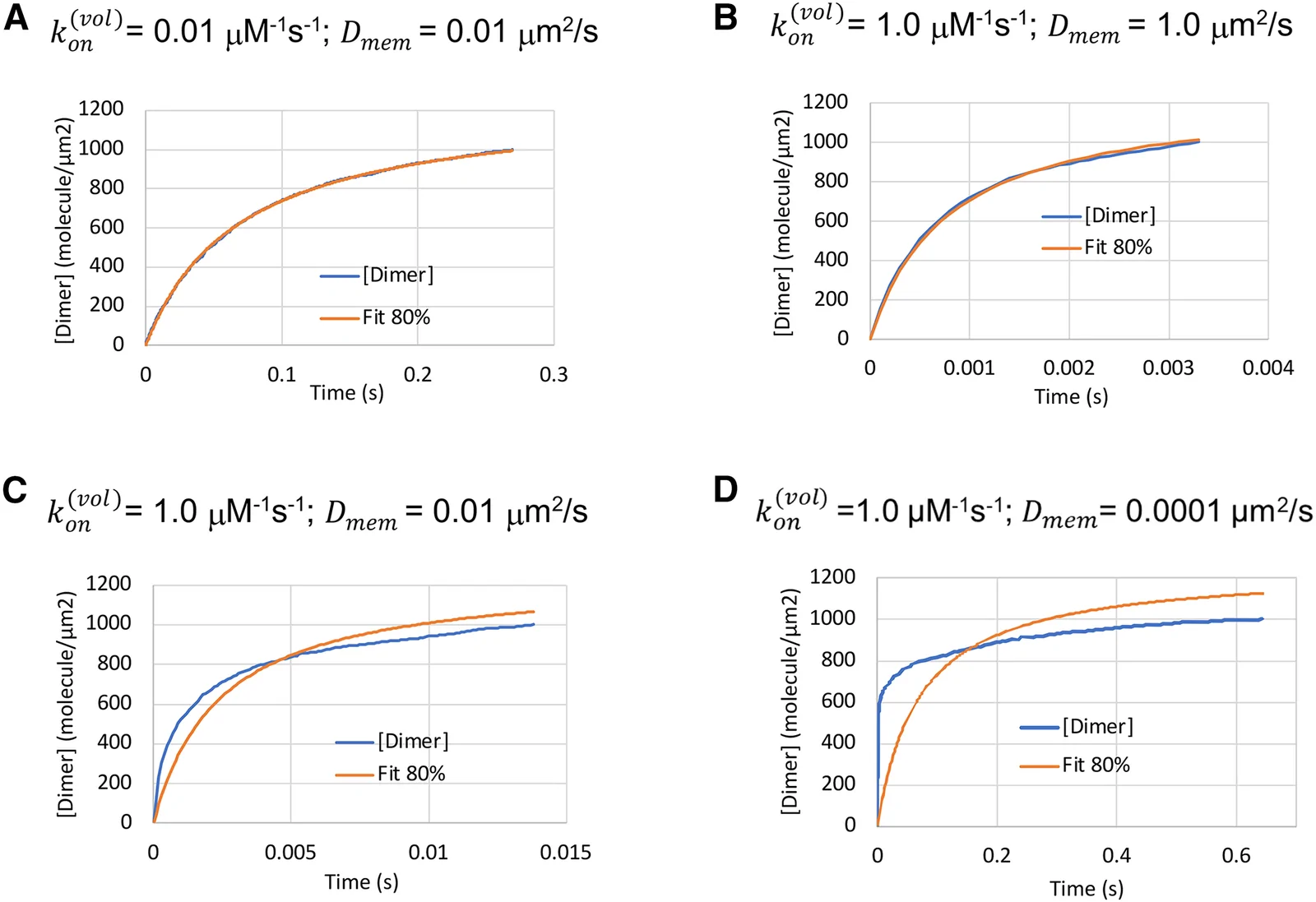
Examples of SpringSaLaD simulation data and their fit to surface-confined mass action dimerization kinetics. The initial surface density of monomers in each case is 2,500 molecule/μm^2^. The diffusion coefficient of the anchor and the volumetric binding rate constant of the binding sites are indicated above each graph, (A), (B), and (C) corresponding respectively to rows 1, 6, and 5 of Table 1. (D) shows a result with diffusion-limited kinetics, D_mem_ = 0.0001 μm^2^/s, exemplifying the poor fit to mass action.

**Figure 3:**
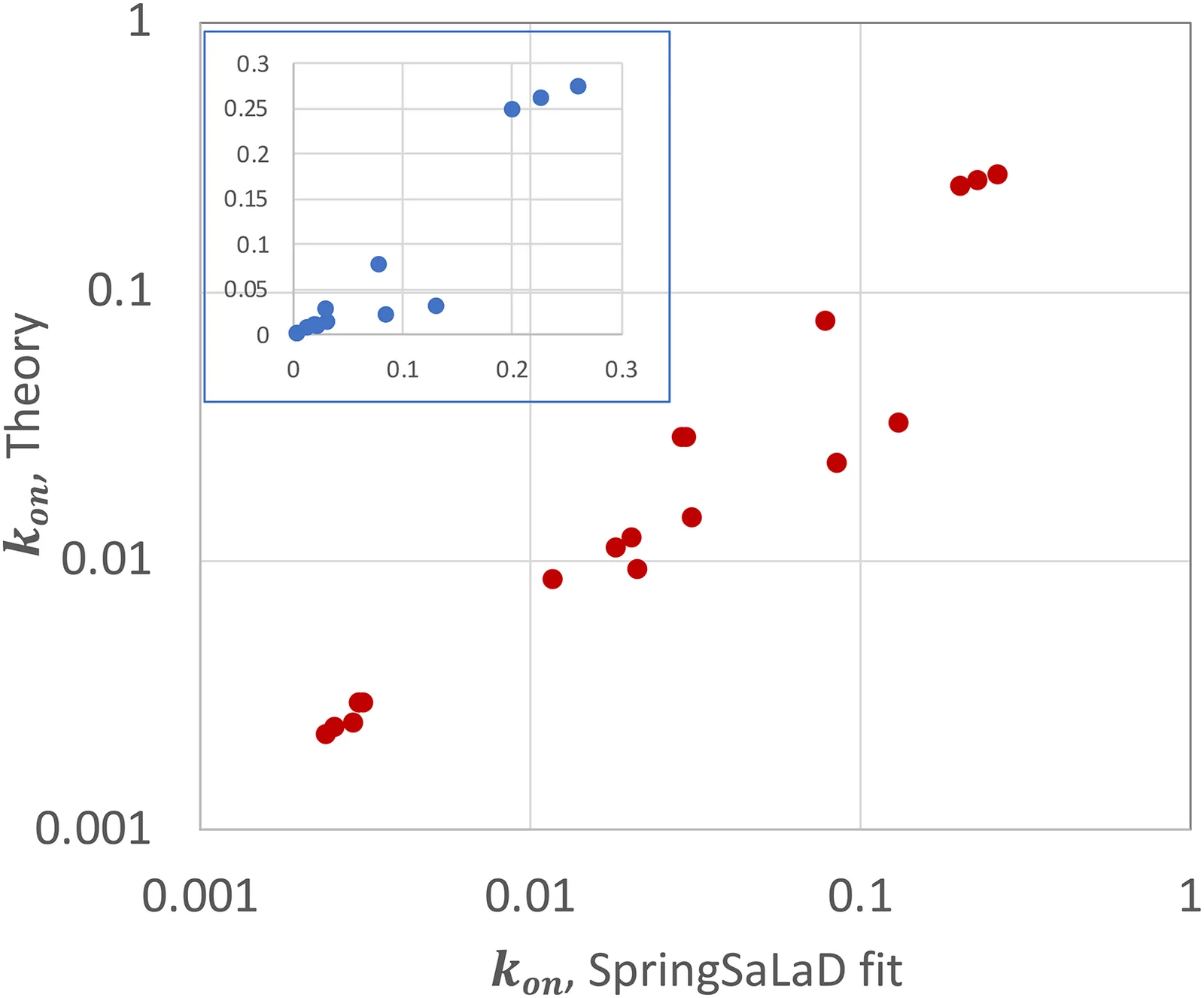
Correlation between $\bar{k}$ and $k_{on}^{\left(mem\right)}$ for 19 different conditions. The main plot is log-log to display the full range of rate constants; the insert is a linear plot. Full lists of all the additional conditions are in Table S2 of Supporting Text II.

All simulations were performed with SpringSaLaD v. 2.3.4 (https://vcell.org/ssalad). While the full physics and math behind the calculations performed by SpringSaLaD can be found in the original publication,^28^ we provide a brief overview of its functionality. A molecule in SpringSaLaD is approximated as a series of variably sized hard spheres, representing protein domains, connected by stiff springs. Random forces (chosen from a normal distribution with a variance determined from the diffusion coefficient assigned to each sphere by the user) impinge on each sphere at each time step and are transmitted to neighboring spheres through the stiff springs. The software models membranes as a planar surface within the volume of the 3D simulation domain. The membrane can have embedded spheres that serve as diffusing anchors (with user-assigned 2D diffusion coefficients) linked to spheres that extend into the volumetric space. Each sphere may also be designated as the site of a reaction. The user can input macroscopic rate constants for reactions, and the software converts these to reaction probabilities within each time step; for bimolecular reactions, this conversion also depends on the radii of the spherical binding sites and the sum of their diffusion coefficients. SpringSaLaD allows users to manually edit molecules to fully customize structure and molecule flexibility. The volumetric rate constant of binding between sites tethered to the membrane is assumed to be the same as that of the untethered sites. Geometrical and steric constraints at binding sites can be modeled by the appropriate placement of neighboring spheres adjacent to the respective binding sites within the reacting molecules. To build coarse-grained molecular structures used in SpringSaLaD for Figure 6, we used AlphaFold2^31^^,^^32^ to generate PDB file estimates of protein structures for EGFR, Grb2, SOS, and Ras via input of entire amino acid sequences. These PDB files are converted to highly coarse-grained molecular models via the mol2sphere^33^ utility embedded in SpringSaLaD. In some cases, we manually edited the structures to capture their essential features from measurements on the PDB structures, as visualized in PyMol (Schrödinger, Inc.). In particular, for SOS we subdivide the CDC25 and REM domains into multiple smaller spherical sites with only two sites (the allosteric and catalytic sites for Ras) capable of participating in a binding reaction. This process maintains the structural characteristics of these domains, while ensuring that the binding radius of the domain is not artificially inflated. Modeling disordered regions, such as the proline rich motif (PRM) region of SOS, can be challenging due to low confidence in the AlphaFold2-generated geometry of these regions. To model disordered domains, we use PyMOL to measure the length of entire straight chain amino acid sequences and then model this sequence in SpringSaLaD using 1.0-nm-diameter sites connected by 3.1-nm linkers (in SpringSaLaD, a string of relatively small spheres connected by links that are larger than the sphere diameters can be used to model flexible [i.e., disordered] domains). Binding reactions in all simulations have rates input in terms of μM^−1^s^−1^; in our simulations, successful binding results in 1-nm links between the surfaces of the spherical sites. The default simulation time step of 10 ns was sufficiently accurate for all the SpringSaLaD simulations in this work, as checked by testing 2-ns timesteps and noting no significant differences; we recommend this test be applied by all users, at least for the simulation scenarios with the fastest rates. Simulations for Tables 1 and 2 and Figure 1, Figure 2, Figure 3 and 5, were run with 40 molecules, while Figure 6 used 200 molecules of Ras and 20 complexes of EGFR-Grb2-SOS to proportionally reflect the difference in concentrations between Ras and SOS molecules *in vivo*.^34^^,^^35^ The 3D computational domain had a z coordinate of 100 nm, with the membrane placed in the XY plane at Z = 10 nm; the boundaries at the edges of the domain have reflective boundary conditions. X and Y coordinates were set to provide the desired initial surface densities. Initial placement of the molecules was random and different for each trajectory. A set of 100 trajectories is simulated in parallel using the Center for Cell Analysis and Modeling High Performance Compute Cluster (https://health.uconn.edu/high-performance-computing/resources/). One run for Tables 1 and 2 required approximately 3 h, and one run for Figure 6 required approximately 30 h. SpringSaLaD input files are in supplemental information and provide all the geometric details for the molecules in each computational experiment.

**Table 1:** Results for single stiff link, binding site *D*_*vol*_ = 1.0 μm^2^/s, *h* = 0.0055 μm:

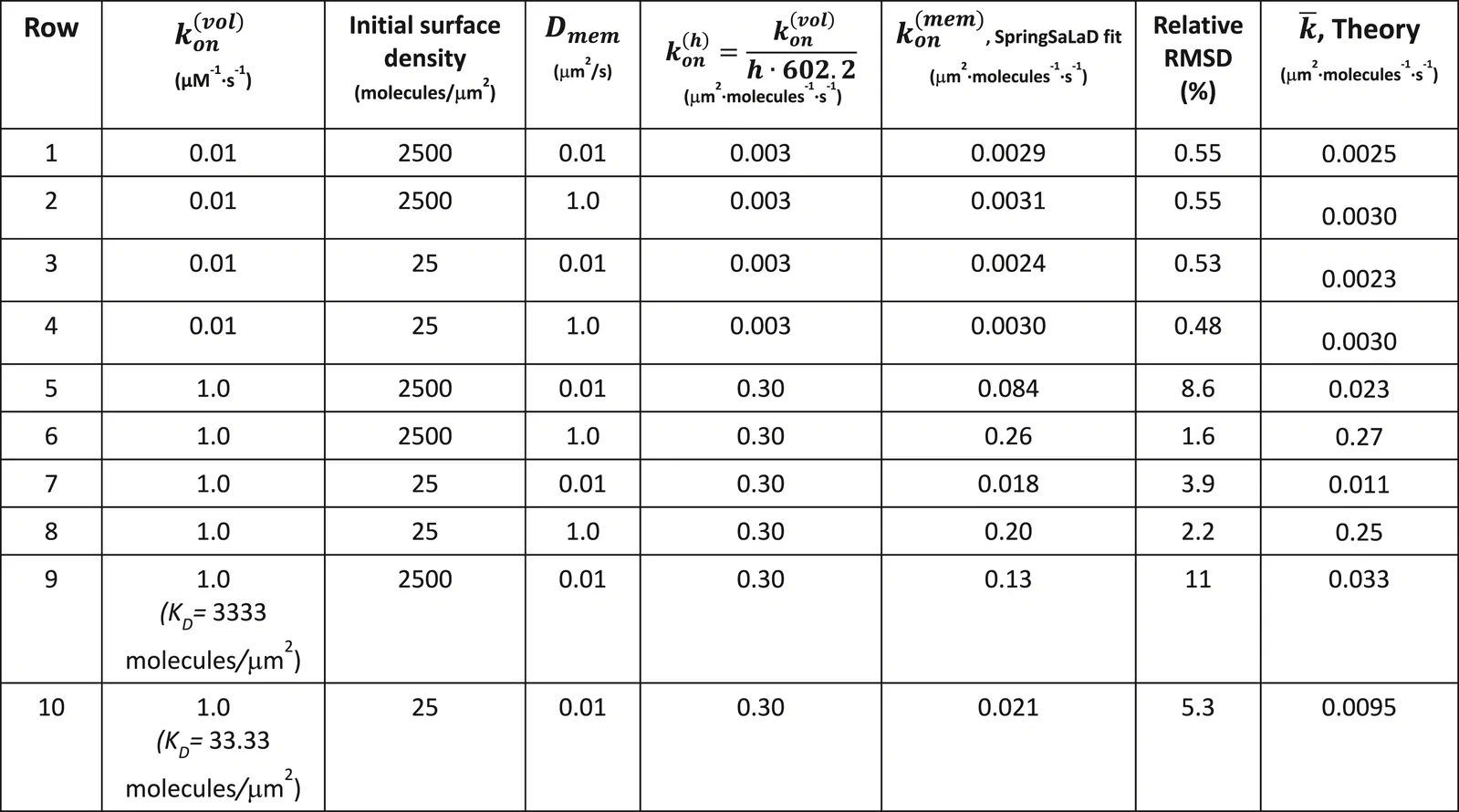

**Table 2:** Dimerization of idealized structure types

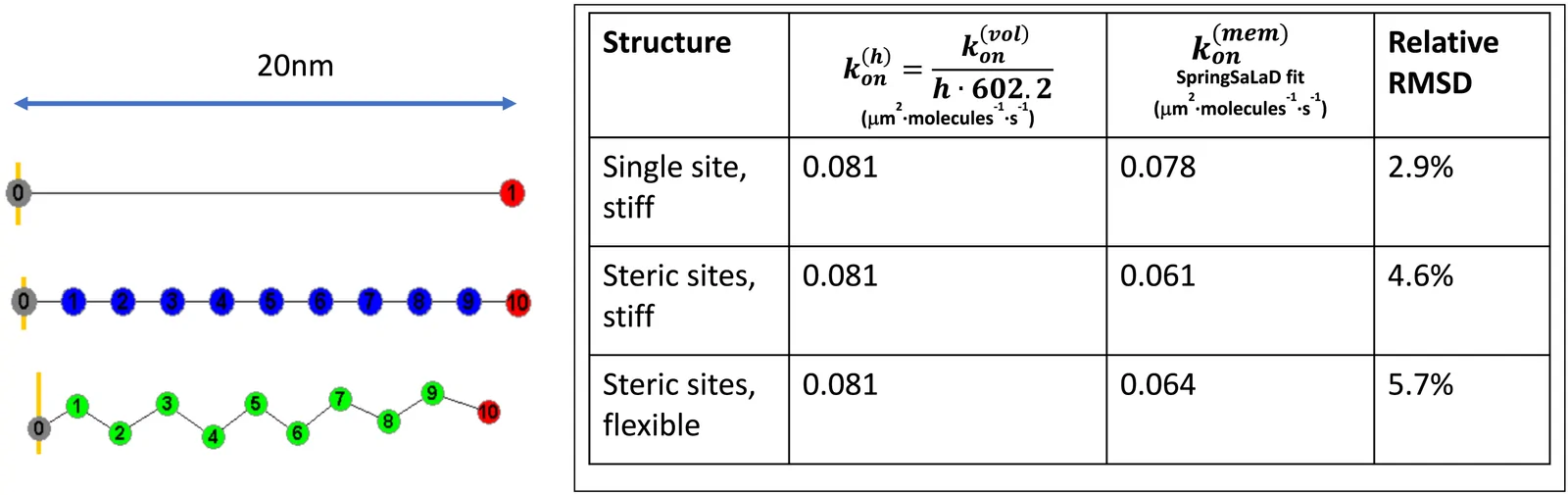

*D_mem_* =0.01μm^2^/s, binding site *D_vol_* =1.0μm^2^/s, $k_{on}^{\left(vol\right)}$ = 1 μM ^-1^s^-1^, *h*=0.0205 μm, surface density = 2500 molecules/μm^2^.

The mean time dependence of dimer surface density [*dimer*]_*t*_, based on 100 SpringSaLaD runs for each of the parameter sets in Tables 1 and 2 and Figure 6, was fitted with a deterministic 2D mass action rate law to obtain $k_{on}^{\left(mem\right)}$. For irreversible dimerization (Table 1, first 8 rows; Table 2), a fit to the analytical mass action expression for the appearance of dimer (Equation 1) was obtained with the Excel solver.

(Equation 1) $${\left[dimer\right]}_{t}={\left[dimer\right]}_{\infty }-\frac{{\left[dimer\right]}_{\infty }}{\left(4*k_{on}^{\left(mem\right)}*{\left[dimer\right]}_{\infty }*t\right)+1}$$

The values of $k_{on}^{\left(mem\right)}$ were all confirmed with COPASI.^36^ COPASI also provided the standard deviations (SDs) of the derived rate constants, which were all less than 0.5% of the determined $k_{on}^{\left(mem\right)}$. Importantly, this SD only estimates the uncertainty in $k_{on}^{\left(mem\right)}$ related to the finite curvature of the objective function near its minimum—not how well the simulation results are fit by the mass action model. The goodness of fit is characterized by the minimum of the objective function. In our study, the objective function was defined as the relative root mean squared deviation (RMSD) of the mass action equation (Equation 1) from the dimer surface density yielded by SpringSaLaD, as described in the results. For reversible dimerization (Table 1, rows 9 and 10) and for the fits in Figure 6, we used the COPASI parameter estimation tool within Virtual Cell (VCell)^37^^,^^38^ as well as COPASI itself for confirmation and to determine the SD. The former can be accessed in the VCell published BioModel “Peterson Table 1 reversible.” All these results with some further analysis can also be found in the spreadsheets included in the supplemental information. The VCell model related to Figure 6 can be found in the VCell database with the name “Peterson Figure 6: Ras-SOS_Binding_fit_to_SpringSaLaD.” The model related to Figure 7 can be available in the VCell database as “SOS_Recruitment_Ras_Binding”. Access to the VCell database is through the VCell client, which can be downloaded at https://vcell.org/run-vcell-software.

## Results

### The general approach

For bimolecular reactions, SpringSaLaD determines the microscopic probability of two binding sites forming a bond as they diffuse within a reaction radius that is slightly larger than the sum of their physical radii. The input to the algorithm is simply the macroscopic volumetric on-rate constant ($k_{on}^{\left(vol\right)}$, units of μM^−1^s^−1^) and the diffusion coefficient of the individual spheres. Full details on the derivation of the reaction probability and a thorough validation of its accuracy can be found in the original paper describing SpringSaLaD.^28^ Importantly for the purposes of this work, the rate of binding for sites that happen to be tethered to a membrane is still treated as volumetric, because the spherical sites are located in the volume compartment even while they are constrained with links to the 2D membrane surface.

Figure 1 illustrates this for a simple dimerization reaction where the two yellow binding sites are tethered to the membrane anchor (gray sphere) by a 5-nm link; in these simulations, both the anchor and tether sites are given identical diffusion coefficients of 1 μm^2^/s. We ran 100 SpringSaLaD simulations each with 40 dimerizing molecules (Figure 1 only shows two molecules for clarity). The mean trajectory for these 100 runs is then fitted with a deterministic mass action membrane binding model in terms of surface densities using either COPASI or Virtual Cell (although an analytical solution can also be fit for simple dimerization) (step 2 in Figure 1). The output binding rate constant, $k_{on}^{\left(mem\right)}$ in units of μm^2^molecule^−1^s^−1^, can then be used to parameterize larger deterministic or stochastic models (that utilize mass action rate constants as inputs) with molecule numbers (>1000) or timescales (>10 s) that would be too large for even highly coarse-grained molecular simulators like SpringSaLaD. Also, we emphasize that binding rates are typically determined experimentally using *in vitro* volumetric measurements; that SpringSaLaD uses volumetric rate constant inputs makes the procedure in Figure 1 especially appropriate and convenient. The results in Figure 1 show that a 2-nm-diameter binding site with an on-rate constant for dimerization of 0.47 μM^−1^s^−1^ and tethered to a membrane surface through a 5-nm link can be modeled as a 2D surface reaction with an on-rate constant, $k_{on}^{\left(mem\right)}$_,_ of 0.109 μm^2^molecule^−1^s^−1^. Using idealized models, we now explore how various structural and biophysical parameters control $k_{on}^{\left(mem\right)}$ and when mass action rate constants may not be appropriate to describe membrane-associated binding kinetics.

### Validation

To develop a rigorous validation of the method, we needed to devise an idealized system that would be theoretically tractable and also qualitatively intuitive. The goal was to test the procedure described in the previous section for various combinations of volumetric dimerization on-rate constant, 2D diffusion constantof the membrane anchor, and initial surface density.

To achieve this, we analyze the case of a 5-nm stiff tether between a 1-nm-diameter binding sphere and a membrane anchor site. The diffusion coefficient for the binder sphere is set to 1 μm^2^/s. Table 1 gives results for all combinations of two volumetric on-rate constants, two surface densities, and two membrane diffusion coefficients. All the dimerization rate laws are irreversible except for the last two rows, where the equilibrium dissociation constant, *K*_*D*_, is indicated. The 2D on-rate constant derived by fitting the SpringSaLaD simulation, $k_{on}^{\left(mem\right)}$, is in the fifth column. For consistency, all these fits are performed for kinetics at 80% completion (for practical applications of our method, shorter or longer levels of completion may be more appropriate, as discussed below). For comparison, we also provide the 2D on-rate constant, $k_{on}^{\left(h\right)}=k_{on}^{\left(vol\right)}/h$, where *h* is the linker length plus the radius of the binder sphere. If *h* is in units of μm, dividing by a unit conversion factor of 602.2 converts $k_{on}^{\left(h\right)}$ from units of μM^−1^s^−1^ to units of μm^2^molecules^−1^ s^−1^; $k_{on}^{\left(h\right)}$ is an equivalent 2D binding rate constant of freely diffusing monomers confined within a thin volume with a height *h* adjacent to the membrane. The Relative RMSD column provides the square root of the sum of the squared deviations of the fitted values from the SpringSaLaD values divided by the sum of the squared SpringSaLaD values; this metric provides a measure of the goodness of fit to the bimolecular mass action rate law and was minimized to obtain $k_{on}^{\left(mem\right)}$. Examples of the fits are shown in Figure 2. (The data and plotted fits for all the entries in Table 1 can be found in Supporting file “Peterson Table 1 Fits.xlsx.”) The last column, $\bar{k}$ is based on an analysis of the time-weighted average of the solution to the Smoluchowski theory for the tethered binding site anchored to the membrane; it will be discussed further, below.

#### The SpringSaLaD fits for reaction-limited binding are consistent with mass action

Let us start by considering the first 4 rows of Table 1 where the volumetric on-rate constants $k_{on}^{\left(vol\right)}$ (and therefore $k_{on}^{\left(h\right)}\;)$ are slow; these represent reaction-limited scenarios where diffusion in the membrane should not severely affect the kinetics, and therefore, mass action should be a good approximation. We looked at two anchor diffusion coefficients corresponding to that of a large transmembrane protein domain (*D*_*mem*_ = 0.01 μm^2^/s) and a lipid anchor (*D*_*mem*_ = 1 μm^2^/s); in all cases, the diffusion coefficient of the binder *D*_*vol*_ = 1 μm^2^/s. Thus, for the cases where *D*_*mem*_ = 0.01 μm^2^/s, the anchor effectively acts as a pivot, and the binder rapidly moves within a hemispherical shell to effectively create a reaction region with a thickness slightly larger than the 1-nm diameter of the binder sphere. Because the region of spatial overlap of the two shells where the binding may occur is restricted, it might seem surprising that $k_{on}^{\left(mem\right)}$ is so closely approximated by $k_{on}^{\left(h\right)}$ in the first row of Table 1. However, as detailed in the Supporting Text I, the reaction probability is enhanced because of the effectively higher density of binding sites within this restricted region, which compensates for the smaller spatial overlap. Thus, the calculations in the Supporting Text I both explain and validate the SpringSaLaD results for the cases of slow anchor diffusion. The second row of Table 1 corresponds to the case where the anchor and the binder have the same fast diffusion. In this scenario, that $k_{on}^{\left(mem\right)}$ ≅ $k_{on}^{\left(h\right)}$ is intuitive, because the effect of the tether in this case essentially reduces to confining the binders within the layer adjacent to the membrane.

#### When diffusion becomes limiting, $k_{on}^{\left(mem\right)}$ is dependent on initial surface density and deviates from $k_{on}^{\left(h\right)}$

Of course, when diffusion is limiting, it is known that the rates of membrane reactions are susceptible to deviations from a simple mass action rate law^10^^,^^12^^,^^16^^,^^39^^,^^40^^,^^41^^,^^42^^,^^43^; this can manifest itself as significant changes of the apparent rate constant as a function of initial surface density. We probed for this by decreasing the initial density by a factor of 100. The combinations of *D*_*mem*_ and $k_{on}^{\left(vol\right)}$ in the third and fourth rows of Table 1 resulted in relatively small changes in $k_{on}^{\left(mem\right)}$, indicating that the mass action rate law applies to these cases. To further test our approach when diffusion may become limiting, we increased the $k_{on}^{\left(vol\right)}$ by a factor of 100 in the lower half of Table 1. Clearly, for the case of *D*_*mem*_ = 0.01 μm^2^/s, there is a strong dependence on surface density (Table 1 rows 5 and 7; see also the fit in Figure 2C). The, $k_{on}^{\left(mem\right)}$ values for these 2 cases are very different from each other and also from $k_{on}^{\left(h\right)}$.Thus, the combination of $k_{on}^{\left(vol\right)}$ = 1.0 μM^−1^s^−1^ and *D*_*mem*_ = 0.01 μm^2^/s present cases where a simple mass action rate law may not be appropriate; this is even more apparent for the case of *D*_*mem*_ = 0.0001 μm^2^/s in Figure 2D, where the deviation of the SpringSaLaD simulation from the best fit to mass action dimerization is very severe. The simulation results are initially faster and then ultimately slower than the best fit that assumes mass action kinetics. This is because the monomers whose binding sites are initially close to each other (effectively within the “reach” of the tether) will react, but leave behind depletion zones where monomers are too far away from potential binding partners.^41^^,^^42^ These isolated monomers can be discerned toward the middle of Video S1, which presents an example trajectory for the first 150 ms of the highly diffusion-controlled case of Figure 2D. It starts with 40 monomers randomly placed on a 160 nm × 100 nm membrane (corresponding to an initial density of 2,500 monomers/μm^2^). It shows that the first 7 dimers form within the first 2 ms and correspond to the pairs of monomers that are relatively close to each other (i.e., “within reach”) at time 0 s. The slow anchor diffusion keeps the remaining 26 monomers from encounters, until by 20 ms, only 2 more dimers are formed, after which long-lived “depletion zones” become established; by the end of the remaining 130 ms in the movie, only 3 more dimers have formed. Given that this is only one trajectory, it is remarkably consistent with the mean of 100 trajectories in Figure 2D and that there are 2 timescales corresponding to approximately reaction limited at the very beginning and diffusion limited for most of the time course—decidedly not well described by Equation 1. But as will be discussed below, it might still be a reasonable approximation to use a mass action rate expression within a kinetic network model for a given initial condition and desired level of completion. Importantly, these results reflect the ability of the SpringSaLaD simulations to capture the physics of membrane-tethered reactions.

#### SpringSaLaD simulations are consistent with the thermodynamic law of mass action

In the last 2 rows of Table 1, we considered reversible reactions for the diffusion-limited scenario of fast $k_{on}^{\left(vol\right)}$ (1.0 μM^−1^s^−1^) and slow *D*_*mem*_ (0.01 μm^2^/s). For the SpringSaLaD simulations, we chose volumetric dissociation rates, $k_{off}^{vol}$, of 1,000 s^−1^ and 10 s^−1^, respectively, for the 2,500 and 25 monomer/μm^2^ initial densities. The respective mean steady-state densities of dimers in the SpringSaLaD simulations were 570 and 5.7 molecules/μm^2^ (see the full set of data and fits in the spreadsheet Peterson Table 1 Fits.xlsx). These values are consistent with the *thermodynamic* law of mass action^11^ and thus further validate the SpringSaLaD simulations. Namely, the ratio of the squared monomer surface density to the dimer surface density is equal at steady state to the equilibrium dissociation constant *K*_*D*_, which is determined by the strength of the interaction between the monomers and thus is independent of reaction kinetics. *K*_*D*_ in each case is thus equal to the ratio of the intrinsic on and off reaction rate constants, uninfluenced by diffusion. In our case, ${K_{D}=k_{off}^{vol}/k}_{on}^{\left(h\right)}$, because the interactions of the binding sites in the 3D volume above the membrane obey mass action kinetics, and $k_{on}^{\left(h\right)}$ is the 3D association rate constant adjusted for the restricted volume above the membrane. It is gratifying (and further validation) that the SpringSaLaD simulations produce these correct steady-state surface densities even when, as in the last 2 rows of Table 1, the kinetics are far from reaction limited.

#### Dimerization rates are highly sensitive to the linker length

Table 2 provides results for three computational experiments in which structural features of the SpringSaLaD molecules are varied. These are all for dimerization reactions where the maximum distance between the membrane and the binding site is four times longer than in Table 1: *h* = 0.0205 μm (linker length of 20 nm and binding site radius of 0.5 nm). The $k_{on}^{\left(h\right)}$ is therefore a factor of ∼4 slower than that in Table 1 for the same $k_{on}^{\left(vol\right)}$ of 1 μM^−1^s^−1^. The SpringSaLaD simulations were carried out with a slow membrane-anchor diffusion coefficient, *D*_*mem*_ = 0.01 μm^2^/s, to model a transmembrane protein domain. However, the longer reach makes the slow anchor diffusion less limiting, and we find that $k_{on}^{\left(mem\right)}$ is much closer to $k_{on}^{\left(h\right)}$ than the comparable condition of row 5 in Table 1. This idea is quantitatively analyzed in supporting textI I and will be further discussed below. The second row in Table 2 shows results for a structure where additional spherical sites are introduced between the membrane anchor and the binding site to model the space occupied by a cytosolic protein sequence; this steric effect results in a small decrease in $k_{on}^{\left(mem\right)}$. This decrease is diminished when flexibility is introduced by allowing the spherical sites to be pivot points in the third row of Table 2; this is how disordered domains may be modeled in SpringSaLaD. Overall, for these idealized structures and simple dimerization, the fitted on-rate constants in Table 2 are relatively close to $k_{on}^{\left(h\right)}$.

#### $k_{on}^{\left(mem\right)}$ correlates well with $\bar{k}$, derived from Smoluchowski theory for dimerization of membrane-tethered binding sites

Qualitatively, one can consider the overall rate coefficient, *k*_*on*_, to be affected by the intrinsic binding occurring once the sites are close enough to bind and characterized by the rate constant *k*_0_, and by diffusion-influenced reactant encounters characterized by their corresponding rate coefficient *k*_*D*_. In 3D, *k*_*D*_ can be treated as essentially time independent (see detailed discussion and Figure S7A in Supporting Text, part II),^14^ and so is *k*_*on*_, which in this case is computed as $k_{on}=\frac{k_{D}k_{0}}{k_{D}+k_{0}}$.^15^ As discussed above, simple mass action kinetics are not appropriate for 2D bimolecular reactions within a surface because *k*_*D*_ becomes a function of time.^10^^,^^12^^,^^14^^,^^15^ Specifically, in the diffusion-limited regimes with *k*_*D*_(*t*)≪*k*_0_, the *k*_*on*_ will be largely determined by *k*_*D*_(*t*) and thus will depend on time. For the idealized case of a single binding site tethered to the membrane by a single link, as in Table 1 and the first row of Table 2, we were able to adapt Smoluchowski theory for the rate of diffusion-limited encounters in 2D^10^^,^^12^^,^^14^^,^^15^ to derive *k*_*D*_*(t)* for the case of tethered binders, as detailed in Supporting Text II. With *k*_*D*_*(t)* in hand for any *D*_*mem*_, and taking the intrinsic reaction rate constant to be $k_{on}^{\left(h\right)}$, we could derive a theoretical time-weighted average rate constant, $\bar{k}$, for a desired level of completion (i.e., $\bar{k}\left(t_{c}\right)\text{,}$ where *t*_*c*_ is the time it takes to reach the level of completion); see the derivation leading up to Equation S14 in Supporting Text II. Figure 3 shows there is a good correlation between $\bar{k}$ and $k_{on}^{\left(mem\right)}$; Figure 3 includes all 10 cases in Table 1, an additional 8 intermediate cases of surface density and *D*_*mem*_, and the first row of Table 2. This is all the more remarkable because different theoretical approaches to determine $\bar{k}$ were required for, respectively, the irreversible 5-nm structures (16 conditions), the irreversible 20-nm structure (Table 2, first row), and the 2 reversible 5-nm cases (Table 1, rows 9 and 10); all of these are described in Supporting Text II.

### Considerations for using $k_{on}^{\left(mem\right)}$ in models of cell signaling

The previous section examined the rate constant, $k_{on}^{\left(mem\right)}$, derived from the SpringSaLaD fits to Equation 1 in a variety of scenarios based on idealized structures. We showed that $k_{on}^{\left(mem\right)}$ follows theory, both qualitatively and quantitatively even for diffusion-limited reactions on membranes, where the results are far from the simple mass action rate law. In this section, we seek to understand how $k_{on}^{\left(mem\right)}$ may be a sufficiently good approximation to be useful in modeling cell signaling, even for situations where diffusion may influence the kinetics.

In a signaling model, the dynamic levels of molecular species feeds into other components of a reaction network. For each of the individual reactions in the network, if the concentrations of reactant and product are reasonably well estimated, the model can provide useful predictions. How well $k_{on}^{\left(mem\right)}$ for our mass action rate expression predicts the levels of reactant and product is given by the relative RMSD. Consider row 5 of Table 1. For this set of conditions, $k_{on}^{\left(mem\right)}$ (0.084 μm^2^molecule^−1^s^−1^) is significantly lower than $k_{on}^{\left(h\right)}$ (0.30 μm^2^molecule^−1^s^−1^), which can be taken as the rate coefficient in the limit of fast diffusion. Despite this, the fit to mass action kinetics appears to be reasonably good (Figure 2C) with a corresponding relative RMSD of 8.6%. While there is an underestimate of the fitted curve at short times and overestimate at long times, at any given time point, the estimated surface density of product is on average within 8.6% of the actual SpringSaLaD simulation. Therefore, even when diffusion severely influences the rate, we might be able to use the derived $k_{on}^{\left(mem\right)}$ within a signaling model, where an uncertainty of 10% is often quite acceptable. We will now further analyze why the relative RMSD can be small even when diffusion is limiting and the true rate coefficient is time dependent.

**Figure 4:**
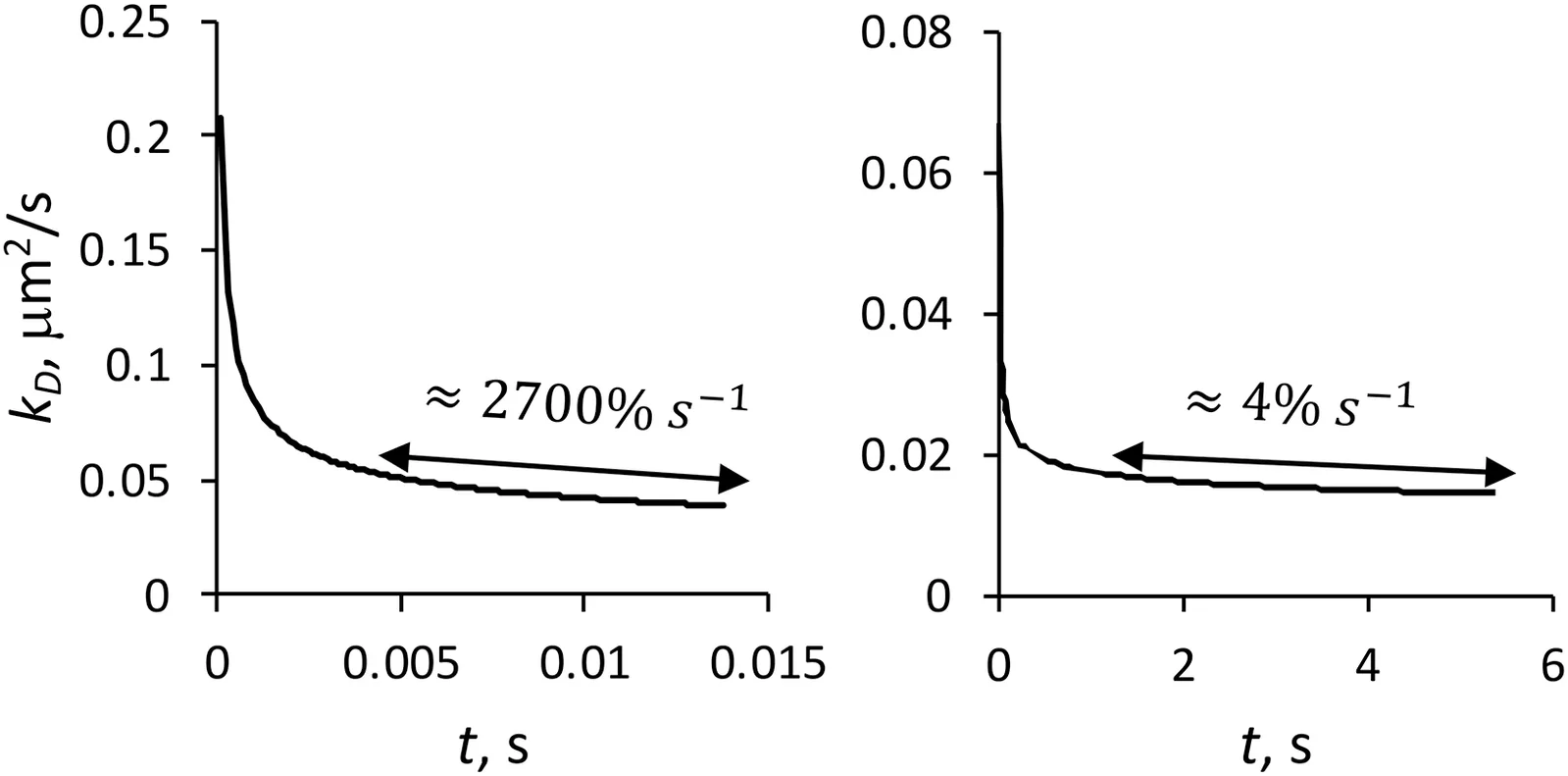
Time dependence of *k*_*D*_ in 2D. Time dependence of *k*_*D*_ in 2D for the conditions of rows 5 (*left*) and 7 (*right*). The abscissa is time in seconds.

The key to understanding the small relative RMSD for cases where diffusion is limiting lies in an examination of the time dependence of *k*_*D*_*(t).* This was derived from Smoluchowski theory for the idealized tethered binders in Supporting Text II and is illustrated in Figure 4 for the conditions of rows 5 and 7 of Table 1. We focus on rows 5 and 7 because the conditions are the same for each except for the initial concentrations (2,500 and 25 molecule/μm^2^, respectively); furthermore, their $k_{on}^{\left(mem\right)}$ values (0.084 and 0.018 μm^2^molecule^−1^s^−1^, respectively) are each quite far from $k_{on}^{\left(h\right)}$, indicating largely diffusion-limited kinetics. Strikingly, the relative RMSD for line 7 (3.9%) is smaller than for line 5 (8.6%) even though $k_{on}^{\left(mem\right)}$ for line 7 is much further from $k_{on}^{\left(h\right)}$. The results of Figure 4 show that most of the time dependence of *k*_*D*_*(t)* occurs during the very beginning of the kinetics. When the kinetics is lengthened simply by starting with lower initial surface densities (Figure 4, right panel), *k*_*D*_*(t)* stays relatively constant over much of the time course. This explains why the longer timescale associated with the kinetics of lower initial surface density results in a near constant contribution of *k*_*D*_*(t)* to the overall k_on_ and the reasonable fit to a constant $k_{on}^{\left(mem\right)}$ in Equation 1. Importantly, because $k_{on}^{\left(mem\right)}$ is so dependent on initial conditions when diffusion becomes limiting, these must be specified in the SpringSaLaD simulations and be based on the corresponding conditions in the network model that is being targeted for parametrization.

**Figure 5:**
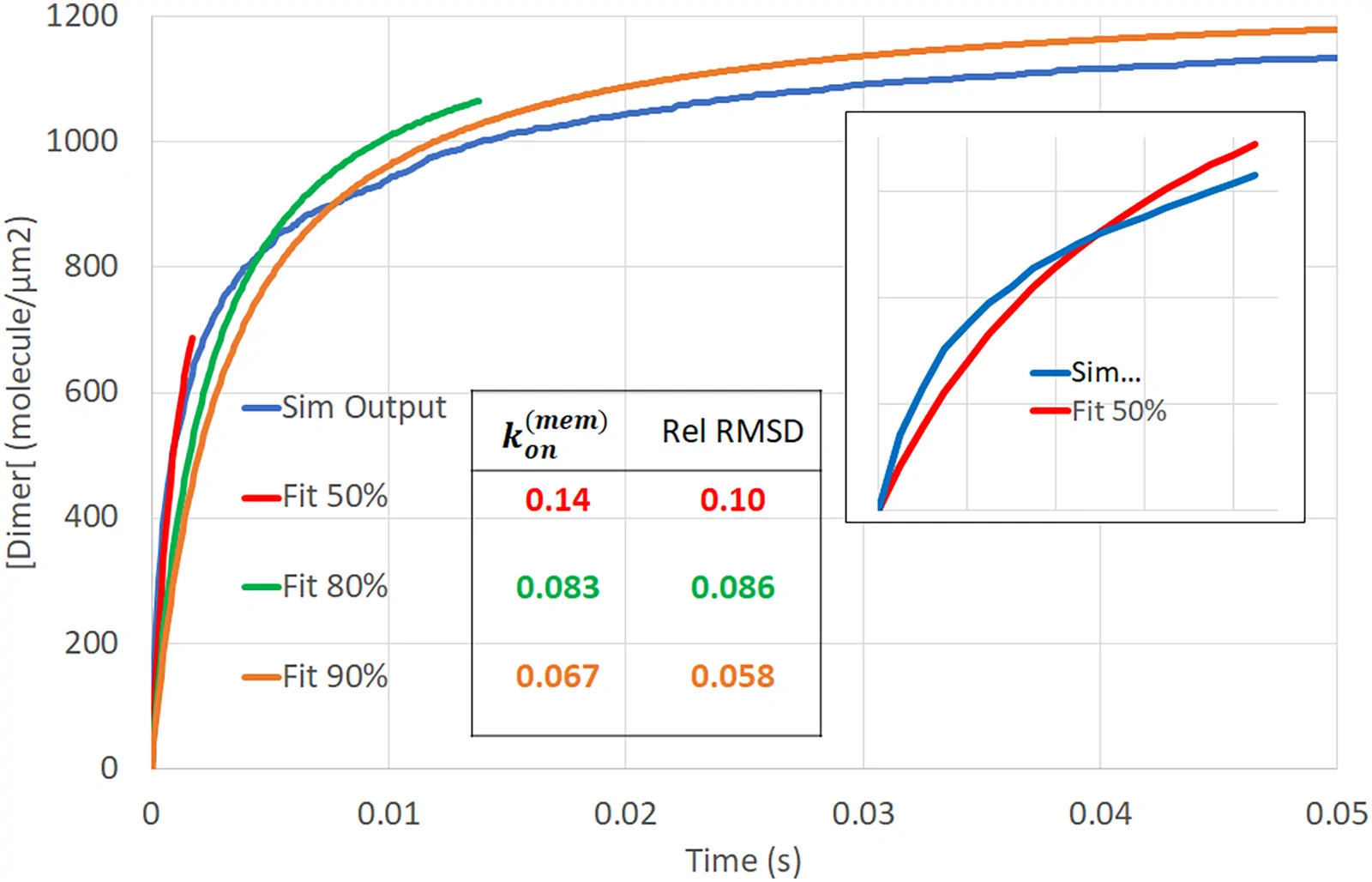
Fits to different levels of completion for the conditions of row 5 of Table 1. The inset is an expansion of the first 1.7 ms.

Thus far, we have compared all our results for the same level of completion—chosen arbitrarily at 80%. But, it is important to appreciate that $k_{on}^{\left(mem\right)}$ will indeed be sensitive to the level of completion for diffusion-influenced kinetics. This is intuitive based on the idea that at the initial stages more tethered binding sites will be close enough to react with a reaction-limited rate, but as “depletion zones” develop, diffusion will increasingly limit the rate of reaction (see the results described in relation to Figure 2D and Video S1). Figure 5 examines this issue directly for the diffusion-influenced case of row 5 in Table 1, where in addition to the fit to 80% completion, we also examined the fits to 50% and 90%. The earlier the fit is terminated, the closer is $k_{on}^{\left(mem\right)}$ to $k_{on}^{\left(h\right)}$, the reaction-limited rate. On the other hand, the fits out to longer levels of completion display better relative RMSD; this is, of course, consistent with our discussion of Figure 4, which shows that at longer times the diffusion-limited component of the rate coefficient varies very slowly. These considerations further underline the importance of specifying the pertinent conditions as precisely as possible when determining a diffusion-influenced $k_{on}^{\left(mem\right)}$ for use in a reaction network model.

But, let us return to the situation where the kinetics are essentially reaction limited (i.e., where the diffusion of the membrane anchor is fast on the timescale of the intrinsic reaction rate). This is the case for the first 4 rows of Table 1 and for Table 2, where $k_{on}^{\left(mem\right)}$ is close to $k_{on}^{\left(h\right)}$. In such cases, it should be possible to use a single value of $k_{on}^{\left(mem\right)}$ for a large range of initial surface densities and levels of completion. Of course, in Tables 1 and 2, $k_{on}^{\left(h\right)}$ was computed from the value of *h* assigned to these idealized structures. In a more realistic application of the method with coarse-grained molecular structures (e.g., in the next section), the value of *h* may be difficult to estimate; furthermore, the reaction may be between sites on different molecules (as opposed to homodimerization as in Tables 1 and 2). However, it should be possible to determine whether $k_{on}^{\left(mem\right)}$ is close to the reaction-limited rate constant simply based on the criteria outlined in this section: that it is insensitive to initial surface density and to the level of completion. Indeed, comparing row 2 to row 4 (where anchor diffusion is high) shows that a 100-fold decrease in surface density does not appreciably affect $k_{on}^{\left(mem\right)}$; however, there is a difference between rows 1 and 3, where the *D*_*mem*_ is 100-fold lower. Another criterion for how close $k_{on}^{\left(mem\right)}$ is to the reaction limit is its sensitivity to the level of completion; Figure 5 demonstrates a high sensitivity for the condition of fast $k_{on}^{\left(h\right)}$ and slow *D*_*mem*_. On the other hand, the opposite conditions in row 2 of Table 1 give the same $k_{on}^{\left(mem\right)}$ of 0.0031 for 50%, 80%, and 90% levels of completion (Supporting file Peterson Table 1 Fits.xlsx). Finally, a third test that could be performed is to see how sensitive the derived $k_{on}^{\left(mem\right)}$ is to a change in *D*_*mem*_; this is exemplified by comparing rows 1 and 2, which show that this scenario is insensitive to *D*_*mem*_ and therefore can be taken to be reaction limited (compare, on the other hand, rows 5 and 6). All of the above tests can be applied to complex coarse-grained structures where $k_{on}^{\left(h\right)}$ cannot be determined *a priori*.

We now illustrate our method by applying it to a real signaling module that has been widely investigated because of its importance in cancer biology.

### Application of the method to interaction of receptor-bound SOS with Ras

Until now, we have employed idealized molecular structures to validate our method and to learn more about biophysical principles that control the on-rate constants of binding sites tethered to membranes. We now illustrate the application of this approach to a biologically relevant example, namely the interaction of the GEF SOS with the lipid-anchored small G-protein Ras.^30^

SOS has two binding sites for Ras: an allosteric site and a catalytic site. When a Ras molecule binds to the allosteric site, it increases the GEF activity of the catalytic site.^44^ Additionally, before SOS binds to Ras, it is first recruited to an active receptor tyrosine kinase (RTK) through an adapter protein; the adaptor binds to a PRM on SOS via a SH3 domain and to a phosphorylated tyrosine via a SH2 domain. One such RTK is the EGFR, and one such adaptor protein is Grb2.^45^ Once SOS is bound to Grb2, it becomes membrane tethered, and its interaction with Ras is facilitated.^44^ However the complex mechanistic details of this system are still emerging.^46^

**Figure 6:**
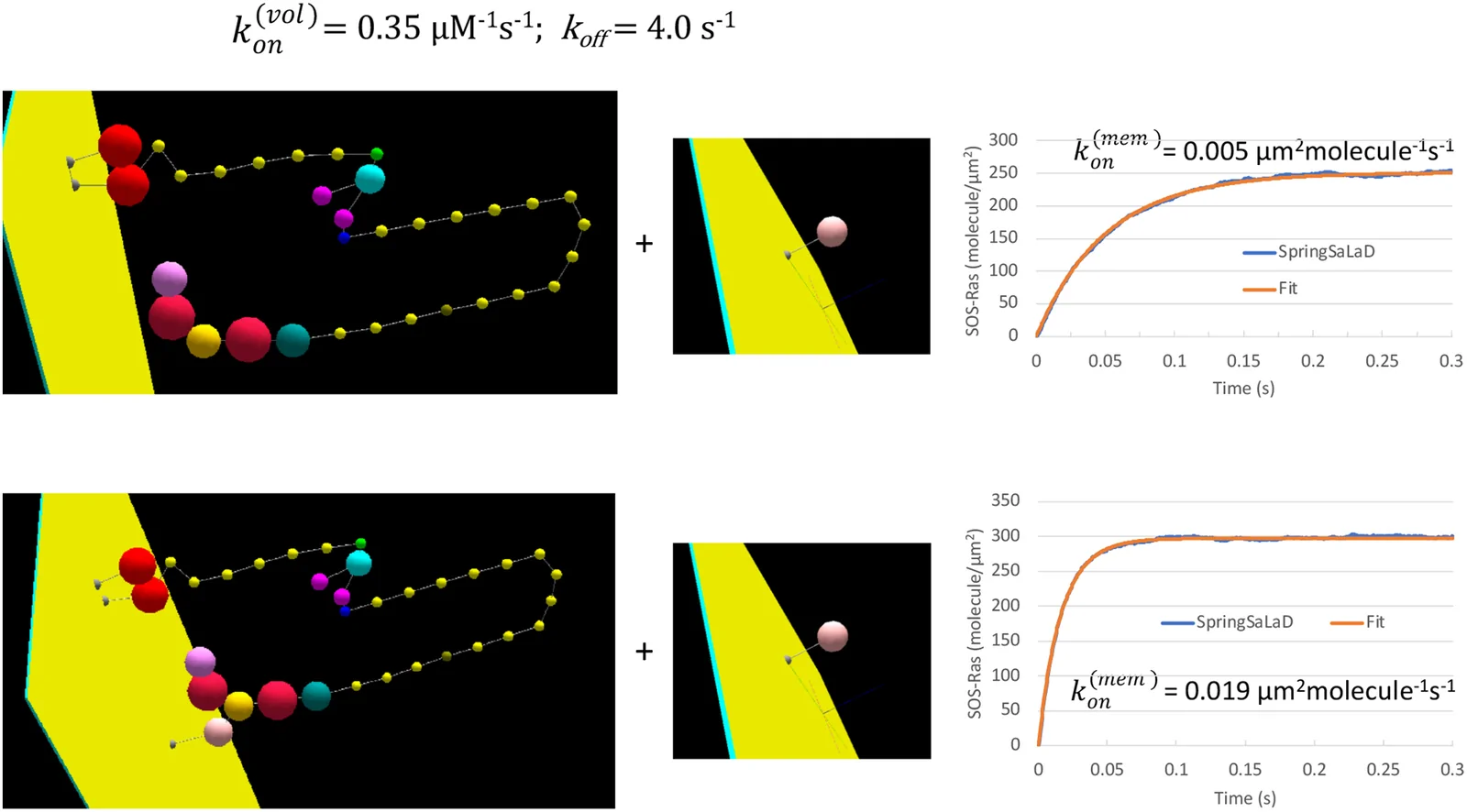
Membrane binding of Ras to the catalytic site of receptor-bound SOS. Top: direct binding. Bottom: after pre-binding of Ras to the allosteric site. The molecular structures are approximated by using the SpringSaLaD 3D editing utility based on atomic structures derived from AlphaFold2. The top left structure is an EGFR cytoplasmic domain anchored to the membrane (red kinase domain, followed by yellow disordered tail capped by a phosphotyrosine in green); the latter is linked to a cyan SH2 domain in Grb2; one of its magenta SH3 domains is linked to an olive PRM on the end of the disordered region of SOS; the violet SOS sphere is the binding site for the catalytic domain of Ras. The bottom left structure is identical, except that the pink allosteric site on SOS is prebound to Ras. The Ras structures are shown in the center with the pink spheres serving as the binding sites. The input rate constants for the SpringSaLaD simulations are shown at the top, corresponding to the volumetric on-rate constant for Ras binding to the catalytic site of SOS. The EGFR anchor diffusion coefficient is 0.01 μm^2^/s. All other site diffusion coefficients are 1.0 μm^2^/s. For each condition, 20 EGFR-Grb2-SOS molecules react with 200 Ras molecules on a 250 nm × 250 nm membrane surface to generate 100 SpringSaLaD trajectories. Their means were fitted to a deterministic 2D rate law to derive $k_{on}^{\left(mem\right)}$, fixing *k*_*off*_ at 4.0 s^−1^; results for the 2 conditions are shown on the right. The fits were carried out for the full 0.6-s simulations, which was close to steady state. For a Figure360 author presentation of this figure, see https://doi.org/10.1016/j.bpj.2026.04.015.

We asked the limited question of how binding of Ras with the receptor-associated SOS catalytic site might depend on whether SOS is prebound to Ras at the allosteric site. Ras is a lipid-anchored protein, so we reasoned that binding of Ras to the allosteric site of SOS would bring the SOS catalytic site closer to the membrane to enhance binding to a second Ras and subsequent exchange of GDP for GTP. Just how large an effect this is may be estimated by the procedure developed above, with the results shown in Figure 6.

We developed molecular models with the aid of the mol2sphere^33^ utility within SpringSaLaD and were guided by AlphaFold 2 atomic structure predictions^31^^,^^32^; all the site diameters and linker lengths are available in the SpringSaLaD input file included in the supplemental information; snapshots of the structure are shown in Figure 6. The top of Figure 6 displays results for binding of SOS-Grb2-EGFR to Ras at the SOS catalytic site; the bottom shows results for the same reaction, except SOS-Grb2-EGFR had been first bound to a Ras molecule at the SOS allosteric site. The input rates shown at the top of Figure 6 are based on experimentally measured data^30^ and are applied to both of the reactions. Special considerations were applied to ensure accuracy of the on-rate constant, which was parameterized via an initial cytosolic reaction where the output rate constant was matched to the experimentally measured rate data.^30^ This important step allows us to assure the SpringSaLaD volumetric on-rate constants reproduce measured reaction rates. The desired output rate corresponding to an experimentally observed rate constant of 0.27 μM^−1^s^−1^^30^ was achieved with a slightly adjusted input rate constant of 0.35 μM^−1^s^−1^, which was then used in subsequent simulations of membrane-bound interactions in Figure 6. In these models, the EGFR membrane anchor site is assigned a diffusion coefficient of 0.01 μm^2^/s to represent a large transmembrane protein, while the Ras membrane anchor is assigned a diffusion coefficient of 1.0 μm^2^/s to represent a lipid anchor; all the sites that are dangling in the cytosol volume are given *D*_*vol*_ of 1.0 μm^2^/s, but since the binding reaction is not close to diffusion-limited, the precise values are not critical. Consistent with these being reaction-limited on rates, the SpringSaLaD simulation outputs (averages of 100 runs) are very well fitted to reversible mass action kinetic law, as shown in the plots on the right of Figure 6 (relative RMSDs are, respectively, 1.6% and 0.6%). Additionally, as discussed in relation to Figure 5, $k_{on}^{\left(mem\right)}$ is insensitive to the level of completion for both cases (see supplemental information spreadsheet Ras_SOS fits for Figure 6), which is consistent with fully reaction-limited mass action kinetics. The 2D on-rate constants ($k_{on}^{\left(mem\right)}$) derived from these fits are, respectively, 5.0 × 10^−3^ μm^2^molecules^−1^s^−1^ and 1.9 × 10^−2^ μm^2^molecules^−1^s^−1^. Likewise, the affinity of the catalytic site is increased by allosteric site pre-association: *K*_*D*_ = 800 molecules/μm^2^ for the top of Figure 6 and 210 molecules/μm^2^ for the bottom pre-association case. Thus, SOS allosteric site association with Ras is estimated to enhance its catalytic site binding rate and affinity by a factor of ∼4—even when SOS is already confined to the membrane through Grb2-mediated association with EGFR.

### A simple ODE model of receptor-mediated signaling

**Figure 7:**
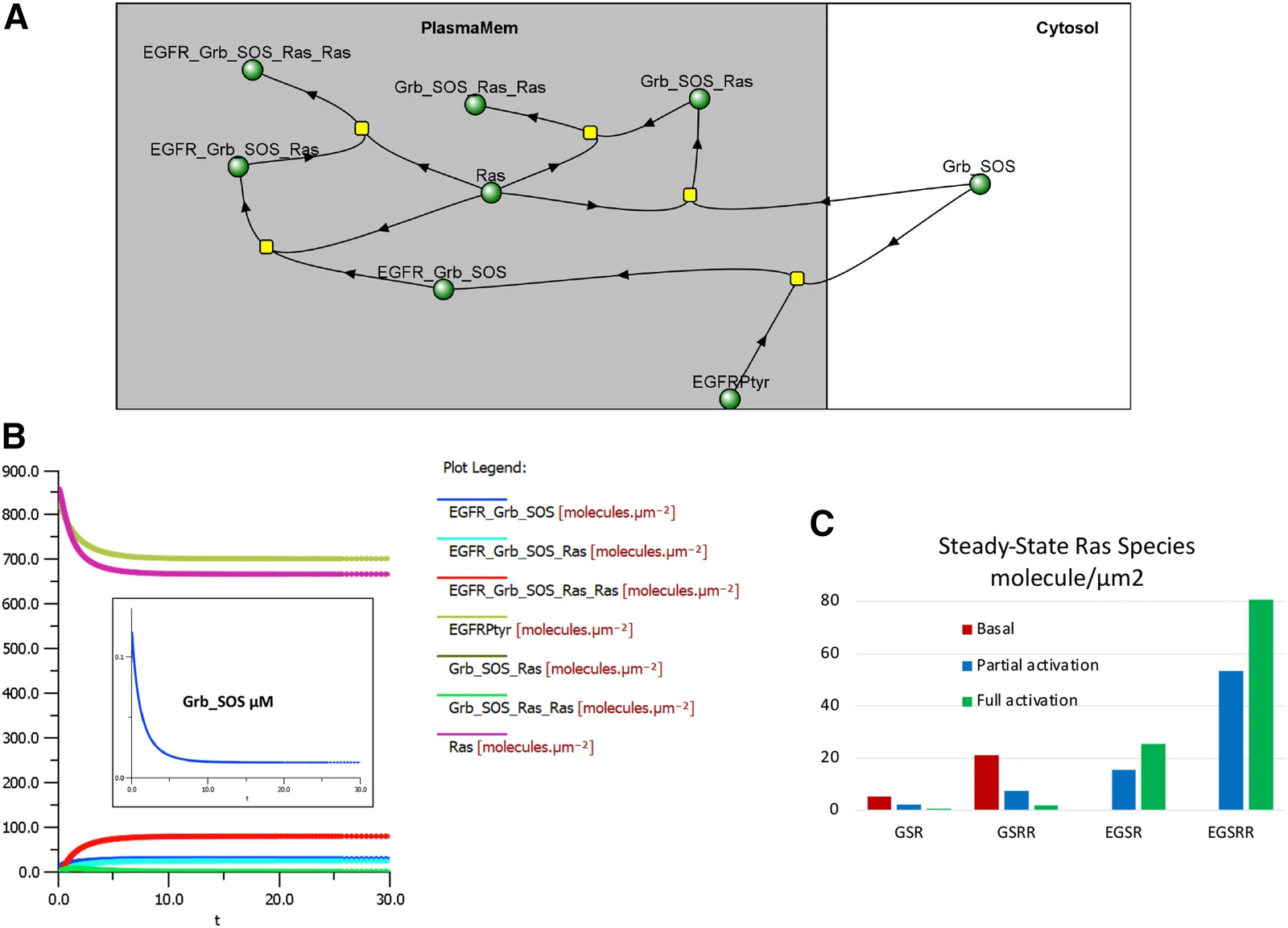
A simple VCell model and simulation results for EGFR signaling to Ras. (A) Reaction network identifying all the variables (see also the corresponding reactions in Table 3.). (B) The kinetics reach steady state within 30 s. The primary plot shows all the membrane species, and the inset shows Grb_SOS in the cytosol. Steady-state surface densities of the 4 different species of 1 or 2 Ras bound to Grb_SOS or EGFRPTyr_Grb_SOS under 3 conditions: “Basal,” “Partial activation,” and “Full activation” corresponding respectively to 0, 200, and 840 molecules/μm^2^ initial EGFRPTyr.

To illustrate the application of our approach, we built a small signaling model based on the values of $k_{on}^{\left(mem\right)}$ determined in the previous section. The ODE model was built in VCell,^37^^,^^38^ and total concentrations of the various species were from measurements on HeLa cells.^34^^,^^35^ In addition to the two reactions from Figure 6, we also modeled recruitment of Grb_SOS from the cytosol to the membrane by phosphorylated EGFR (EGFRPTyr) and direct stepwise binding of two Ras molecules to Grb_SOS; the model assumes that almost all the cytosolic SOS is prebound to Grb2, which is present in excess.^47^ The VCell reaction diagram in Figure 7A displays the connectivity of the network, and Table 3 provides the corresponding mass action rate constants.

**Table 3:** Rate constants for the VCell model

Reaction	*k*_*on*_	*k*_*off*_	Source
EGFRPTyr + Grb_SOS -> EGFR_Grb_SOS	1.0 μM^−1^s^−1^	0.3 s^−1^	Chook et al.^48^ 1996
Grb_SOS + Ras -> Grb_SOS_Ras	0.27 μM^−1^s^−1^	4.0 s^−1^	Iversen et al.^30^ 2014
EGFR_Grb_SOS + Ras -> EGFR_Grb_SOS_Ras	0.005 μm^2^molecule^−1^s^−1^	4.0 s^−1^	Iversen et al.^30^ 2014 with Figure 6
Ras + EGFR_Grb_SOS_Ras -> EGFR_Grb_SOS_Ras_Ras	0.019 μm^2^molecule^−1^s^−1^	4.0 s^−1^	Iversen et al.^30^ 2014 with Figure 6
Ras + Grb_SOS_Ras -> Grb_SOS_Ras_Ras	0.019 μm^2^molecule^−1^s^−1^	4.0 s^−1^	Iversen et al.^30^ 2014 with Figure 6

We used the data for EGFR measured in HeLa cells^34^ to derive an initial surface density for EGFPtyr of 840 molecules/μm^2^, which we took to be the fully activated receptor (we did not attempt to account for kinetics of inactivation, which would lower this number, or for the multiple phosphorylation sites available on the cytoplasmic domain of EGFR, which would raise this number). Figure 7B shows the approach to steady state for this “Full activation” condition. It shows that free EGFPtyr and free Ras are only partially depleted at steady state, while ∼90% Grb_SOS is removed from the cytosol to become membrane associated. Figure 7C provides a more detailed look at the association of Grb_SOS with Ras. We chose three levels of initial EGFRPTyr, 0, 200 and 840 molecules/μm^2^, to model, respectively, basal unstimulated cells, partial activation of EGFR, and full activation. The species composed of doubly bound Ras, Grb_SOS_Ras_Ras and EGFR_Grb_SOS_Ras_Ras (GSRR and EGSRR in Figure 5), are always a factor of ∼3.5 higher than the corresponding species with one Ras. This is important because the binding of two Ras molecules is required for SOS to optimally express its GEF activity^49^; the second binding event is, of course, significantly enhanced by the proximity effect quantified in Figure 6; this enhancement at the membrane has been reported based on experiments for the binding of Ras to SOS alone.^50^ Another conclusion from Figure 7C is that the overall levels of Ras doubly bound to SOS are significantly increased by EGFR activation; this is consistent with the high affinity of the SH2 domains on Grb2 for phosphotyrosine,^48^ creating a higher local concentration of SOS near the membrane surface. Indeed, under the “Basal” condition without participation of EGFRPTyr, 80% of the Grb_SOS remains unbound in the cytosol (data not shown but available in the public VCell model “SOS_Recruitment_Ras_Binding”).

## Discussion

The kinetics of reactions at membranes have long fascinated biophysicists.^8^^,^^10^^,^^12^^,^^13^^,^^17^^,^^21^^,^^27^^,^^40^^,^^51^ These studies have produced theoretical insights to illuminate how surface-associated reactions have distinct properties compared with reactions occurring in 3D solution. Which of these special properties are most pertinent to any given membrane-bound molecular interaction is difficult to ascertain *a priori*. Furthermore, experiments to measure bimolecular kinetics on membrane surfaces are complex,^6^^,^^7^ so often only on-rate constants measured in 3D are accessible. Fundamentally, however, the kinetics of key membrane-associated reactions depend on the 2D surface densities and 2D rate constants, not on the bulk cellular concentrations and 3D rate constants. Indeed, because surface to volume ratios of different cell types vary tremendously, volumetric rate constants cannot be readily used to model and simulate cell signaling systems. Furthermore, because bimolecular reactions in 2D may have a special dependence on diffusion, a single mass action rate constant may not be applicable. To address these theoretical and practical problems, we describe a procedure (Figure 1) using experimentally accessible volumetric on-rate constants, $k_{on}^{\left(vol\right)}$ within the SpringSaLaD simulation software to estimate the $k_{on}^{\left(mem\right)}$, the 2-dimensional rate constant for a membrane-confined bimolecular reaction. Of course, the ideal would be to perform full atomistic molecular dynamics simulation within a realistic lipid bilayer using inter-molecular potentials and force fields.^21^^,^^25^^,^^26^ But deriving typical binding kinetics would require prohibitively expensive multimolecular simulations on the seconds timescale. This is why more highly approximate simulations may be required. In our approach, the potentials are encapsulated in the experimentally determined on-rate constant, and intra- and inter-molecular dynamics are derived from diffusion coefficients using a Langevin dynamics formalism.^28^ While the molecular structures are necessarily highly coarse grained, we believe they capture key factors that govern reactivity, namely 2D diffusion, proximity of binding sites to the membrane, and the steric environment of the binding sites.

To validate the method of Figure 1, we applied it to the dimerization of a single binding site tethered to a surface through a 5-nm stiff linker, where the membrane anchor acts as a pivot (Table 1). For the situation where the reaction is rate limiting, this system can be solved theoretically (see Supporting Text I); gratifyingly, $k_{on}^{\left(mem\right)}$ determined by our method is well reproduced by this solution. Interestingly, for these cases, $k_{on}^{\left(mem\right)}$ is well approximated by $k_{on}^{\left(h\right)}=k_{on}^{\left(vol\right)}/h$/602.2 (μm^2^molecules^−1^s^−^^1^), where *h* is the distance of the binding site from the membrane anchor (in μm) and 602.2 is a unit conversion factor. The parameter *h* has also been referred to as the “confinement length,”^21^ defining a thin volume above the membrane that concentrates the binding sites and directly produces the relationship between $k_{on}^{\left(h\right)}$ and $k_{on}^{\left(vol\right)}$.

While mass action kinetics are generally applicable for both encounter-limited and reaction-limited kinetics in 3D solution (but see Keizer^14^ and Collins and Kimball^15^), it has long been appreciated that the situation may be more complex for 2D kinetics.^8^^,^^10^^,^^12^^,^^13^^,^^19^^,^^40^ This is demonstrated by the results in Table 1 for situations where the diffusion coefficient of the anchor is slow, but the volumetric on-rate constant is fast (rows 5 and 7). For these cases, different estimates of $k_{on}^{\left(mem\right)}$ are obtained at different initial surface density—clearly incompatible with the reaction-limited kinetics. Indeed, the third panel of Figure 2 shows that the SpringSaLaD kinetic data are only approximately fit by a mass action rate law; and Figure 2D displays a terrible fit. A video of one trajectory (Video S1) nicely illustrates how the initial rate is fast, while the binding sites are within “reach”^27^ but fall off as binding sites are left orphaned outside the reach of the remaining slowly diffusing monomers. Because of this, reaction-limited kinetics apply better to high initial concentrations and to shorter time durations, before depletion zones develop. To address the reliability of our method theoretically, even for cases that are influenced by diffusion, we solved the Smoluchowski model^14^ in 2D using VCell (Supporting Text II) to generate the relations plotted in Supporting Text II Figures S7 and S8 and Figure 4 in the main text for the time dependence of the diffusion-limited reaction coefficient. This allowed us to implement a pipeline for computing a weighted average of the on-rate coefficient, $\bar{k}\left(t_{c}\right)$ over time, for any intrinsic on-rate constant, *k*_0_, membrane diffusion coefficient, initial surface density, and desired level of completion. We used this to show that for 19 idealized scenarios, a good correlation between $\bar{k}\left(t_{c}\right)$ and $k_{on}^{\left(mem\right)}$ was displayed (Figure 3).

Thus, as opposed to reaction-limited kinetics, where $k_{on}^{\left(mem\right)}$ can be considered a fixed constant for a broad range of conditions, when the system is “diffusion influenced,” both the initial surface densities and the desired level of completion will strongly determine $k_{on}^{\left(mem\right)}$ (Table 1, rows 5 versus 7; Figure 5). As the system goes further to completion, with fewer of the remaining reactants within reach of the tethers, the system will transition to fully diffusion limited with rate constants much lower than reaction limited; under these conditions, the system will no longer be described by an invariant $k_{on}^{\left(mem\right)}$. Still, if the modeler can analyze the overall reaction network to assess, at least approximately, what the initial concentrations are and how far to completion the membrane-tethered reaction is likely to go, it may be possible to approximate the levels of reactant and product with a single constant $k_{on}^{\left(mem\right)}$ parameter and a mass action ODE; this is especially true for long times, when diffusion is limiting, because of the very slow decline in *k*_*D*_*(t)* (Figure 4 and Supporting Text II Figure S8). The best metric to assess this is the relative RMSD for the fit, which measures how significantly the SpringSaLaD mean trajectory deviates from the mass action fitted curve.

To explore how other molecular structural features might affect dimerization of the monomers tethered to the membrane, we looked at three additional idealized systems in Table 2. In all these, *h* was 20.5 nm (as opposed to 5.5 nm in Table 1). As expected, the longer confinement length decreased the estimated $k_{on}^{\left(mem\right)}$ by a factor of ∼4. The insertion of steric sites between the anchor and the binding site or allowing for flexibility of the linker region has minor effects on $k_{on}^{\left(mem\right)}$, which is relatively well approximated by $k_{on}^{\left(h\right)}$.

For membrane binding of real biological molecules, the average location of binding sites relative to the membrane surface (i.e., *h*) will generally be not well defined, since it is influenced by the steric effects and the variable flexibility of neighboring protein domains. Also, the two binding sites may be parts of very different structures with different distances from the membrane surface. In situations like this, our approach has the potential to provide good estimates of rate constants that can be applied to larger cell signaling systems. Indeed, there may be direct insights that can be realized just by considering the structural details of the interacting membrane molecules. We have illustrated this in relation to adaptor-mediated protein kinase receptor signaling mechanisms, specifically for the interaction of the GEF SOS with its effector Ras (Figure 6). This system has been the subject of many modeling studies^46^^,^^52^^,^^53^^,^^54^^,^^55^^,^^56^^,^^57^^,^^58^ because of the importance of Ras as a key oncogene.^59^ It has been shown that direct catalysis by the SOS catalytic domain of Ras conversion from the GDP to the GTP states is relatively slow. However, binding of Ras to SOS at a site that is not catalytic (termed the “allosteric” site on SOS) significantly accelerates the catalytic activity,^49^^,^^50^ where the catalysis becomes processive.^30^^,^^44^^,^^46^ Here, we confine ourselves to the membrane recruitment of SOS mediated by active EGFR and ask how the specific details of membrane association might affect SOS activity. The fit of the SpringSaLaD simulations to a mass action rate law is excellent in both scenarios of Figure 6, indicating that this system is reaction limited, and mass action is applicable to a range of conditions. The results in Figure 6 suggest that at least part of the mechanism for allosteric activation is due to the close proximity of the SOS catalytic site to the membrane once it is bound to Ras at its allosteric site. Even though SOS is already localized to the membrane by initially binding to EGFR via Grb2 in our computational experiment, pre-binding of the SOS allosteric site to Ras brings it even closer to the membrane. Of course, there could be additional effects such as a direct allosteric enhancement through a conformational change or release of self-inhibition,^30^^,^^44^^,^^46^ but here we focus on the significance of constricting the binding zone through membrane tethers of varying length and flexibility.

To illustrate the application of our approach to cell signaling, we constructed a simple model of EGFR signaling to Ras via SOS in HeLa cells (Figure 7). The results show that the proximity effect established in Figure 6 for the binding of SOS to the second Ras molecule results in the dominance of doubly bound Ras species. This no doubt contributes to the efficiency of the GEF activity associated with SOS. Our simple model also demonstrates the effectiveness of active EGFR in boosting the recruitment of Grb_SOS to the membrane where it presents an effectively high local concentration to Ras. The model, which is available in the VCell database, can be further explored for the effect of varying Ras or SOS concentrations or the behavior of different cell types with different levels of the key signaling models; it could also be used as a starting point for more elaborate models that incorporate more detailed mechanisms and additional downstream signaling events.

In conclusion, we showed how to estimate a 2D rate constant for a mass action rate equation for membrane-tethered molecules, even when diffusion is limiting. While there are many approximations associated with our approach, we believe it represents a significant improvement over the typical simplifications that are used to parameterize large reaction network models. In ODE models, diffusion is assumed to be fast on the timescale of reaction rates; however, our approach is necessary to capture the special local membrane effects that allow conversion of a bulk measurement of the on-rate constant to $k_{on}^{\left(mem\right)}$. In cell-scale PDE (or spatial stochastic) models, where diffusion is considered explicitly, the membrane molecules are not resolved, and the distances are very large on the scale of molecular dimensions. Using $k_{on}^{\left(mem\right)}$ will allow these models to capture the role of molecular structural effects and the influence of membrane diffusion.

## Data availability

All the data used to produce the results in Tables 1 and 2 and in Figures 2, 3, 4, 5, and 6 are available as Excel spreadsheets in the Supporting Data and Information deposited with this paper. The SpringSaLaD and VCell software used to build the models can be downloaded at vcell.org. The results displayed in Figure 7 are from a public VCell model “SOS_Recruitment_Ras_Binding,” which is available on servers accessible through the VCell software.

## Acknowledgments

This work was supported by 10.13039/100000002NIH grants R24 GM137787 and R01 GM132859. We are pleased to acknowledge the advice of Aniruddha Chattaraj with some of the data analysis.

## Author contributions

K.J.P. created the computational models, performed the simulations, analyzed the data and wrote the paper; B.M.S. performed the calculations in Supporting Text I and II and wrote the paper; L.M.L. conceived the research, analyzed the data, and wrote the paper.

## Declaration of interests

The authors declare no competing interests.

## Supplemental information

Document S1. Supporting Text: “Derivation of an equivalent 2D dimerization rate constant for tethered binders in the limit of Dmem ≪ Dvol” (Supporting Text I) and “Applicability of mass action kinetics to dimerization of molecules tethered to the membrane” (Supporting Text II).

Data S1. Spreadsheets describing the calculations in Supporting Text II of Document S1.

Video S1. Video of an example trajectory for the model of Figure 2D

Figure360. An author presentation of Figure 6

Data S2. SpringSaLaD input file for the models in Tables 1 and 2 We include 13 ∗.txt input files for models in Tables 1 and 2. The models for the various cases in Tables 1 and 2 are specified by appropriate choices of molecule numbers, membrane area, diffusion coefficients, and binding rate constants.

Data S3. SpringSaLaD input files for the models of Figure 6 EGFR_Grb_SOS binding to Ras.txt; EGFR_Grb_SOS_prebound at allo binding to Ras.txt.

Data S4. Spreadsheets related to Tables 1 and 2 and Figure 6 Containing the SpringSaLaD simulation results and the fits to determine $k_{on}^{\left(mem\right)}$**.**

Document S2. Article plus supplemental information

## References

[1] Slepchenko B.M., Schaff J.C., Loew L.M. (2003). Quantitative cell biology with the Virtual Cell. Trends Cell Biol..

[2] Eungdamrong N.J., Iyengar R. (2004). Modeling Cell Signaling Networks. Biol. Cell.

[3] Myers P.J., Lee S.H., Lazzara M.J. (2021). Mechanistic and data-driven models of cell signaling: tools for fundamental discovery and rational design of therapy. Curr. Opin. Syst. Biol..

[4] Banga J.R., Villaverde A.F. (2025). Mechanistic dynamic modelling of biological systems: The road ahead. Curr. Opin. Syst. Biol..

[5] Shinobu A., Nagasato-Ichikawa A., Okada M. (2026). Network structures and parameters in multiscale modeling in ErbB signaling networks. Curr. Opin. Cell Biol..

[6] Groves J.T., Dustin M.L. (2003). Supported planar bilayers in studies on immune cell adhesion and communication. J. Immunol. Methods.

[7] Gavutis M., Lata S., Piehler J. (2006). Probing 2-dimensional protein–protein interactions on model membranes. Nat. Protoc..

[8] Adam G., Delbrück M., Rich A., Davidson N. (1968). Structural Chemistry and Molecular Biology.

[9] Pólya G. (1921). Über eine Aufgabe der Wahrscheinlichkeitsrechnung betreffend die Irrfahrt im Straßennetz. Math. Ann..

[10] Torney D.C., McConnell H.M., Porter G.R. (1983). Diffusion-limited reaction rate theory for two-dimensional systems. Proceedings of the Royal Society of London. A. Mathematical and Physical Sciences.

[11] Koudriavtsev A.B., Jameson R.F., Linert W., SpringerLink (2001).

[12] Yogurtcu O.N., Johnson M.E. (2015). Theory of bi-molecular association dynamics in 2D for accurate model and experimental parameterization of binding rates. J. Chem. Phys..

[13] Axelrod D., Wang M.D. (1994). Reduction-of-dimensionality kinetics at reaction-limited cell surface receptors. Biophys. J..

[14] Keizer J. (1987). Diffusion effects on rapid bimolecular chemical reactions. Chem. Rev..

[15] Collins F.C., Kimball G.E. (1949). Diffusion-controlled reaction rates. J. Colloid Sci..

[16] Szabo A. (1989). Theory of diffusion-influenced fluorescence quenching. J. Phys. Chem..

[17] Haugh J.M. (2002). A Unified Model for Signal Transduction Reactions in Cellular Membranes. Biophys. J..

[18] Windisch B., Bray D., Duke T. (2006). Balls and Chains—A Mesoscopic Approach to Tethered Protein Domains. Biophys. J..

[19] Bell G.I. (1978). Models for the Specific Adhesion of Cells to Cells. Science.

[20] Bell G.I., Dembo M., Bongrand P. (1984). Cell adhesion. Competition between nonspecific repulsion and specific bonding. Biophys. J..

[21] Wu Y., Vendome J., Honig B. (2011). Transforming binding affinities from three dimensions to two with application to cadherin clustering. Nature.

[22] Weikl T.R., Hu J., Lipowsky R. (2016). Binding equilibrium and kinetics of membrane-anchored receptors and ligands in cell adhesion: Insights from computational model systems and theory. Cell Adh. Migr..

[23] Xu G.K., Hu J., Weikl T.R. (2015). Binding constants of membrane-anchored receptors and ligands: A general theory corroborated by Monte Carlo simulations. J. Chem. Phys..

[24] Jhaveri A., Chhibber S., Johnson M.E. (2025). Binding affinities for 2D protein dimerization benefit from enthalpic stabilization. bioRxiv.

[25] Xie Z.-R., Chen J., Wu Y. (2014). Linking 3D and 2D binding kinetics of membrane proteins by multiscale simulations. Protein Sci..

[26] Hu J., Xu G.-K., Weikl T.R. (2015). Binding kinetics of membrane-anchored receptors and ligands: Molecular dynamics simulations and theory. J. Chem. Phys..

[27] Zhang Y., Clemens L., Isaacson S.A. (2019). The Influence of Molecular Reach and Diffusivity on the Efficacy of Membrane-Confined Reactions. Biophys. J..

[28] Michalski P.J., Loew L.M. (2016). SpringSaLaD: A Spatial, Particle-Based Biochemical Simulation Platform with Excluded Volume. Biophys. J..

[29] Chattaraj A., Youngstrom M., Loew L.M. (2018). The interplay of structural and cellular biophysics controls clustering of multivalent molecules. bioRxiv.

[30] Iversen L., Tu H.-L., Groves J.T. (2014). Ras activation by SOS: Allosteric regulation by altered fluctuation dynamics. Science.

[31] Abramson J., Adler J., Jumper J.M. (2024). Accurate structure prediction of biomolecular interactions with AlphaFold 3. Nature.

[32] Jumper J., Evans R., Hassabis D. (2021). Highly accurate protein structure prediction with AlphaFold. Nature.

[33] Masison J., Michalski P.J., Schuyler A.D. (2018). mol2sphere: spherical decomposition of multi-domain molecules for visualization and coarse grained spatial modeling. Bioinformatics.

[34] Kamioka Y., Yasuda S., Matsuda M. (2010). Multiple decisive phosphorylation sites for the negative feedback regulation of SOS1 via ERK. J. Biol. Chem..

[35] Fujioka A., Terai K., Matsuda M. (2006). Dynamics of the Ras/ERK MAPK Cascade as Monitored by Fluorescent Probes. J. Biol. Chem..

[36] Hoops S., Sahle S., Kummer U. (2006). COPASI: a COmplex PAthway SImulator. Bioinformatics.

[37] Schaff J., Fink C.C., Loew L.M. (1997). A general computational framework for modeling cellular structure and function. Biophys. J..

[38] Cowan A.E., Moraru I.I., Loew L.M. (2012). Spatial modeling of cell signaling networks. Methods Cell Biol..

[39] Kopelman R. (1986). Rate processes on fractals: Theory, simulations, and experiments. J. Stat. Phys..

[40] Berry H. (2002). Monte Carlo Simulations of Enzyme Reactions in Two Dimensions: Fractal Kinetics and Spatial Segregation. Biophys. J..

[41] Ovchinnikov A.A., Zeldovich Y.B. (1978). Role of density fluctuations in bimolecular reaction kinetics. Chem. Phys..

[42] Toussaint D., Wilczek F. (1983). Particle–antiparticle annihilation in diffusive motion. J. Chem. Phys..

[43] Tachiya M. (1983). Theory of diffusion-controlled reactions: Formulation of the bulk reaction rate in terms of the pair probability. Radiat. Phys. Chem..

[44] Bandaru P., Kondo Y., Kuriyan J. (2019). The Interdependent Activation of Son-of-Sevenless and Ras. Cold Spring Harb. Perspect. Med..

[45] Jorissen R.N., Walker F., Burgess A.W. (2003). Epidermal growth factor receptor: mechanisms of activation and signalling. Exp. Cell Res..

[46] Ren H., Lee A.A., Groves J.T. (2024). Positive feedback in Ras activation by full-length SOS arises from autoinhibition release mechanism. Biophys. J..

[47] Giubellino A., Burke T.R., Bottaro D.P. (2008). Grb2 signaling in cell motility and cancer. Expert Opin. Ther. Targets.

[48] Chook Y.M., Gish G.D., Pawson T. (1996). The Grb2-mSos1 complex binds phosphopeptides with higher affinity than Grb2. J. Biol. Chem..

[49] Margarit S.M., Sondermann H., Kuriyan J. (2003). Structural Evidence for Feedback Activation by Ras·GTP of the Ras-Specific Nucleotide Exchange Factor SOS. Cell.

[50] Gureasko J., Galush W.J., Kuriyan J. (2008). Membrane-dependent signal integration by the Ras activator Son of sevenless. Nat. Struct. Mol. Biol..

[51] McCloskey M.A., Poo M.M. (1986). Rates of Membrane-associated Reactions: Reduction of Dimentionality Revisited. J. Cell Biol..

[52] Eungdamrong N.J., Iyengar R. (2007). Compartment-specific feedback loop and regulated trafficking can result in sustained activation of Ras at the Golgi. Biophys. J..

[53] Tian T., Harding A., Hancock J.F. (2007). Plasma membrane nanoswitches generate high-fidelity Ras signal transduction. Nat. Cell Biol..

[54] Das J., Ho M., Roose J.P. (2009). Digital Signaling and Hysteresis Characterize Ras Activation in Lymphoid Cells. Cell.

[55] Coyle S.M., Lim W.A. (2016). Mapping the functional versatility and fragility of Ras GTPase signaling circuits through in vitro network reconstitution. eLife.

[56] Erickson K.E., Rukhlenko O.S., Kholodenko B.N. (2019). New insights into RAS biology reinvigorate interest in mathematical modeling of RAS signaling. Semin. Cancer Biol..

[57] Huang W.Y.C., Alvarez S., Groves J.T. (2019). A molecular assembly phase transition and kinetic proofreading modulate Ras activation by SOS. Science.

[58] Huang W.Y.C., Alvarez S., Groves J.T. (2021). Relating cellular signaling timescales to single-molecule kinetics: A first-passage time analysis of Ras activation by SOS. Proc. Natl. Acad. Sci. USA.

[59] Pylayeva-Gupta Y., Grabocka E., Bar-Sagi D. (2011). RAS oncogenes: weaving a tumorigenic web. Nat. Rev. Cancer.

