# Supplementary material for: Mesoscale simulations of membrane-tethered reactions to parameterize cell-scale models of signaling
Source: Biophys J. 2026 Apr 25;125(11):2632–47. doi: 10.1016/j.bpj.2026.04.015 (PMC13351843; doi:10.1016/j.bpj.2026.04.015)
Supplement: Document S1. Supporting Text: “Derivation of an equivalent 2D dimerization rate constant for tethered binders in the limit of Dmem ≪ Dvol” (Supporting Text I) and “Applicability of mass action kinetics to dimerization of molecules tethered to the membrane” (Supporting Text II). [file mmc1.pdf]

Biophysical Journal, Volume *125*

Supplemental information

Mesoscale simulations of membrane-tethered reactions to parameterize cell-scale models of signaling

Kelvin J. Peterson, Boris M. Slepchenko, and Leslie M. Loew

Supporting Text

I. Derivation of an equivalent 2D dimerization rate constant for tethered binders in the limit of $D_{mem} \ll D_{vol}$.

Here we derive $k_{on}^{(mem)}$ analytically for conditions pertaining to the first row in Table 1 of the main text. Our approach is conceptually similar to the one previously used in modeling the binding of bivalent haptens to an antibody (Dembo and Goldstein, 1978).

As in SpringSaLad (Michalski and Loew, 2016), we model a binder as a ball of diameter d. A link of length L tethers the ball's center to a membrane-bound anchor. Because the anchor diffuses in the membrane much slower than the binder in the cytosol, the link restricts movements of the binder, so that at any given time, the binder is generally confined to a hemisphere, centered at a current position of the anchor, with the radius $R = L + \frac{d}{2}$ (in the main text, this parameter is termed h). The simulation results, which we seek to understand, were obtained for $d < \frac{1}{5}L$, so that the excluded volume effects due to the binder's finite size can be ignored. We disregard possible effects on $k_{on}^{(mem)}$ of the collisions or entanglement of the tethers, given that the links in SpringSaLad are not affected by each other.

Importantly, we assume that during dimerization, the monomers remain uniformly distributed. A formal analysis of the conditions underlying this assumption is provided in part II below.

With the assumptions outlined above, the effective 2D rate constant $k_{on}^{(mem)}$ for the dimerization of monomers tethered to the membrane can be obtained analytically in terms of the dimerization rate constant $k_{on}^{(vol)}$ of free binders.

Two binders can collide if the distance between their anchors does not exceed $2R$. As mentioned earlier, the anchors can be viewed as "well-mixed" with some surface density σ. Without loss of generality, we may assume that the anchor of one of the monomers is immobile (in what follows, we will call such a monomer 'a given monomer'). The number of binding partners of this monomer is then

$$n = 4\pi R^2 \sigma. \qquad \text{Eq (S1)}$$

Our goal is to derive the rate of dimerization of a given monomer.

We first consider cases where the links between a binder and its anchor are stiff. In such cases, given that $D_{mem} \ll D_{vol}$, the binder may be thought of as uniformly distributed at any time within a hemispherical shell centered at a current position of its anchor. The shell has the outer radius R, the inner radius $r = L - \frac{d}{2}$, and the volume $v_{shell} = \frac{2\pi R^3}{3}\left(1 - \left(\frac{r}{R}\right)^3\right)$, so the binder 'concentration' (more precisely, the binder probability density function) is $\frac{1}{v_{shell}} = [\frac{2\pi R^3}{3}\left(1 - \left(\frac{r}{R}\right)^3\right)]^{-1}$.

The dimerization of a given monomer with a binding partner requires that their binders collide, which occurs at the intersection of their shells (the shared space in Figure S1). The collision probability for the binder of the given monomer, distributed within the shell shaded in Figure S1,

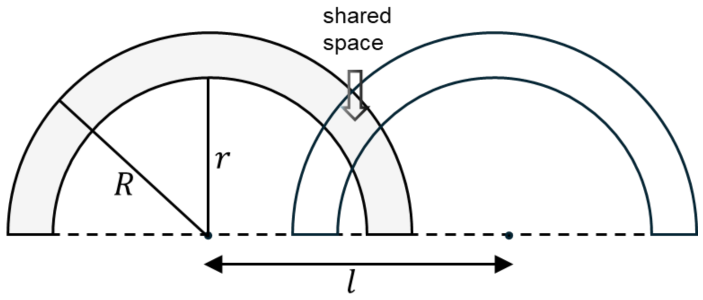

Figure S1. Cross-section of overlapping hemispherical shells.
The shells cover binder positions of two monomers with anchors separated by distance l.

$p_{coll} = \frac{v_{shared}}{v_{shell}}$, where v_{shared} is the volume of the shared space. As v_{shared} depends on distance l separating the anchors, so does the collision probability. Because the anchors are well-mixed, the mobile anchor of the binding partner (the center of the unshaded shell in Figure S1) is uniformly distributed within the circle of radius $2R$ with the center at the fixed anchor. The average collision probability is found by integrating of $p_{coll}(l) = \frac{v_{shared}(l)}{v_{shell}}$ over this circle:

$$\bar{p}_{coll} = \frac{1}{\pi(2R)^2}\int_0^{2\pi} d\varphi \int_0^{2R} p_{coll}(l) l dl = \frac{2}{(2R)^2}\int_0^{2R} p_{coll}(l) l dl. \qquad \text{Eq (S2)}$$

Then the rate of dimerization of the given monomer with a single binding partner is the product of the rate constant of dimerization of free binders $k_{on}^{(vol)}$ and the 'concentration' of the single binder $\frac{1}{v_{shell}}$ modified by the average collision probability: $\bar{p}_{coll}\frac{k_{on}^{(vol)}}{v_{shell}}$.

We now recall that the given monomer has n binding partners defined by Eq (S1). For anchor densities that are not too high, so that the events of three-binder collisions can be ignored, the rate of dimerization of a given monomer with any of the available binding partners is simply $n\bar{p}_{coll}\frac{k_{on}^{(vol)}}{v_{shell}} = \left(\frac{4\pi R^2 \bar{p}_{coll} k_{on}^{(vol)}}{v_{shell}}\right)\sigma$, where the expression in the parenthesis is the sought formula for the equivalent $k_{on}^{(mem)}$:

$$k_{on}^{(mem)} = \frac{4\pi R^2 \bar{p}_{coll} k_{on}^{(vol)}}{v_{shell}} = \frac{4\pi R^2}{v_{shell}^2} k_{on}^{(vol)} \int_0^1 v_{shared}(\rho) 2\rho d\rho, \qquad \text{Eq (S3)}$$

where we introduced the dimensionless variable $\rho = \frac{l}{2R}$.

We now turn to calculations of $v_{shared}(\rho)$. Introducing for brevity the notation $a = \frac{r}{R}$, we observe that for any $a, \rho \leq 1$, the space shared by two intersecting spherical shells is a combination of spherical caps. It is therefore convenient to introduce a function,

$$f(x) = 1 - \frac{3}{2}x + \frac{1}{2}x^3, \qquad \text{Eq (S4)}$$

which for $x = \cos\theta$ yields the volume fraction of a hemisphere that is occupied by a spherical cap with polar angle θ (Figure S2).

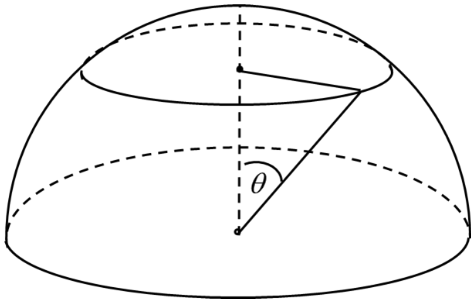

Figure S2. A spherical cap defined by a polar angle θ .
The cap is shown as a part of the corresponding hemisphere.

The structure of $v_{shared}(\rho)$ depends on the value of a. For $a \geq \frac{1}{3}$, the derivation based on stereometric considerations yields the following dependence:

$$\frac{v_{shared}(\rho)}{(2\pi R^3/3)} = \begin{cases} f(\rho) - a^3 f\left(-\frac{\rho}{a}\right), & \text{for } \rho \in \left[0,\ \frac{1}{2}(1-a)\right] \\ f(\rho) + a^3 f\left(\frac{\rho}{a}\right) - f\left(\frac{1-a^2}{4\rho} + \rho\right) - a^3 f\left(\frac{a^2-1}{4\rho a} + \frac{\rho}{a}\right), & \text{for } \rho \in \left[\frac{1}{2}(1-a),\ a\right] \\ f(\rho) - f\left(\frac{1-a^2}{4\rho} + \rho\right) - a^3 f\left(\frac{a^2-1}{4\rho a} + \frac{\rho}{a}\right), & \text{for } \rho \in \left[a, \frac{1}{2}(1+a)\right] \\ f(\rho), & \text{for } \rho \in \left[\frac{1}{2}(1+a), 1\right] \end{cases} \quad \text{. Eq (S5)}$$

Eq (S5) applies to the stiff-tether cases discussed in the main text. Indeed, for $L = 5$ nm and $d = 1$ nm,the outer and inner shell radii are $R = 5.5$ nm, $r = 4.5$ nm, resulting in $a = \frac{r}{R} = \frac{9}{11} > \frac{1}{3}$, and the monomers with larger L and same d are characterized by even larger a. Figure S3 illustrates $\frac{v_{shared}}{\frac{2\pi R^3}{3}}$ as a function of ρ for $a = \frac{9}{11}$.

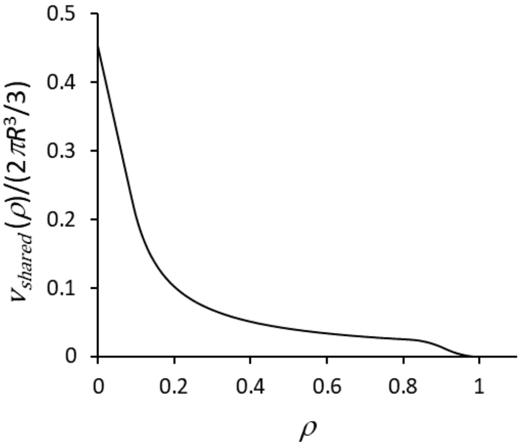

Figure S3. Volume of intersection of two identical shells as a function of distance between their centers.
The volume of the shared space, normalized to the volume of the hemisphere, is shown as a function of the normalized distance $\rho = \frac{l}{2R}$; $L = 5$ nm, $d = 1$ nm.

The integral in Eq(S3) is readily evaluated,

$$\int_0^1 v_{shared}(\rho)2\rho d\rho = \frac{2\pi R^3}{3}\{\frac{1}{5}(1+a^5)+\frac{a}{8}(1-a^2)^2-\frac{1}{80}((1+a)^5-(1-a)^5)\}. \qquad \text{Eq (S6)}$$

Substituting Eq (S6) into Eq (S3) and noting that $v_{shell}=\frac{2\pi R^3}{3}\left(1-\left(\frac{r}{R}\right)^3\right)=\frac{2\pi R^3}{3}(1-a^3)$, we find

$$k_{on}^{(mem)} = \phi(a)\frac{k_{on}^{(vol)}}{R}, \qquad \text{Eq (S7a)}$$

where

$$\phi(a) = \frac{\frac{6}{5}(1+a^5)+\frac{3a}{4}(1-a^2)^2-\frac{3}{40}((1+a)^5-(1-a)^5)}{(1-a^3)^2}. \qquad \text{Eq (S7b)}$$

In the terms of the main text, $\frac{k_{on}^{(vol)}}{R}=\frac{k_{on}^{(vol)}}{h}=k_{on}^{(h)}$, and from Eqs (S7a), $\phi(a)=\frac{k_{on}^{(mem)}}{k_{on}^{(h)}}$. For stiff tethers with length $L = 5$ nm and a binder's diameter $d = 1$ nm, $a=\frac{9}{11}$ and $\frac{k_{on}^{(mem)}}{k_{on}^{(h)}}=1.06$. This is close to $\frac{k_{on}^{(mem)}}{k_{on}^{(h)}}=0.967$ from the first row of Table 1 (main text). Thus, the analytical solution validates the simulation results obtained with SpringSaLaD.

Note that the ratio $\frac{k_{on}^{(mem)}}{k_{on}^{(h)}}$ are not particularly sensitive to tether lengths L in the limit $D_{mem} \ll D_{vol}$ (Figure S4A), and it is nearly the same as in the case of $D_{mem} = D_{vol}$ (second row of Table 1). This appears counterintuitive, given that for $D_{mem} \ll D_{vol}$, the tethering causes the collision probability to decrease with L, whereas for equal diffusivities, the binders are effectively unconstrained by their anchors in their movements within the layer of height $L+\frac{d}{2}$, adjacent to the membrane.

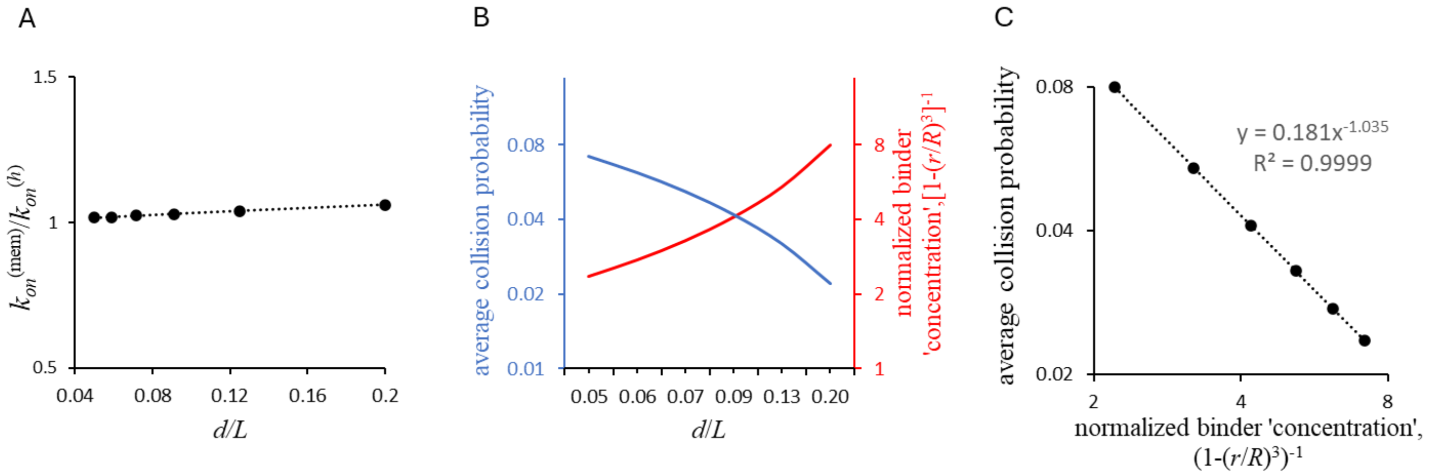

Figure S4. Ratio $k_{on}^{(mem)}/k_{on}^{(h)}$ has low sensitivity to tether length L.
(A) The ratio $k_{on}^{(mem)}/k_{on}^{(h)}$ as a function of the ratio of binder diameter d and tether length L; $d = 1$ nm. (B) Interplay of main determinants of $k_{on}^{(mem)}/k_{on}^{(h)}$ as functions of d/L. (C) The log-log plot of collision probability as a function of binder 'concentration' in the shell, indicating that they are nearly reciprocal; this explains the low sensitivity of $k_{on}^{(mem)}/k_{on}^{(h)}$ to L.

The low sensitivity of the coefficient of proportionality between $k_{on}^{(mem)}$ and $k_{on}^{(h)}$ to L in the limit $D_{mem} \ll D_{vol}$ is explained by the interplay of the collision probability and the normalized binder 'concentration' within the shell, another determinant of $\frac{k_{on}^{(mem)}}{k_{on}^{(h)}}$ (Figure S4B). Indeed, these factors vary with $\frac{d}{L}$ in a nearly reciprocal manner (Figure S4C).

That the value of $\frac{k_{on}^{(mem)}}{k_{on}^{(h)}}$ in the limit $D_{mem} \ll D_{vol}$ is close to that in the case of equal diffusivities is largely due to the assumption of well-mixed anchors: the averaging of $p_{coll}(l) = \frac{v_{shared}(l)}{v_{shell}}$ is done over the uniform anchor distribution (Eq (S3)), and the number of binding partners (Eq (S1)) assumes that the anchors are well-mixed.

We now turn to cases of flexible tethers, again assuming the diameter of a binder d to be significantly less than the tether length L. If a tether is totally flexible, then the entire hemisphere, centered at the anchor, is accessible to the binder, so we have a case of $r = 0$ and, consequently, $a = 0$. For completeness, we will consider a 'semiflexible' tether model, where a portion of the tether adjacent to the anchor is stiff, whereas the remaining segment is flexible, so that the binder again is found with equal probability in a hemispherical shell with the inner radius r, though in this case $(R - r) \neq d$.

We recall that Eq (S5) holds only for $a \geq \frac{1}{3}$. For $a < \frac{1}{3}$, the stereometry yields the following equation for $v_{shared}/(\frac{2\pi R^3}{3})$,

$$\frac{v_{\,shared}(\rho)}{(2\pi R^3/3)} = \begin{cases} f(\rho) - a^3 f\left(-\frac{\rho}{a}\right), & \text{for } \rho \in [0,\ a] \\ f(\rho) - 2a^3, & \text{for } \rho \in \left[a,\ \frac{1}{2}(1-a)\right] \\ f(\rho) - f\left(\frac{1-a^2}{4\rho} + \rho\right) - a^3 f\left(\frac{a^2-1}{4\rho a} + \frac{\rho}{a}\right), & \text{for } \rho \in \left[\frac{1}{2}(1-a),\ \frac{1}{2}(1+a)\right] \\ f(\rho), & \text{for } \rho \in \left[\frac{1}{2}(1+a), 1\right] \end{cases} . \quad \text{Eq (S8)}$$

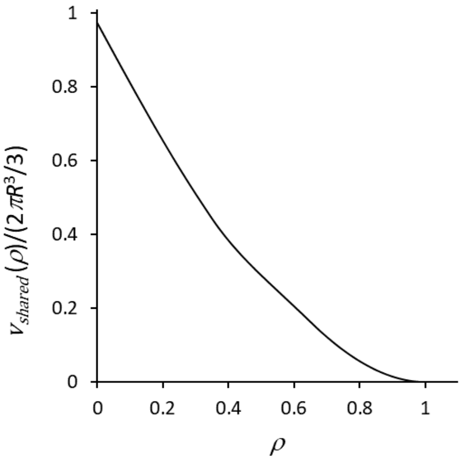

Figure S5. Volume shared by two identical shells as a function of distance between their centers.
The volume of the shared space, normalized by volume of the hemisphere, is shown as a function of $\rho = \frac{l}{2R}$; $\frac{r}{R} =$

Figure S5 illustrates $v_{shared}/(\frac{2\pi R^3}{3})$ as a function of ρ for $a = 0.3$.

Interestingly, while Eq (S8) differs from Eq (S5), its integral is the same as in Eq (S6), so Eqs (S7) hold for all $a \in [0, 1]$. Thus, for the case of 'semiflexible' tethers, the ratio $k_{on}^{(mem)}/(\frac{k_{on}^{(vol)}}{R})$ as a function of $a = \frac{r}{R}$ is described by Eq (S7b); the graph of the ratio as a function of a flexible fraction of the tether is shown in Figure S6.

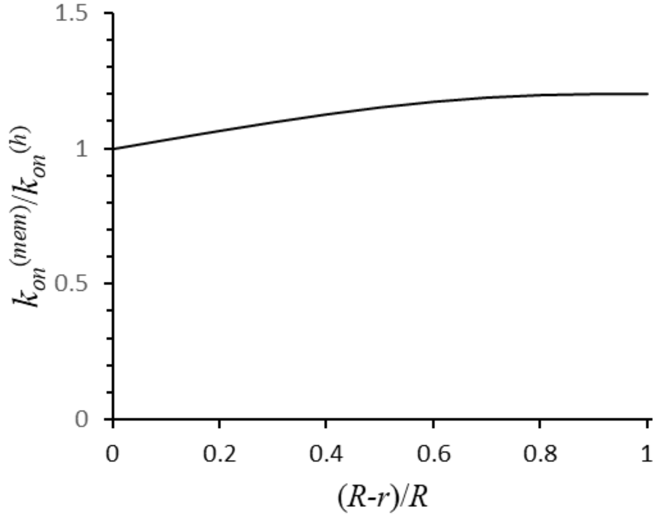

Figure S6. Ratio $k_{on}^{(mem)}/k_{on}^{(h)}$ for the case of monomers with (semi)flexible tethers. The ratio is shown as a function of a flexible fraction of the tether.

For the fully flexible tether, it follows from Eq (S7b) that $\phi(0) = 1.2$, therefore $k_{on}^{(mem)} = \phi(0)\frac{k_{on}^{(vol)}}{R} = 1.2k_{on}^{(h)}$.

II. Applicability of mass-action kinetics to dimerization of molecules tethered to the membrane.

Determining the equivalent two-dimensional (2D) rate constant $k_{on}^{(mem)}$ for dimerization of monomers tethered to the membrane is based on the assumption that its kinetics can be accurately approximated as $k\sigma_A\sigma_B$, where $\sigma_A(t)$ and $\sigma_B(t)$ are the 2D densities of the binding partners A and B, and k is the rate constant, i.e. it is independent of time. In the case of homodimerization, the corresponding rate equation, $\partial_t\sigma = -2k\sigma^2$, has the exact solution,

$$\sigma(t) = \frac{\sigma_0}{1+2k\sigma_0 t}, \qquad \text{Eq (S9)}$$

where σ_0 is the initial monomer concentration, $\sigma_0 = \sigma(0)$. In part I of this text, we showed that if the binding partners remain distributed uniformly during the entire process, the dimerization is indeed described by mass-action kinetics and used this assumption to derive $k_{on}^{(mem)}$. If the mechanism of monomer mixing is the diffusion of their anchors, it is qualitatively clear that the

mass-action rate law would apply assuming high anchor diffusivity and slow association of the binding sites upon collision. Under these conditions, the mass-action rate constant essentially coincides with the rate constant of the intrinsic binding. However, this assumption is not always true, as some examples of the main text indicate. In this section, we use the idealized cases presented in Table 1 and the first row of Table 2 of the main text to provide insight into factors affecting applicability of mass-action kinetics and further validate our simulation results.

Binding of molecules embedded in the membrane ($L \to 0$).
It is instructive to first analyze the system in the limit of $L \to 0$ and then generalize the results to nonzero L. The criteria of applicability of mass-action kinetics in this limit can be studied by applying the Smoluchowski theory, which was extensively reviewed in the past, see, e.g., (Keizer, 1987); for more recent overview, see (Yogurtcu and Johnson, 2015) and references therein. The theory assumes that the association, or intrinsic binding, of the molecules occurs on collision with some rate constant k_0, whereas the observed rate coefficient, which is also influenced by molecular diffusion, could be time-dependent and therefore may not be accurately described by mass-action kinetics. However, for the reaction-limited binding characterized by high diffusivity of binding partners and slow initrinsic binding, the effects of diffusion are negligible, so that the observable rate coefficient coincides with k_0 and the mass-action approximation applies both in 3D and 2D.

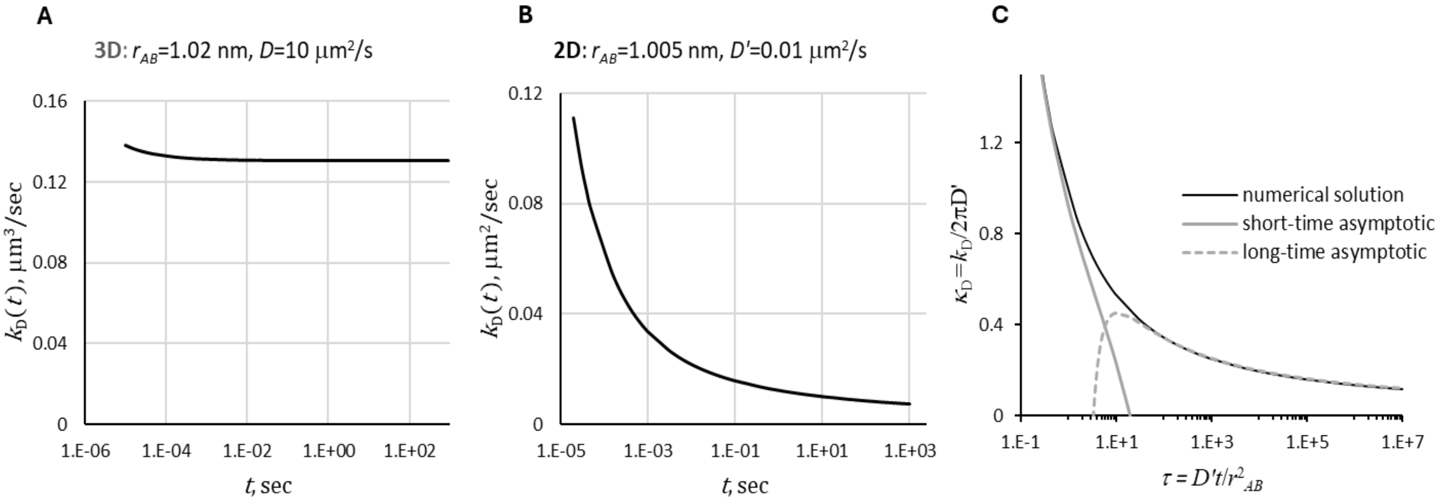

Figure S7. Time dependence of k_D for diffusion-limited binding in 3D and 2D.
Time dependencies $k_D(t)$ in 3D (A) and 2D (B) shown for typical values of r_{AB} and D'.
(C) Numerically obtained time dependence of the 2D binding rate coefficient, shown in dimensionless coordinates $\kappa_D = k_D/(2\pi)$ and $\tau = D't/r_{AB}^2$ (black solid curve), is compared with analytical short-time and long-time asymptotic solutions (Torney, McConnell, 1983; Barzykin and Tachiya, 1993): $\kappa_D = \frac{1}{\sqrt{\pi\tau}} + \frac{1}{2} - \frac{1}{4}\sqrt{\frac{\tau}{\pi}} + \ldots$ (grey solid curve) and $\kappa_D = 2\left(\xi - \gamma\xi^2 - \left(\frac{\pi^2}{6} - \gamma^2\right)\xi^3 + \cdots\right)$, where $\xi = \frac{1}{\ln(4\tau) - 2\gamma}$ and Euler's constant $\gamma = 0.5772157\ldots$. (grey dashed curve).

In contrast, for diffusion-limited reactions characterized by fast initrinsic binding ($k_0 \to \infty$), the 3D kinetics differs significantly from that in 2D. While the theory yields time-dependent rate coefficients for both 2D and 3D, the rate coefficient of diffusion-limited binding in 3D relatively quickly approaches its steady value $k_D = 4\pi r_{AB} D'$, where r_{AB} is the sum of effective radii of the

binding partners, $r_{AB} = r_A + r_B$, and D' is the sum of their diffusion coefficients, $D' = D_A + D_B$ (Figure S7A). Thus, bimolecular binding in 3D is in effect well described by mass-action kinetics both in the association- and diffusion-dominated limits.

In the intermediate regime with finite k_0 and k_D, sometimes termed “diffusion-influenced”, the 3D binding is also well approximated by mass-action kinetics (Collins and Kimball, 1949) with the rate constant

$$k = \frac{k_0 k_D}{k_0 + k_D} . \qquad \text{Eq (S10)}$$

The situation in 2D is significantly different (Figure S7B), as the rate coefficient $k_D(t)$ in this case does not stabilize after the intial steep decrease, but rather continues descending to zero (Torney and NcConnel, 1983). This is a consequence of the loss of dimensionality, which reducies to zero the probability for a molecular pair to escape an encounter, resulting in depletion zones around the binders that have not yet reacted. In Figure S7C, the black solid curve represents the “universal” time dependence of the rate coefficient for the diffusion-limited binding in 2D, with k_D scaled by $2\pi D'$ and t scaled by r_{AB}^2/D', see *Computational details* below.

Table S1. Normalized rate coefficient for diffusion-limited binding in 2D as a function of dimensionless time

$D't/r_{AA}^2$	$k_D/2\pi D'$	$D't/r_{AA}^2$	$k_D/2\pi D'$	$D't/r_{AA}^2$	$k_D/2\pi D'$	$D't/r_{AA}^2$	$k_D/2\pi D'$	$D't/r_{AA}^2$	$k_D/2\pi D'$	$D't/r_{AA}^2$	$k_D/2\pi D'$	$D't/r_{AA}^2$	$k_D/2\pi D'$	$D't/r_{AA}^2$	$k_D/2\pi D'$
0.198015	1.766791	188.1142	0.313627	584.144	0.268346	980.1738	0.25157	39602.98	0.173029	79205.96	0.163375	2079156	0.129257	6039454	0.120976
0.297022	1.49689	198.0149	0.311271	594.0447	0.267768	990.0745	0.251264	40593.05	0.172666	80196.03	0.163212	2178164	0.128867	6138462	0.120858
0.39603	1.344426	207.9156	0.309059	603.9454	0.267202	1980.149	0.233219	41583.13	0.172312	81186.11	0.163051	2277171	0.128501	6237469	0.120742
0.495037	1.241926	217.8164	0.306978	613.8462	0.266647	2970.224	0.222209	42573.2	0.171969	82176.18	0.162892	2376179	0.128154	6336477	0.120628
0.990075	1.007724	227.7171	0.305014	623.7469	0.266103	3960.298	0.21527	43563.28	0.171636	83166.26	0.162734	2475186	0.127822	6435484	0.120517
1.485112	0.879671	237.6179	0.303155	633.6477	0.26557	4950.373	0.210245	44553.35	0.171311	84156.33	0.162579	2574194	0.127505	6534492	0.120407
1.980149	0.806328	247.5186	0.301392	643.5484	0.265047	5940.447	0.206333	45543.43	0.170994	85146.41	0.162427	2673201	0.127201	6633499	0.120298
2.475186	0.75647	257.4194	0.299724	653.4492	0.264534	6930.522	0.203148	46533.5	0.170685	86136.48	0.162278	2772209	0.126909	6732507	0.120192
2.970224	0.719406	267.3201	0.298137	663.3499	0.264031	7920.596	0.20047	47523.58	0.170384	87126.56	0.16213	2871216	0.12663	6831514	0.120087
3.465261	0.690319	277.2209	0.296615	673.2507	0.263537	8910.671	0.198167	48513.65	0.170089	88116.63	0.161984	2970224	0.126361	6930522	0.119985
3.960298	0.666675	287.1216	0.295158	683.1514	0.263052	9900.745	0.196154	49503.73	0.169801	89106.71	0.16184	3069231	0.126101	7029529	0.119883
4.455335	0.646923	297.0224	0.293763	693.0522	0.262575	10890.82	0.194369	50493.8	0.169521	90096.78	0.161696	3168238	0.125851	7128536	0.119784
4.950373	0.630062	306.9231	0.292425	702.9529	0.262107	11880.89	0.192767	51483.87	0.169246	91086.85	0.161555	3267246	0.12561	7227544	0.119685
5.44541	0.615425	316.8238	0.291141	712.8536	0.261647	12870.97	0.191317	52473.95	0.168977	92076.93	0.161417	3366253	0.125377	7326551	0.119589
5.940447	0.602536	326.7246	0.289907	722.7544	0.261194	13861.04	0.189994	53464.02	0.168715	93067	0.16128	3465261	0.125151	7425559	0.119493
6.435484	0.591071	336.6253	0.288719	732.6551	0.26075	14851.12	0.188779	54454.1	0.168458	94057.08	0.161144	3564268	0.124932	7524566	0.1194
6.930522	0.580771	346.5261	0.287574	742.5559	0.260312	15841.19	0.187655	55444.17	0.168206	95047.15	0.16101	3663276	0.12472	7623574	0.119307
7.425559	0.571452	356.4268	0.28647	752.4566	0.259882	16831.27	0.186613	56434.25	0.16796	96037.23	0.160878	3762283	0.124515	7722581	0.119216
7.920596	0.562964	366.3276	0.285405	762.3574	0.259459	17821.34	0.185639	57424.32	0.167718	97027.3	0.160746	3861291	0.124315	7821589	0.119126
8.415633	0.555171	376.2283	0.284374	772.2581	0.259042	18811.42	0.184728	58414.4	0.167481	98017.38	0.160617	3960298	0.124122	7920596	0.119038
8.910671	0.548035	386.1291	0.283378	782.1589	0.258632	19801.49	0.183873	59404.47	0.167249	99007.45	0.160489	4059305	0.123933	8019603	0.11895
9.405708	0.541435	396.0298	0.282412	792.0596	0.258229	20791.56	0.183066	60394.54	0.167021	198014.9	0.152737	4158313	0.12375	8118611	0.118864
9.900745	0.535248	405.9305	0.281477	801.9603	0.257831	21781.64	0.182303	61384.62	0.166798	297022.4	0.147872	4257320	0.123571	8217618	0.118779
19.80149	0.467884	415.8313	0.28057	811.8611	0.25744	22771.71	0.18158	62374.69	0.166579	396029.8	0.144723	4356328	0.123397	8316626	0.118695
29.70224	0.428993	425.732	0.27969	821.7618	0.257054	23761.79	0.180893	63364.77	0.166364	495037.3	0.142402	4455335	0.123227	8415633	0.118613
39.60298	0.405744	435.6328	0.278835	831.6626	0.256674	24751.86	0.180238	64354.84	0.166153	594044.7	0.140573	4554343	0.123062	8514641	0.118531
49.50373	0.38948	445.5335	0.278004	841.5633	0.2563	25741.94	0.179614	65344.92	0.165946	693052.2	0.139067	4653350	0.122901	8613648	0.11845
59.40447	0.377121	455.4343	0.277195	851.4641	0.255931	26732.01	0.179018	66334.99	0.165742	792059.6	0.137791	4752358	0.122743	8712656	0.118371
69.30522	0.367247	465.335	0.276409	861.3648	0.255568	27722.09	0.178447	67325.07	0.165541	891067.1	0.136687	4851365	0.122589	8811663	0.118292
79.20596	0.359101	475.2358	0.275643	871.2656	0.255209	28712.16	0.177899	68315.14	0.165344	990074.5	0.135714	4950373	0.122438	8910671	0.118215
89.10671	0.352197	485.1365	0.274897	881.1663	0.254856	29702.24	0.177373	69305.22	0.165151	1089082	0.134848	5049380	0.122291	9009678	0.118138
99.00745	0.346229	495.0373	0.27417	891.0671	0.254507	30692.31	0.176866	70295.29	0.16496	1188089	0.134066	5148387	0.122147	9108685	0.118062
108.9082	0.340991	504.938	0.27346	900.9678	0.254163	31682.38	0.176379	71285.36	0.164773	1287097	0.133355	5247395	0.122006	9207693	0.117988
118.8089	0.336338	514.8387	0.272768	910.8685	0.253824	32672.46	0.175909	72275.44	0.164589	1386104	0.132704	5346402	0.121868	9306700	0.117914
128.7097	0.332161	524.7395	0.272092	920.7693	0.253489	33662.53	0.175456	73265.51	0.164407	1485112	0.132103	5445410	0.121733	9405708	0.117841
138.6104	0.328384	534.6402	0.271432	930.67	0.253159	34652.61	0.175018	74255.59	0.164228	1584119	0.131546	5544417	0.121601	9504715	0.117769
148.5112	0.324943	544.541	0.270787	940.5708	0.252833	35642.68	0.174595	75245.66	0.164052	1683127	0.131028	5643425	0.121471	9603723	0.117697
158.4119	0.32178	554.4417	0.270157	950.4715	0.252511	36632.76	0.174185	76235.74	0.163879	1782134	0.130542	5742432	0.121344	9702730	0.117627
168.3127	0.318856	564.3425	0.26954	960.3723	0.252194	37622.83	0.173787	77225.81	0.163708	1881142	0.130087	5841440	0.121219	9801738	0.117557
178.2134	0.316148	574.2432	0.268937	970.273	0.25188	38612.91	0.173402	78215.89	0.163541	1980149	0.129663	5940447	0.121096	9900745	0.117488

It was obtained by solving the Smoluchowski model numerically with *Virtual Cell* (VCell), a suite of numerical tools for solving models arising in cell biology (Slepchenko and Loew, 2010; Resasco et al., 2012). The values of $k_D/2\pi D'$ as a function of $D't/r_{AB}^2$ are shown in Table S1.

Computation details

The Smoluchowski model defines the binding rate coefficient $k(t)$ in terms of pair survival probability $u(r,t)$, the probability that an isolated pair of binding partners, initially separated by distance r, does not react by time t. Here and below, we assume uniformity of space, system axial symmetry, and that irreversible binding occurs on encounter. Without loss of generality, one of the binding partners (molecule A) can be fixed at the origin and assigned the effective reaction radius, $r_{AB} = r_A + r_B$, whereas the center of the other binding partner (molecule B) diffuses with the effective diffusion coefficient $D' = D_A + D_B$ in the domain $r \in [r_{AB}, \infty)$. The survival probability u is governed by the equation, $u_t = D'\Delta u$, where Δ is the 2D diffusion operator, $\Delta = \frac{1}{r}\partial_r(r\partial_r)$, see, e.g., (Barzykin and Tachiya, 1993).The governing equaton is solved with the flux boundary condition, $2\pi r_{AB}\partial_r u(r_{AB},t) = k_0 u(r_{AB},t)$, the initial condition $u(r,0) = 1$, and the boundary condition $u(\infty,t) = 1$ at $r_{AB} \to \infty$. The 'observed' reacton rate coefficient $k(t)$ is then defined as $k(t) = 2\pi r_{AB}\partial_r u(r_{AB},t)$. For diffusion-limited reactions, $k_0 \to \infty$, so the boundary condition at $r = r_{AB}$ becomes $u(r_{AB},t) = 0$. Thus, $k_D(t) = 2\pi r_{AB}\partial_r u(r_{AB},t)$ for the reactive boundary condition $u(r_{AB},t) = 0$.

To solve this model with VCell, the governing equation must be rewritten in a 1D 'Cartesian' diffusion-advection form: using the change of variables, $u = U/r$, the equation becomes $U_t = D'\partial_{rr}^2 U - \partial_r(vU)$, with $v = D'/r$. To avoid numerical spatial differentiation, which is lower order of accuracy than numerical integration, we solved the model both outside and inside the reaction boundary. Outside the reactive boundary, i.e., for $r \in [r_{AB}, r_{max}]$, the equation for U was solved using the flux density $f_U(r_{AB},t) = -k_0 U(r_{AB},t)$ with sufficiently large k_0, the initial condition $U(r,0) = r$, and the boundary condition $f_U(r_{max},t) = 0$ at $r = r_{max}$. Inside the reactive boundary, i.e., for $r \in [0, r_{AB})$, we integrate the flux across the reactive boundary over time, $I(t) = \int_0^t k_D(t)dt$. For this, we solve there the 1D diffusion equation, $u_t = D\partial_{rr}^2 u$, with sufficiently high diffusivity D, so that $u(r,t)$ is equal to its average $\bar{u}(t)$ with high precision. The equation was solved with the flux boundary condition, $f_u(r_{AB},t) = f_U(r_{AB},t)/r$ and $f_u(0,t) = 0$, and the intial condition $u(r,0) = 0$. Thus, $\bar{u}(t)r_{AB}$ yields the integrated flux density and the integrated flux $I(t)$ is given by $2\pi r_{AB}^2\bar{u}(t)$. Finally we determine $k_D(t)$ by numerical time differentiation of $\bar{u}(t)$, $k_D(t) = \frac{dI}{dt} = 2\pi r_{AB}^2\bar{u}_t$. The computations were performed using VCell fully-implicit finite-volume solver (Slepchenko et al, 2018) for $r_{AB} = 0.001005$ μm, $r_{max} = 10$ μm, $D' = 0.01$ μm²/s. Results obtained with the increasing k_0 and decreasing space discretization parameter Δr, showed that the relative numerical errors of the solution with $k_0 = 10^6$ μm²/s and $\Delta r = 10^{-5}$ μm were within few percent. Comparison against the short-time and long-time analytical asymptotic solutions (Figure S7C) indicated relative errors in the 2-4% range. We nondimensionalized the solution obtained with $k_0 = 10^6$ μm²/s and $\Delta r = 10^{-5}$ μm to obtained the universal dependence of $k_D/2\pi D'$ on normalized time $\tau = D't/r_{AB}^2$ (Table S1).VCell implementation of the model can be found in the public VCell MathModel database under 'boris'. Model name: Diffusion_limited_binding_2D'; simulations: Simulation3 and its copies.

Since the function $\kappa_D(\tau)$ in Figure S7C approaches zero as $\tau \to \infty$ and diverges to infinity when $\tau \to 0$, the 2D bimolecular binding in the intermediate regime with finite intrinsic rate constants k_0 always begins as reaction-limited and thus initially is well approximated by mass-action kinetics, but eventually crosses over to the diffusion-limited mode with a time-dependent rate coefficient. It is therefore clear that in 2D, the mass-action kinetics given by Eq (S9) with a single-valued k cannot adequately describe the kinetics of diffusion-influenced binding over arbitrary time periods. However, as we explain later, under certain conditions mass-action kinetics can be a reasonably accurate approximation even for diffusion-limited reactions.

The time dependence of k_D in 2D renders the corresponding observed rate coefficient time-dependent as well, due to the general relation between the pair survival probabilities in the diffusion-limited and intermediate regimes (Pedersen, 1980), which holds for the systems of all dimensions (see Appendix in (Tachiya, 1983)). This property, originating from linearity of the Smoluchowski model and the fact that the two regimes differ only by conditions at the reactive boundary and not by the equation itself, allows one to connect the kinetic coefficients of the two regimes (Szabo, 1989),

$$\hat{k}(s) = k_0\hat{k}_D(s)/(k_0 + s\hat{k}_D(s)), \qquad \text{Eq (S11)}$$

where $\hat{k}_D(s) = \int_0^\infty k_D\,(t)e^{-st}dt$ is the Laplace transform of $k_D(t)$, and $\hat{k}(s) = \int_0^\infty k(t)e^{-st}dt$ is the Laplace transform of the observed rate coefficient $k(t)$ in the intermediate regime with a finite k_0; the Laplace variable s has units of the inverse time. Note that Eq (S11) reduces to Eq (S10), in 3D, where both k and k_D are effectively constant on the time scale of interest.

In lower dimensions, the meaning of $s\hat{k}_D(s) = \frac{1}{s^{-1}}\int_0^\infty k_D(t)\exp\,(-\frac{t}{s^{-1}})dt$ is that of a weighted average of $k_D(t)$ over time $t{\sim}s^{-1}$, given that the main contributions to the integral come from the times t that do not significantly exceed s^{-1}; we therefore denote $s\hat{k}_D(s)$ as $\bar{k}_D(s^{-1})$,

$$\bar{k}_D(s^{-1}) = \frac{1}{s^{-1}}\int_0^\infty k_D(t)\exp\,(-\frac{t}{s^{-1}})dt. \qquad \text{Eq (S12)}$$

We define time to completion t_c as the time required for a reaction to reach a desired level of completion p, $p \in (0,1)$; so for irreversible reactions, $\sigma(t_c)/\sigma_0 = 1 - p$ (for reversible reactions, see subsection *Reversible binding of tethered monomers*). While Eq (S11) holds for arbitrary s, we are interested in $s^{-1} = t_c$, so from Eqs (S12),

$$\bar{k}_D(t_c) = \frac{1}{t_c}\int_0^\infty k_D\,(t)\exp\left(-\frac{t}{t_c}\right)dt\ , \qquad \text{Eq (S13)}$$

and from Eq (S11),

$$\bar{k}(t_c) = \frac{1}{t_c}\int_0^\infty k\,(t)\exp\left(-\frac{t}{t_c}\right)dt\ = k_0\bar{k}_D(t_c)/(k_0 + \bar{k}_D(t_c)); \qquad \text{Eq (S14)}$$

(note the structural similarity between Eq (S14) and Eq (S10)).

Yogurtcu and Johnson introduced a time-dependent parameter $\delta(t) = 1 - k(t)/k_0$ as a measure of deviation from reaction-limited kinetics (Yogurtcu and Johnson, 2015). We define a somewhat different measure, $\delta = \bar{k}(t_c)/\bar{k}_D(t_c)$, which for given t_c quantitatively identifies reaction kinetics in the spectrum between the reaction-contolled ($\delta = 0$) and diffusion-limited ($\delta = 1$) regimes. Our analysis yields an exact explicit formula for δ in terms of k_0 and $k_D(t)$,

$$\delta = \frac{\bar{k}(t_c)}{\bar{k}_D(t_c)} = \left(1 + \frac{\bar{k}_D(t_c)}{k_0}\right)^{-1}, \qquad \text{Eq (S15)}$$

which follows from Eqs (S13, S12).

Note that while the condition $\delta \ll 1$, which indicates the association-influnced binding, is sufficient for the mass-action approximation, it is not necessary. As we demonstrate later, the mass-action approximation can be reasonably accurate under certain conditions for $\delta \approx 1$ as well.

Irreversible binding of tethered monomers.
Generalizing the theory to dimerization of tethered molecules depends on dimensionless parameter $\sigma_0 \pi h^2$ determining how many neighboring binders are initially within the reach of a given monomer.

We first consider cases with $\sigma_0 \pi h^2 < 1$ (exemplified by first eight rows in Table 1 of the main text). For these cases, the reactive boundary is a hemisphere with radius h centered at a monomer's anchor.Then the corresponding effective 2D radius, which is the average of tether projections on the membrane, is $r_A^{(2D)} = h/2$; therefore $r_{AA}^{(2D)} = 2r_A^{(2D)} = h$. One can also show that upon projection onto the membrane, the effective membrane diffusivity of the binder is $D_A^{\text{eff}} = D_{mem}/2$, and therefore $D' = 2D_A^{\text{eff}} = D_{mem}$ .Given these parameters, we can determine $k_D(t)$ by utilizing $\kappa_D(\tau)$ from Table S1 (see also the corresponding Excell worksheet in the supplimentary files): $k_D(t) = 2\pi D_{mem} \cdot \kappa_D(D_{mem} t/h^2)$.

Noting that the association rate constant is now defined as $k_{on}^{(h)} = k_{on}^{(vol)}/h$, we can evaluate the integral in Eq (S13) and then use Eqs (S14) and (S15) to compute weighted averages of the irreversible-binding rate coefficient $\bar{k}_{irr}(t_c)$ and the corresponding δ, for any combination of $k_{on}^{(h)}$, h, D_{mem}, and t_c (note that, as we discuss later, t_c is affected by an initial monomer density σ_0, level of reaction completion p, as well as by either $k_{on}^{(h)}$ or D_{mem}, depending on whether the kinetics is association- or diffusion-influenced).

Table S2 below presents values of $\bar{k}_{irr}(t_c)$ obtained with $h = 5.5$ nm for varying parameter sets, in comparison with the corresponding values of $k_{on}^{(mem)}$, as the latter are also averages of $k_{irr}(t)$ yielded by fitting Eq (S9) to results of SpringSaLaD simulations with the same parameter sets, see also Figure 3 in the main text. The times to completion t_c (column 7), corresponding to $p =$

Table S2. $k_{on}^{(mem)}$ from SpringSaLaD simulations vs. $\bar{k}_{irr}(t_c)$ based on Smoluchowski theory for cases with $\sigma_0 \pi h^2 < 1$

row ##	σ_0, μm^{-2}	D_{mem}, μm^2/s	$k_{on}^{(h)}$, μm^2/s	$k_{on}^{(mem)}$, μm^2/s	Relative RMSD	t_c, s	$\bar{k}_{irr}(t_c)$, μm^2/s	δ
1	2500	0.01	0.003	0.0029	0.0055	0.2695	0.0025	0.16
2	2500	1	0.003	0.0031	0.0055	0.2645	0.0030	0.0019
3	25	0.01	0.003	0.0024	0.0053	33.78	0.0023	0.23
4	25	1	0.003	0.0030	0.0048	26.62	0.0030	0.0030
5	2500	0.01	0.3	0.084	0.086	0.0138	0.023	0.97
6	2500	1	0.3	0.26	0.016	0.0033	0.27	0.085
7	25	0.01	0.3	0.018	0.039	5.372	0.011	0.96
8	25	1	0.3	0.20	0.022	0.425	0.25	0.092
9	25	0.01	0.03	0.012	0.02	4.0000	0.0086	0.71
10	25	1	0.03	0.029	0.009	2.8300	0.029	0.024
11	2500	1	0.03	0.029	0.01	0.0264	0.029	0.030
12	2500	0.01	0.03	0.020	0.03	0.0436	0.012	0.59
13	250	0.01	0.003	0.0025	0.008	2.0000	0.0024	0.19
14	250	0.01	0.3	0.031	0.05	0.3130	0.015	0.95
15	250	1	0.003	0.003	0.009	2.5000	0.0030	0.0024
16	250	1	0.3	0.23	0.01	0.0374	0.26	0.13

0.8 in all rows, except for rows 10 ($p = 0.69$), 13 ($p = 0.72$), and 15 ($p = 0.795$), were retrieved from the simulated dimer densities $[dimer]_t$.The accuracy of fitting was characterized by computing the relative root-mean-square deviation (relative RMSD) defined as $(\sum_i([dimer]_{t_i}^{sim} - ([dimer]_{t_i}^{appr}))^2 / \sum_i([dimer]_{t_i}^{sim})^2)^{1/2}$, where the summation was perfomed over all time points $t_i \leq t_c$. The difference in the first sum is between the simulated and approximated by mass-action kinetics dimer densities corresponding to same t_i.

The comparison of the data in columns 5 and 8 (highlighted) indicates similar trends in responses to parameter changes. As expected, more pronounced differences between $k_{on}^{(mem)}$ and $\bar{k}_{irr}(t_c)$ correlate with higher relative RMSD (rows 5, 7, 9, 12, and 14), but even in these cases, the respective values are of same order of magnitude. Overall, the degree of correspondence gives credence to both the simulations and theory.

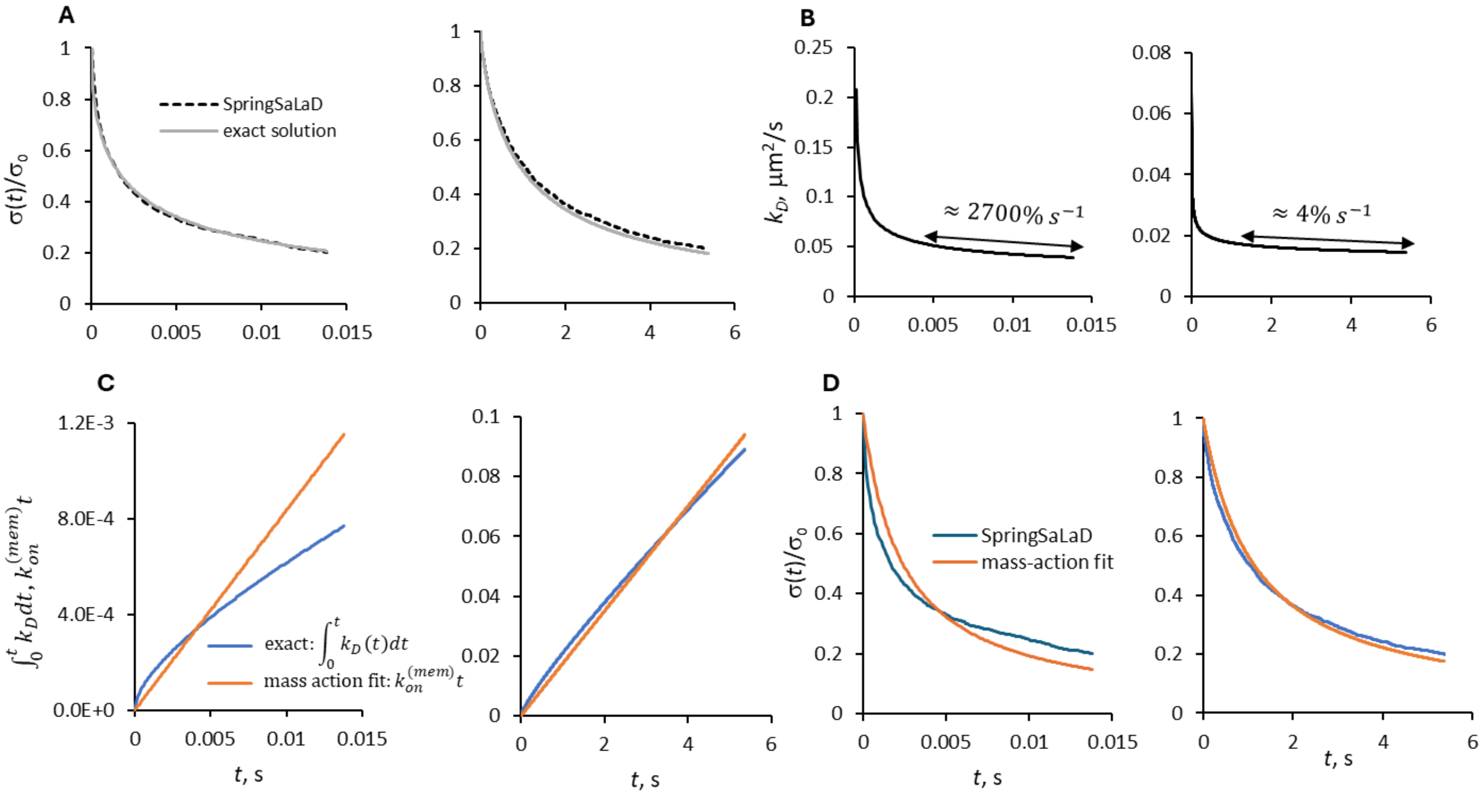

Figure S8. Comparing conditions of rows 5 and 7 of Table 1: Slow diffusion-limited binding is accurately described by mass-action kinetics.

(A) SpringSaLaD solutions (black dashed curves) are consistent with exact diffusion-limited kinetics, $\sigma(t)/\sigma_0 = \left(1 + 2\sigma_0 \int_0^t k_D(t)dt\right)^{-1}$ (grey solid curves), both for the fast reaction with $\sigma_0 = 2500$ μm^{-2} (left panel) and the slow one with $\sigma_0 = 25$ μm^{-2} (right panel). (B) Because of the same $r_{AA}^{(2D)} = h$ and $D' = D_{mem}$ in both rows, the two reactions have the same $k_D(t)$ which is shown for the times to 80% completion of the fast (left) and slow (right) reactions. Average grades of $k_D(t)$ decline during the logarithmic phase, shown for the time intervals [0.005 s, 0.0138 s] (left) and [1 s, 5.372 s] (right), differ by more than two orders of magnitude on the two timescales, with the slow reaction having nearly constant rate coefficient. (C) As the fitting to mass-action kinetics (Eq(S9)) replaces the exact function $\int_0^t k_D(t')dt'$ with the approximate linear function $k_{on}^{(mem)}t$, the two functions are compared using the best-fit values of $k_{on}^{(mem)}$, for the fast (left) and slow (right) reactions. (D) Best mass-action fits of $\sigma(t)/\sigma_0$ for the fast (left) and slow (right) reactions.

Remarkably, the conditions described in rows 7 and 14 are effectively diffusion-limited ($\delta = 0.96$ and 0.95, respectively), yet they are approximated relatively well by mass-action kinetics. To explain this, it is instructive to compare the reaction of row 7 to the one described in row 5. The latter is also diffusion-limited ($\delta = 0.97$) but its mass-action fit is much less accurfate. For both reactions, SpringSaLaD results are indeed close to the diffusion-limited kinetics $\sigma(t)/\sigma_0 = (1 + 2\sigma_0 \int_0^t k_D(t)dt)^{-1}$ (Figure S8A), which in and of itself provides yet another validation of the simulaiton results. The function $k_D(t)$ used in both cases is the same, as it is determined by the same $r_{AA}^{(2D)} = h = 5.5$ nm and $D' = D_{mem} = 0.01$ μm²/s. The only parameter that is different in the conditons of rows 5 and 7 is σ_0, which affects the time to complition t_c. Indeed, it follows from Eq (S9) that $\sigma(t)/\sigma_0 = 1 - p = (1 + 2\sigma_0 k t_c)^{-1}$, yielding $t_c = \frac{1}{2k\sigma_0}\frac{p}{(1-p)}$. Thus, for a given p, higher values of σ_0 result in shorter t_c, and this makes the difference.

The effect of t_c on applicability of mass-action kinetics has to do with biphasic behavior of $k_D(t)$ illustrated by its plots in linear coordinates in Figure S8B: after the initial steep descent, the function nearly plateaus as its decline becomes logarithmically slow. Thus, the slow diffusion-limited reaction operating on a long-time scale (large t_c) is characterized for most of the time by a nearly constant rate coefficient order D_{mem} and, therefore, lends itself to more accurate approximation by mass-action kinetics (Figure S8C-D).

In cases with $\sigma_0 \pi h^2 > 1$, exemplified by row 1 in Table 2 of the main text, geometric constraints imposed by the tethers and slow anchor diffusion are less limiting in two aspects. First, multiple binding sites may now interact under the hemisphere of radius h. As a result, the tether length no longer represents the effective reaction radius, which is now determined by the radii of the binding sites $d/2$. Second, the main mixing mechanism is now volumetric diffusion. Because the area of the hemisphere is twice the area of its projection on the membrane, and both are covered by diffusion within the same time, the corresponding effective 2D diffusivity is $D_{vol}/2$. Thus, to compute $\bar{k}_{irr}(t_c)$ and δ for these cases, one can again use Eqs (S14) and (S15) with $r_{AA} = d/2$ and $D' = D_{vol}$.

The value of $\bar{k}_{irr}(t_c)$ in Table S3 was obtained with the same parameters as in Table 2, row 1 of the main text. It is in good agreement with the $k_{on}^{(mem)}$, obtained by fitting Eq (S9) to the SpringSaLaD simulations results, and characterized by low δ, i.e. the kinetics in this example is well approximated by the mass-action rate law.

Table S3. $k_{on}^{(mem)}$ from SpringSaLaD simulations vs. $\bar{k}_{irr}(t_c)$ based on Smoluchowski theory for cases with $\sigma_0 \pi h^2 > 1$

σ_0, μm^{-2}	D_{mem}, μm²/s	h, nm	$k_{on}^{(h)}$, μm²/s	$k_{on}^{(mem)}$, μm²/s	Relative RMSD	r_{AA}, μm	D', μm²/s	t_c, s	$\bar{k}_{irr}(t_c)$, μm²/s	δ
2500	0.01	20.5	0.081	0.078	0.03	0.0005	1.0	0.0113	0.078434	0.03

Note that the inequality $\sigma(t)\pi h^2 > 1$ may not hold for all levels of completion p. Indeed, the left-hand side of the inequality changes as $\sigma(t)$ drops and the effective reach of the binding side increases due to anchor diffusion ($h_{\text{eff}}(t) = h + \Delta r_{\text{diff}}$) . Therefore, the maximum $\bar{p}$ for which $\sigma(t)\pi h_{\text{eff}}^2(t) > 1$ holds true can be estimated by solving the equation, $h + 2(D_{mem} t(\bar{p}))^{1/2} = (\pi(1 - \bar{p})\sigma_0)^{-1/2}$. Function $t(p)$ is the inverse of the dimer density time dependence, which can be retrieved from the SpringSaLaD simulation results for the example in Table S3. The equation

can also be solved directly assuming applicability of Eq (S9), from which $t(p) = \frac{1}{2k_{on}^{(h)}\sigma_0}\frac{p}{(1-p)}$. The substitution yields the equation, $h(\pi(1-\bar{p})\sigma_0)^{1/2} + (2\pi D_{mem}\bar{p}/k_{on}^{(h)})^{1/2} = 1$, which can be solved exactly. For the parameters in Table S3, $\bar{p} = 0.995$. Thus, the completion level p =0.8, used for this example in SpringSaLaD and in estimating the corresponding $\bar{k}(t_c)$, satisfies the inequality $\sigma(t)\pi h_{\text{eff}}^2(t) > 1$ for all $t < \frac{1}{2k_{on}^{(h)}\sigma_0}\frac{p}{(1-p)}$.

Reversible binding of tethered monomers.
The mass-action rate law for reversible homodimerization is $\partial_t\sigma = -2k\sigma^2 + kK(\sigma_0 - \sigma)$, where K is the equilibrium dissociation constant. Its exact solution $x(t) \equiv \frac{\sigma(t)}{\sigma_0}$ satisfies the following relation,

$$\frac{x(t)-x_1}{x(t)-x_2} = \frac{1-x_1}{1-x_2}\exp(-2k\sigma_0(x_1-x_2)t), \qquad \text{Eq (S16)}$$

where x_1 is the normalized steady state, $x_1 = \sigma_{stedy\ state}/\sigma_0 = \frac{1}{2}(\sqrt{\alpha^2+4\alpha} - \alpha)$ and $x_2 = -\frac{1}{2}(\sqrt{\alpha^2+4\alpha} + \alpha)$, where $\alpha = K/2\sigma_0$. The level of completion p in the reversible case reflects how close the reaction is to its steady state, i.e. $1 - p = (x(t_c) - x_1)/(1 - x_1)$.

Based on the theory of diffusion-influenced reversible reactions (Agmon and Szabo, 1990), the weighted average of a reversible-binding reaction rate coefficient, $\bar{k}_{rev}(t_c)$, satisfies the following equation,

$$(\bar{k}_{rev}(t_c))^{-1} = (\bar{k}_{irr}(t_c))^{-1} + t_cK = (\bar{k}_D(t_c))^{-1} + \left(k_{on}^{(h)}\right)^{-1} + t_cK. \qquad \text{Eq (S17)}$$

In the second equality of Eq (S17), we used Eq (S14). It follows from the first equality of Eq (S17) that $\bar{k}_{rev}(t_c) = \bar{k}_{irr}(t_c)(1 + t_cK \cdot \bar{k}_{irr}(t_c))^{-1}$; this formula was implemented in the corresponding supplementary Excell worksheet for computing $\bar{k}_{rev}(t_c)$ based on values of $\bar{k}_{irr}(t_c)$, K, and t_c. It was used to obtained values of $\bar{k}_{rev}(t_c)$ for the examples of reversible homodimerization of rows 9 and 10 in Table 1 of the main text.
Note that the last two terms of the right-hand side of Eq (S17) are independent of diffusion. By introducing an auxiliary constant $\tilde{k}_0$ such that $(\tilde{k}_0)^{-1} = \left(k_{on}^{(h)}\right)^{-1} + t_cK$, the equation for $\bar{k}_{rev}(t_c)$ can be written in the form similar to Eq (S14): $\bar{k}_{rev}(t_c) = \tilde{k}_0\bar{k}_D(t_c)/(\tilde{k}_0 + \bar{k}_D(t_c))$, and in similarity to Eq (15), $\delta_{rev} = \bar{k}_{rev}(t_c)/\bar{k}_D(t_c)$; this formula was used for computing δ_{rev} (see the worksheet for reversible binding in the collection of supplementary files). For the reversible reactions of rows 9 and 10 in Table 1 of the main text, t_c = 0.00171 s and 0.8636 s, respectively, and for both cases $\delta_{rev} = 0.7$.

References

M. Dembo, B. Goldstein, A thermodynamic model of binding of flexible bivalent haptens to antibody. *Immunochemistry* **15**, 307-313 (1978).

P. J. Michalski, L. M. Loew, SpringSaLaD: A Spatial, Particle-Based Biochemical Simulation Platform with Excluded Volume. *Biophys J* **110**, 523-529 (2016).

O. N. Yogurtcu, M. E. Johnson, Theory of bi-molecular association dynamics iin 2D for accurate model and experimental parameterization of binding rates. *J Chem Phys* **143**, 084117 (2015).

J. Keizer, Diffusion Effects on Rapid Bimolecular Chemical Reactions. *Chem Rev* **87**, 167-180 (1987).
F. C. Collins, G. E. Kimball, Diffusion-Controlled Reaction Rates. *J Colloid Sci* **4**, 425-437 (1949).
D. C. Torney, H. M. McConnell, Diffusion-limited reaction rate theory for two-dimensional systems. *Proc Soc* A **387**, 147-170 (1983).
A. V. Barzykin, M. Tachiya, Diffusion-influenced reaction kinetics on fractal structures. *J Chem Phys* **99**, 9591-9597 (1993).
B. M. Slepchenko, L. M. Loew, Use of Virtual Cell in Studies of Cellular Dynamics. Int Rev Cell Mol Biol 283, 1-56 (2010).
D. C. Resasco, F. Gao, F. Morgan, I. L. Novak, J. C. Schaff, B. M. Slepchenko, Virtual Cell: computational tools for modeling in cell biology. *WIREs: Syst Biol Med* **4**, 129-140 (2012).
B. M. Slepchenko, J. C. Schaff, L. M. Loew, Spatial Modeling of Cellular Systems with VCell. In *Quantitative Biology. Theory, Computational Methods, and Examples of Models*, edited by B. Munsky, W. S. Hlavacek, and L. S, Tsimring. (MIT Press, Cambridge MA, 2018), Chapter 22, p. 455-468.
J. B. Pedersen, The reactivity dependence of the recombination probability. *J Chem Phys* **72**, 3904-3908 (1980).
M. Tachiya, Theory of diffusion-controlled reaction: formulation of the bulk reaction rate in terms pof the pair probability. *Radiat Phys Chem* 21, 167-175 (1983).
A. Szabo, Theory of Diffusion-Influenced Fluorescence Quenching. *J Phys Chem* **93**, 6929-6939 (1989).
N. Agmon, A. Szabo, Theory of reversible diffusion-influenced reactions. . *J Chem Phys* **92**, 5270-5284 (1990).
