# Supplementary material for: Mesoscale simulations of membrane-tethered reactions to parameterize cell-scale models of signaling
Source: Biophys J. 2026 Apr 25;125(11):2632–47. doi: 10.1016/j.bpj.2026.04.015 (PMC13351843; doi:10.1016/j.bpj.2026.04.015)
Supplement: Document S2. Article plus supplemental information [file mmc8.pdf]

Biophysical Journal
Article

# Mesoscale simulations of membrane-tethered reactions to parameterize cell-scale models of signaling

Kelvin J. Peterson,[1] Boris M. Slepchenko,[1] and Leslie M. Loew[1,*]
[1]R. D. Berlin Center for Cell Analysis and Modeling, University of Connecticut School of Medicine, Farmington, CT, USA

ABSTRACT Biochemical interactions at membranes are starting points for cell signaling. But reaction kinetics are difficult to measure on two-dimensional (2D) membranes and are usually measured in volumetric assays. Membrane tethering produces confinement and steric effects that will significantly impact binding rates; these cannot be determined by volumetric measurements. Additionally, because of the properties of 2D diffusion, bimolecular reactions may not conform to simple mass action kinetics. Here, we show how simulations using the SpringSaLaD software can be used to estimate a 2D rate constant based on a known 3D rate constant and coarse-grained molecular structures for the reactants; this approach accounts for confinement of the reaction to the near-membrane space as well as the steric environment and flexibility of the membrane-anchored binding sites. The approach is validated using theoretical solutions for dimerization in an idealized system containing a binding site at the end of a single stiff membrane anchor. With this ideal system, we also assess whether simple mass action rate constants can correctly describe the reaction rate, considering the diffusivity of the membrane anchors, the initial membrane densities of the reactants, and the desired level of completion of the reaction. We explore how factors such as molecular reach, steric effects, disordered domains, and diffusion affect the kinetics. We then apply our approach to epidermal growth factor receptor (EGFR)-mediated activation of the membrane-bound small GTPase Ras. The analysis reveals how binding of Ras to the allosteric site of SOS, a guanine nucleotide exchange factor that is recruited to EGFR, significantly accelerates Ras binding to the SOS catalytic site. A biochemical network model parametrized with the derived 2D rate constants demonstrates how recruitment of SOS via EGFR can significantly enhance Ras activation. Thus, we offer a novel method to more rigorously parameterize receptor-mediated steps in cell signaling.

SIGNIFICANCE In cell signaling, the activation of a surface receptor leads to a cascade of intracellular biochemical events. Many protein interactions occur near the inner plasma membrane surface. However, accurate rate parameters for these steps in models of signaling are rarely available because membrane-tethered reaction kinetics are difficult to experimentally measure. Here, we use a coarse-grained molecular simulator to model the kinetics of reactions between binding sites that are tethered to a membrane. We can fit these simulation outputs with two-dimensional rate laws to obtain rate constants that can be used to build complex models of cell signaling. The derived rate constants can also be analyzed to understand the key biophysical features controlling the kinetics of bimolecular membrane reactions.

## INTRODUCTION

The cell membrane responds to and integrates electrical, mechanical, and chemical signals from the extracellular environment. For chemical signals, the initial step is binding of a ligand to an external binding site on a membrane receptor protein. This triggers a chain of events that typically involves a change of state of the cytoplasmic receptor domain and subsequent recruitment of adapter proteins, enzymes, and/or cytoskeletal regulators to further evoke a cell biological response. Mathematical modeling of signaling pathways is a powerful tool to systematically organize the experimental knowledge we have about these complex systems and then develop predictions, through simulations, to inspire new experiments.[1–5]

A challenge in developing cell signaling models is the acquisition of the appropriate experimentally grounded input parameters. Often, kinetic data are available from

*Submitted January 10, 2025, and accepted for publication April 16, 2026.*
*Correspondence: les@uchc.edu
Editor: Margaret Johnson.
https://doi.org/10.1016/j.bpj.2026.04.015
© 2026 The Author(s). Published by Elsevier Inc. on behalf of Biophysical Society.
This is an open access article under the CC BY license (http://creativecommons.org/licenses/by/4.0/).

*in vitro* biochemistry, and this has served the mature field of metabolic modeling very well. However, rate parameters are less available and more difficult to measure for signaling pathways and networks. Among the key challenges is that many of the essential steps are associated with the plasma membrane, where multiple molecules are recruited before a messenger ultimately diffuses to an intracellular target (e.g., the nucleus). It is experimentally difficult to measure reaction rates on membranes because complex reconstituted bilayer methods must be used,[6,7] so available data are commonly derived from volumetric measurements. While such quantitative data are useful, it can be challenging to translate rate parameters derived from 3D solution to the very different biophysical environment of a 2D membrane.

Indeed, the biophysics of membrane-associated reactions has a long scientific history. An early focus of investigation, initiated with a classic paper by Adam and Delbruck,[8] was the difference between 2D and 3D diffusion-limited reactions; they argued that the 2-step process of absorbing a cytosolic molecule to the membrane and subsequent 2D search for an enzyme or binding partner might offer a kinetic advantage over a fully 3D search. This argument emerges from the fundamental difference between 2D and 3D diffusion: a diffusing molecule in 2D is guaranteed to eventually find its target, while no such guarantee can be made in 3D.[9]

An additional consequence of the difference between 2D and 3D diffusion is that bimolecular reactions on a surface cannot be fundamentally described by mass action kinetics.[10–16] With regard to cell signaling, theoretical analyses of reactions at membranes have been extended and elaborated to consider both diffusion-limited and reaction-limited bimolecular kinetics.[10,12,13,17] Intuitively, for reaction-limited cases (where diffusion is fast compared with the intrinsic reaction rate upon encounter) the Adam and Delbruck idea is not pertinent, and the reaction can be well described by simple mass action kinetics. In particular, Yogurtcu and Johnson[12] developed the theory to determine where the transition from reaction-limited to diffusion-limited 2D kinetics occurs—i.e., when mass action in 2D is an adequate description or when a more complex time-dependent parameterization of the rate is required.

These pioneering studies treated membrane reactions as strictly 2D surface events. However, most biological membrane-associated reactions actually occur in the immediately adjacent cytosol, with interacting sites tethered to the membrane through lipid or protein anchors. A well-known feature of anchoring bimolecular reactions to a membrane is the effect of locally increased effective concentration: compared with the same reaction by the same number of molecules within the cell volume, anchoring the reaction to the membrane generally increases concentration by confining the reaction volume to a thin layer above the membrane.[18,19] The thickness of that layer is often parametrized as $h$, sometimes called the "confinement length."[20,21] Essentially, $h$ is related to the distance the binding sites can sample above the membrane surface.[21–24] The smaller $h$, the greater is the effective concentrations of binding sites and the greater is the effective 2D affinity of the binding reaction. Estimates of $h$, and generally the interactions between membrane-bound reactants, can be derived by analyzing detailed molecular dynamics simulations of the flexibility and motions of binding domains tethered to the membrane.[21,25,26]

Recently, the concept of *molecular reach* was introduced as a more general framework for assessing how molecular structure influences the steady-state phosphorylated fraction of a membrane-bound substrate interacting with a tethered kinase.[27] The *reach* is defined as the distance of the kinase site from the membrane anchor and is directly related to $h$ when the anchor diffuses freely in the membrane. However, when lateral diffusion is restricted (e.g., within large signaling clusters such as the immune synapse), binding sites with longer *reach* may have an advantage in being able to find more binding partners; for such diffusion-limited scenarios, this increase in reach can outweigh the decrease in local concentration associated with increased $h$.[27]

Thus, it is clear from these foundational studies that the membrane diffusion and the structural features of interacting membrane-bound molecules must be considered when converting a measured 3D on rate to a 2D on rate suitable for cell-scale continuum models based on ordinary or partial differential equations (ODEs or PDEs). While detailed molecular dynamics calculations can be utilized to determine $h$ or even rate constants,[21,25,26] such intensive calculations are not always feasible to parameterize a large signaling model. In this work, we show how this can be done using simulations from SpringSaLaD[28] to derive 2D rate constants. Importantly, we are treating tethered binding sites. This is in fact the more general situation in cell signaling where a more-or-less flexible protein domain containing the binding site extends into the cytosol a significant distance from the actual membrane—typically much longer than the actual thickness of the lipid bilayer. Because our method accounts for the structure of the protein (albeit very coarsely), the distance of the binding sites from the membrane as well as other biophysical features are accounted for in the estimation of the 2D on-rate constant.

SpringSaLaD uses a series of variously sized spherical sites linked together with stiff springs to coarsely model the key structural features of macromolecules such as flexibility, excluded volume, and binding site localization. Each sphere within the molecule can be assigned its own diffusion coefficient, and Brownian diffusion is simulated via a Langevin dynamics algorithm. The molecule can be tethered to a surface, representing a membrane, via a specialized anchoring sphere that has a lateral diffusion coefficient; the rest of the molecule, including the spheres designated as binding sites, is free to explore the volume above the membrane within its reach. Naturally therefore

(and particularly advantageous for the purpose of this work), volumetric rate constants are used for bimolecular rate expressions even for membrane-bound molecules. In previous work, this feature was used to show how multivalent clustering is enhanced when one of the interacting molecules is tethered to a membrane.[29]

To determine 2D rate constants, we fit the stochastic kinetics simulated with SpringSaLaD to a deterministic mass action rate law based on the corresponding surface densities of the binding partners. Using idealized structures, we validate this procedure against theoretical calculations. We then explore how molecular structural features (e.g., tether length and stiffness, steric access), surface density, and lateral diffusion affect binding kinetics. Additionally, we develop a theory for membrane-tethered reactions, similar to that of Yogurtcu and Johnson for purely 2D reactions,[12] to assess the appropriateness of a mass action rate constant for varying tether lengths, anchor diffusion coefficients, reaction rates, initial surface densities, and the desired level of completion of the reaction. Then, as a biologically relevant example, we apply this approach to recruitment of SOS to the epidermal growth factor receptor (EGFR). SOS is the G-protein exchange factor (GEF) for Ras,[30] and a better understanding of SOS activation of Ras emerges from this analysis. Thus, we offer a procedure for parameterizing membrane kinetics that serves to bridge molecular to cell-scale simulations of cell signaling.

## MATERIAL AND METHODS

All simulations were performed with SpringSaLaD v. 2.3.4 (https://vcell.org/ssalad). While the full physics and math behind the calculations performed by SpringSaLaD can be found in the original publication,[28] we provide a brief overview of its functionality. A molecule in SpringSaLaD is approximated as a series of variably sized hard spheres, representing protein domains, connected by stiff springs. Random forces (chosen from a normal distribution with a variance determined from the diffusion coefficient assigned to each sphere by the user) impinge on each sphere at each time step and are transmitted to neighboring spheres through the stiff springs. The software models membranes as a planar surface within the volume of the 3D simulation domain. The membrane can have embedded spheres that serve as diffusing anchors (with user-assigned 2D diffusion coefficients) linked to spheres that extend into the volumetric space. Each sphere may also be designated as the site of a reaction. The user can input macroscopic rate constants for reactions, and the software converts these to reaction probabilities within each time step; for bimolecular reactions, this conversion also depends on the radii of the spherical binding sites and the sum of their diffusion coefficients. SpringSaLaD allows users to manually edit molecules to fully customize structure and molecule flexibility. The volumetric rate constant of binding between sites tethered to the membrane is assumed to be the same as that of the untethered sites. Geometrical and steric constraints at binding sites can be modeled by the appropriate placement of neighboring spheres adjacent to the respective binding sites within the reacting molecules. To build coarse-grained molecular structures used in SpringSaLaD for Figure 6, we used AlphaFold2[31,32] to generate PDB file estimates of protein structures for EGFR, Grb2, SOS, and Ras via input of entire amino acid sequences. These PDB files are converted to highly coarse-grained molecular models via the mol2sphere[33] utility embedded in SpringSaLaD. In some cases, we manually edited the structures to capture their essential features from measurements on the PDB structures, as visualized in PyMol (Schrödinger, Inc.). In particular, for SOS we subdivide the CDC25 and REM domains into multiple smaller spherical sites with only two sites (the allosteric and catalytic sites for Ras) capable of participating in a binding reaction. This process maintains the structural characteristics of these domains, while ensuring that the binding radius of the domain is not artificially inflated. Modeling disordered regions, such as the proline rich motif (PRM) region of SOS, can be challenging due to low confidence in the AlphaFold2-generated geometry of these regions. To model disordered domains, we use PyMOL to measure the length of entire straight chain amino acid sequences and then model this sequence in SpringSaLaD using 1.0-nm-diameter sites connected by 3.1-nm linkers (in SpringSaLaD, a string of relatively small spheres connected by links that are larger than the sphere diameters can be used to model flexible [i.e., disordered] domains). Binding reactions in all simulations have rates input in terms of $\mu M^{-1}s^{-1}$; in our simulations, successful binding results in 1-nm links between the surfaces of the spherical sites. The default simulation time step of 10 ns was sufficiently accurate for all the SpringSaLaD simulations in this work, as checked by testing 2-ns timesteps and noting no significant differences; we recommend this test be applied by all users, at least for the simulation scenarios with the fastest rates. Simulations for Tables 1 and 2 and Figures 1–3 and 5, were run with 40 molecules, while Figure 6 used 200 molecules of Ras and 20 complexes of EGFR-Grb2-SOS to proportionally reflect the difference in concentrations between Ras and SOS molecules *in vivo*.[34,35] The 3D computational domain had a z coordinate of 100 nm, with the membrane placed in the XY plane at Z = 10 nm; the boundaries at the edges of the domain have reflective boundary conditions. X and Y coordinates were set to provide the desired initial surface densities. Initial placement of the molecules was random and different for each trajectory. A set of 100 trajectories is simulated in parallel using the Center for Cell Analysis and Modeling High Performance Compute Cluster (https://health.uconn.edu/high-performance-computing/resources/). One run for Tables 1 and 2 required approximately 3 h, and one run for Figure 6 required approximately 30 h. SpringSaLaD input files are in supplemental information and provide all the geometric details for the molecules in each computational experiment.

**Table 1. Results for single stiff link, binding site $D_{vol}$ = 1.0 μm²/s, $h$ = 0.0055 μm:**

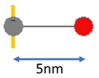


| Row | $k_{on}^{(vol)}$ (μM$^{-1}$·s$^{-1}$) | Initial surface density (molecules/μm$^2$) | $D_{mem}$ (μm$^2$/s) | $k_{on}^{(h)} = \frac{k_{on}^{(vol)}}{h \cdot 602.2}$ (μm$^2$·molecules$^{-1}$·s$^{-1}$) | $k_{on}^{(mem)}$, SpringSaLaD fit (μm$^2$·molecules$^{-1}$·s$^{-1}$) | Relative RMSD (%) | $\bar{k}$, Theory (μm$^2$·molecules$^{-1}$·s$^{-1}$) |
|---|---|---|---|---|---|---|---|
| 1 | 0.01 | 2500 | 0.01 | 0.003 | 0.0029 | 0.55 | 0.0025 |
| 2 | 0.01 | 2500 | 1.0 | 0.003 | 0.0031 | 0.55 | 0.0030 |
| 3 | 0.01 | 25 | 0.01 | 0.003 | 0.0024 | 0.53 | 0.0023 |
| 4 | 0.01 | 25 | 1.0 | 0.003 | 0.0030 | 0.48 | 0.0030 |
| 5 | 1.0 | 2500 | 0.01 | 0.30 | 0.084 | 8.6 | 0.023 |
| 6 | 1.0 | 2500 | 1.0 | 0.30 | 0.26 | 1.6 | 0.27 |
| 7 | 1.0 | 25 | 0.01 | 0.30 | 0.018 | 3.9 | 0.011 |
| 8 | 1.0 | 25 | 1.0 | 0.30 | 0.20 | 2.2 | 0.25 |
| 9 | 1.0 ($K_D$= 3333 molecules/μm$^2$) | 2500 | 0.01 | 0.30 | 0.13 | 11 | 0.033 |
| 10 | 1.0 ($K_D$= 33.33 molecules/μm$^2$) | 25 | 0.01 | 0.30 | 0.021 | 5.3 | 0.0095 |

The mean time dependence of dimer surface density $[dimer]_t$, based on 100 SpringSaLaD runs for each of the parameter sets in Tables 1 and 2 and Figure 6, was fitted with a deterministic 2D mass action rate law to obtain $k_{on}^{(mem)}$. For irreversible dimerization (Table 1, first 8 rows; Table 2), a fit to the analytical mass action expression for the appearance of dimer (Equation 1) was obtained with the Excel solver.

$$[dimer]_t = [dimer]_\infty - \frac{[dimer]_\infty}{\left(4 * k_{on}^{(mem)} * [dimer]_\infty * t\right) + 1} \quad \text{(Equation 1)}$$

The values of $k_{on}^{(mem)}$ were all confirmed with COPASI.[36] COPASI also provided the standard deviations (SDs) of the derived rate constants, which were all less than 0.5% of the determined $k_{on}^{(mem)}$. Importantly, this SD only estimates

**Table 2. Dimerization of idealized structure types**

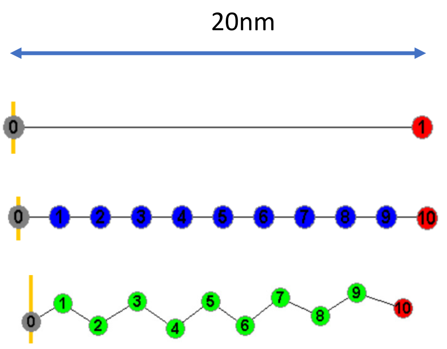


| Structure | $k_{on}^{(h)} = \frac{k_{on}^{(vol)}}{h \cdot 602.2}$ (μm$^2$·molecules$^{-1}$·s$^{-1}$) | $k_{on}^{(mem)}$ SpringSaLaD fit (μm$^2$·molecules$^{-1}$·s$^{-1}$) | Relative RMSD |
|---|---|---|---|
| Single site, stiff | 0.081 | 0.078 | 2.9% |
| Steric sites, stiff | 0.081 | 0.061 | 4.6% |
| Steric sites, flexible | 0.081 | 0.064 | 5.7% |

$D_{mem}$ =0.01μm$^2$/s, binding site $D_{vol}$ =1.0μm$^2$/s, $k_{on}^{(vol)}$ = 1 μM $^{-1}$s$^{-1}$, $h$=0.0205 μm, surface density = 2500 molecules/μm$^2$.

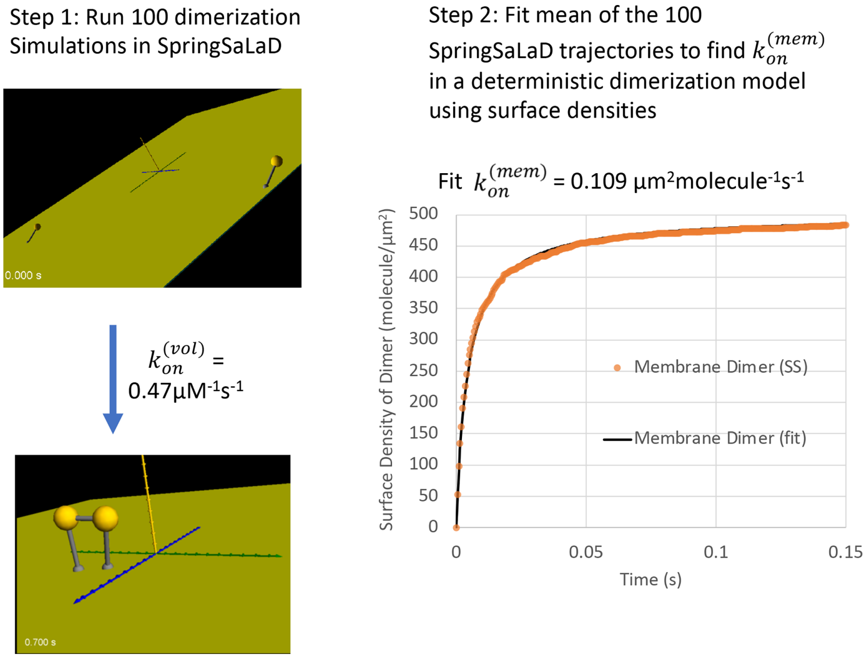


Figure 1. Workflow for finding membrane bimolecular binding rate constants ($k_{on}^{(mem)}$) in terms of surface densities. Step 1 is to run 100 SpringSaLaD trajectories with the coarse-grained model of the binding partners. Illustrated are a pair of simple monomers (*top*) consisting of 2-nm-diameter spheres (yellow) tethered to a 1-nm membrane anchor sphere (gray) with a 5-nm link; at the bottom, the product dimer is depicted. Step 2 consists of fitting the average of 100 outputs from stochastic SpringSaLaD simulations to a deterministic (ODE) nonspatial model of surface-bound dimerization.

the uncertainty in $k_{on}^{(mem)}$ related to the finite curvature of the objective function near its minimum—not how well the simulation results are fit by the mass action model. The goodness of fit is characterized by the minimum of the objective function. In our study, the objective function was defined as the relative root mean squared deviation (RMSD) of the mass action equation (Equation 1) from the dimer surface density yielded by SpringSaLaD, as described in the results. For reversible dimerization (Table 1, rows 9 and 10) and for the fits in Figure 6, we used the COPASI parameter estimation tool within Virtual Cell (VCell)[37,38] as well as COPASI itself for confirmation and to determine the SD. The former can be accessed in the VCell published BioModel "Peterson Table 1 reversible." All these results with some further analysis can also be found in the spreadsheets included in the supplemental information. The VCell model related to Figure 6 can be found in the VCell database with the name "Peterson Figure 6: Ras-SOS_Binding_fit_to_SpringSaLaD." The model related to Figure 7 can be available in the VCell database as "SOS_Recruitment_Ras_Binding". Access to the VCell database is through the VCell client, which can be downloaded at https://vcell.org/run-vcell-software.

## RESULTS

### The general approach

For bimolecular reactions, SpringSaLaD determines the microscopic probability of two binding sites forming a bond as they diffuse within a reaction radius that is slightly larger than the sum of their physical radii. The input to the algorithm is simply the macroscopic volumetric on-rate constant ($k_{on}^{(vol)}$, units of $\mu M^{-1}s^{-1}$) and the diffusion coefficient of the individual spheres. Full details on the derivation of the reaction probability and a thorough validation of its accuracy can be found in the original paper describing SpringSaLaD.[28] Importantly for the purposes of this work, the rate of binding for sites that happen to be tethered to a membrane is still treated as volumetric, because the spherical sites are located in the volume compartment even while they are constrained with links to the 2D membrane surface.

Figure 1 illustrates this for a simple dimerization reaction where the two yellow binding sites are tethered to the membrane anchor (gray sphere) by a 5-nm link; in these simulations, both the anchor and tether sites are given identical diffusion coefficients of 1 $\mu m^2$/s. We ran 100 SpringSaLaD simulations each with 40 dimerizing molecules (Figure 1 only shows two molecules for clarity). The mean trajectory for these 100 runs is then fitted with a deterministic mass action membrane binding model in terms of surface densities using either COPASI or Virtual Cell (although an analytical solution can also be fit for simple dimerization) (step 2 in Figure 1). The output binding rate constant, $k_{on}^{(mem)}$ in units of $\mu m^2 molecule^{-1}s^{-1}$, can then be used to parameterize larger deterministic or stochastic models (that utilize mass action rate constants as inputs) with molecule numbers (>1000) or timescales (>10 s) that would be too large for even highly coarse-grained molecular simulators like SpringSaLaD. Also, we emphasize that binding rates are typically determined experimentally using *in vitro* volumetric measurements; that SpringSaLaD uses volumetric rate constant inputs makes the procedure in Figure 1 especially appropriate and convenient. The results in Figure 1 show that a 2-nm-diameter binding site with an on-rate constant for dimerization

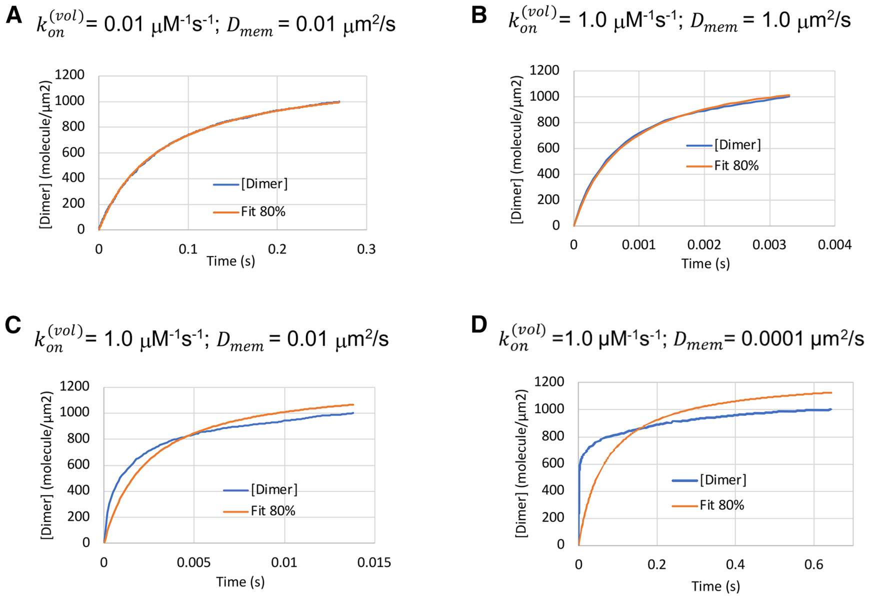


Figure 2. Examples of SpringSaLaD simulation data and their fit to surface-confined mass action dimerization kinetics. The initial surface density of monomers in each case is 2,500 molecule/μm$^2$. The diffusion coefficient of the anchor and the volumetric binding rate constant of the binding sites are indicated above each graph, (A), (B), and (C) corresponding respectively to rows 1, 6, and 5 of Table 1. (D) shows a result with diffusion-limited kinetics, $D_{mem}$ = 0.0001 μm$^2$/s, exemplifying the poor fit to mass action.

of 0.47 μM$^{-1}$s$^{-1}$ and tethered to a membrane surface through a 5-nm link can be modeled as a 2D surface reaction with an on-rate constant, $k_{on}^{(mem)}$, of 0.109 μm$^2$molecule$^{-1}$s$^{-1}$. Using idealized models, we now explore how various structural and biophysical parameters control $k_{on}^{(mem)}$ and when mass action rate constants may not be appropriate to describe membrane-associated binding kinetics.

## Validation

To develop a rigorous validation of the method, we needed to devise an idealized system that would be theoretically tractable and also qualitatively intuitive. The goal was to test the procedure described in the previous section for various combinations of volumetric dimerization on-rate constant, 2D diffusion constantof the membrane anchor, and initial surface density.

To achieve this, we analyze the case of a 5-nm stiff tether between a 1-nm-diameter binding sphere and a membrane anchor site. The diffusion coefficient for the binder sphere is set to 1 μm$^2$/s. Table 1 gives results for all combinations of two volumetric on-rate constants, two surface densities, and two membrane diffusion coefficients. All the dimerization rate laws are irreversible except for the last two rows, where the equilibrium dissociation constant, $K_D$, is indicated. The 2D on-rate constant derived by fitting the SpringSaLaD simulation, $k_{on}^{(mem)}$, is in the fifth column. For consistency, all these fits are performed for kinetics at 80% completion (for practical applications of our method, shorter or longer levels of completion may be more appropriate, as discussed below). For comparison, we also provide the 2D on-rate constant, $k_{on}^{(h)} = k_{on}^{(vol)}/h$, where $h$ is the linker length plus the radius of the binder sphere. If $h$ is in units of μm, dividing by a unit conversion factor of 602.2 converts $k_{on}^{(h)}$ from units of μM$^{-1}$s$^{-1}$ to units of μm$^2$molecules$^{-1}$ s$^{-1}$; $k_{on}^{(h)}$ is an equivalent 2D binding rate constant of freely diffusing monomers confined within a thin volume with a height $h$ adjacent to the membrane. The Relative RMSD column provides the square root of the sum of the squared deviations of the fitted values from the SpringSaLaD values divided by the sum of the squared SpringSaLaD values; this metric provides a measure of the goodness of fit to the bimolecular mass action rate law and was minimized to obtain $k_{on}^{(mem)}$. Examples of the fits are shown in Figure 2. (The data and plotted fits for all the entries in Table 1 can be found in Supporting file "Peterson Table 1 Fits.xlsx.") The last column, $\bar{k}$ is based on an analysis of the time-weighted average of the solution to the Smoluchowski theory for the tethered binding site anchored to the membrane; it will be discussed further, below.

### *The SpringSaLaD fits for reaction-limited binding are consistent with mass action*

Let us start by considering the first 4 rows of Table 1 where the volumetric on-rate constants $k_{on}^{(vol)}$ (and therefore $k_{on}^{(h)}$) are slow; these represent reaction-limited scenarios where diffusion in the membrane should not severely affect the kinetics, and therefore, mass action should be a good approximation. We looked at two anchor diffusion coefficients corresponding to that of a large transmembrane protein domain ($D_{mem}$ = 0.01 μm$^2$/s) and a lipid anchor ($D_{mem}$ = 1 μm$^2$/s); in all cases, the diffusion coefficient of the binder $D_{vol}$ = 1 μm$^2$/s. Thus, for the cases where $D_{mem}$ = 0.01 μm$^2$/s, the anchor effectively acts as a pivot, and the binder rapidly moves within a hemispherical shell to effectively create a reaction region with a thickness slightly larger than the 1-nm diameter of the binder sphere.

Because the region of spatial overlap of the two shells where the binding may occur is restricted, it might seem surprising that $k_{on}^{(mem)}$ is so closely approximated by $k_{on}^{(h)}$ in the first row of Table 1. However, as detailed in the Supporting Text I, the reaction probability is enhanced because of the effectively higher density of binding sites within this restricted region, which compensates for the smaller spatial overlap. Thus, the calculations in the Supporting Text I both explain and validate the SpringSaLaD results for the cases of slow anchor diffusion. The second row of Table 1 corresponds to the case where the anchor and the binder have the same fast diffusion. In this scenario, that $k_{on}^{(mem)} \cong k_{on}^{(h)}$ is intuitive, because the effect of the tether in this case essentially reduces to confining the binders within the layer adjacent to the membrane.

### *When diffusion becomes limiting, $k_{on}^{(mem)}$ is dependent on initial surface density and deviates from $k_{on}^{(h)}$*

Of course, when diffusion is limiting, it is known that the rates of membrane reactions are susceptible to deviations from a simple mass action rate law[10,12,16,39–43]; this can manifest itself as significant changes of the apparent rate constant as a function of initial surface density. We probed for this by decreasing the initial density by a factor of 100. The combinations of $D_{mem}$ and $k_{on}^{(vol)}$ in the third and fourth rows of Table 1 resulted in relatively small changes in $k_{on}^{(mem)}$, indicating that the mass action rate law applies to these cases. To further test our approach when diffusion may become limiting, we increased the $k_{on}^{(vol)}$ by a factor of 100 in the lower half of Table 1. Clearly, for the case of $D_{mem} = 0.01\ \mu m^2/s$, there is a strong dependence on surface density (Table 1 rows 5 and 7; see also the fit in Figure 2C). The, $k_{on}^{(mem)}$ values for these 2 cases are very different from each other and also from $k_{on}^{(h)}$.Thus, the combination of $k_{on}^{(vol)} = 1.0\ \mu M^{-1}s^{-1}$ and $D_{mem} = 0.01\ \mu m^2/s$ present cases where a simple mass action rate law may not be appropriate; this is even more apparent for the case of $D_{mem} = 0.0001\ \mu m^2/s$ in Figure 2D, where the deviation of the SpringSaLaD simulation from the best fit to mass action dimerization is very severe. The simulation results are initially faster and then ultimately slower than the best fit that assumes mass action kinetics. This is because the monomers whose binding sites are initially close to each other (effectively within the "reach" of the tether) will react, but leave behind depletion zones where monomers are too far away from potential binding partners.[41,42] These isolated monomers can be discerned toward the middle of Video S1, which presents an example trajectory for the first 150 ms of the highly diffusion-controlled case of Figure 2D. It starts with 40 monomers randomly placed on a 160 nm × 100 nm membrane (corresponding to an initial density of 2,500 monomers/μm$^2$). It shows that the first 7 dimers form within the first 2 ms and correspond to the pairs of monomers that are relatively close to each other (i.e., "within reach") at time 0 s. The slow anchor diffusion keeps the remaining 26 monomers from encounters, until by 20 ms, only 2 more dimers are formed, after which long-lived "depletion zones" become established; by the end of the remaining 130 ms in the movie, only 3 more dimers have formed. Given that this is only one trajectory, it is remarkably consistent with the mean of 100 trajectories in Figure 2D and that there are 2 timescales corresponding to approximately reaction limited at the very beginning and diffusion limited for most of the time course—decidedly not well described by Equation 1. But as will be discussed below, it might still be a reasonable approximation to use a mass action rate expression within a kinetic network model for a given initial condition and desired level of completion. Importantly, these results reflect the ability of the SpringSaLaD simulations to capture the physics of membrane-tethered reactions.

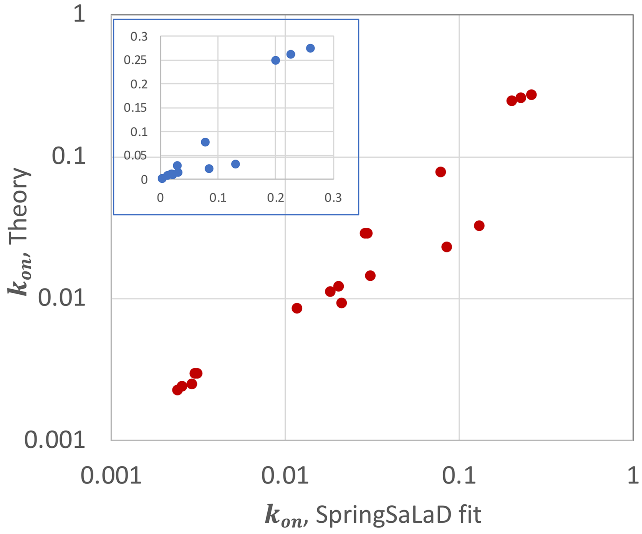


Figure 3. Correlation between $\bar{k}$ and $k_{on}^{(mem)}$ for 19 different conditions. The main plot is log-log to display the full range of rate constants; the insert is a linear plot. Full lists of all the additional conditions are in Table S2 of Supporting Text II.

### *SpringSaLaD simulations are consistent with the thermodynamic law of mass action*

In the last 2 rows of Table 1, we considered reversible reactions for the diffusion-limited scenario of fast $k_{on}^{(vol)}$ (1.0 $\mu M^{-1}s^{-1}$) and slow $D_{mem}$ (0.01 $\mu m^2/s$). For the SpringSaLaD simulations, we chose volumetric dissociation rates, $k_{off}^{vol}$, of 1,000 $s^{-1}$ and 10 $s^{-1}$, respectively, for the 2,500 and 25 monomer/μm$^2$ initial densities. The respective mean steady-state densities of dimers in the SpringSaLaD simulations were 570 and 5.7 molecules/μm$^2$ (see the full set of data and fits in the spreadsheet Peterson Table 1 Fits.xlsx). These values are consistent with the *thermodynamic* law of mass action[11] and thus further validate the SpringSaLaD simulations. Namely, the ratio of the squared monomer surface density to the dimer surface density is equal at steady state to the equilibrium dissociation constant

$K_D$, which is determined by the strength of the interaction between the monomers and thus is independent of reaction kinetics. $K_D$ in each case is thus equal to the ratio of the intrinsic on and off reaction rate constants, uninfluenced by diffusion. In our case, $K_D = k_{off}^{vol}/k_{on}^{(h)}$, because the interactions of the binding sites in the 3D volume above the membrane obey mass action kinetics, and $k_{on}^{(h)}$ is the 3D association rate constant adjusted for the restricted volume above the membrane. It is gratifying (and further validation) that the SpringSaLaD simulations produce these correct steady-state surface densities even when, as in the last 2 rows of Table 1, the kinetics are far from reaction limited.

### *Dimerization rates are highly sensitive to the linker length*

Table 2 provides results for three computational experiments in which structural features of the SpringSaLaD molecules are varied. These are all for dimerization reactions where the maximum distance between the membrane and the binding site is four times longer than in Table 1: $h$ = 0.0205 μm (linker length of 20 nm and binding site radius of 0.5 nm). The $k_{on}^{(h)}$ is therefore a factor of ~4 slower than that in Table 1 for the same $k_{on}^{(vol)}$ of 1 $\mu M^{-1}s^{-1}$. The SpringSaLaD simulations were carried out with a slow membrane-anchor diffusion coefficient, $D_{mem}$ = 0.01 $\mu m^2/s$, to model a transmembrane protein domain. However, the longer reach makes the slow anchor diffusion less limiting, and we find that $k_{on}^{(mem)}$ is much closer to $k_{on}^{(h)}$ than the comparable condition of row 5 in Table 1. This idea is quantitatively analyzed in supporting textI I and will be further discussed below. The second row in Table 2 shows results for a structure where additional spherical sites are introduced between the membrane anchor and the binding site to model the space occupied by a cytosolic protein sequence; this steric effect results in a small decrease in $k_{on}^{(mem)}$. This decrease is diminished when flexibility is introduced by allowing the spherical sites to be pivot points in the third row of Table 2; this is how disordered domains may be modeled in SpringSaLaD. Overall, for these idealized structures and simple dimerization, the fitted on-rate constants in Table 2 are relatively close to $k_{on}^{(h)}$.

### *$k_{on}^{(mem)}$ correlates well with $\bar{k}$, derived from Smoluchowski theory for dimerization of membrane-tethered binding sites*

Qualitatively, one can consider the overall rate coefficient, $k_{on}$, to be affected by the intrinsic binding occurring once the sites are close enough to bind and characterized by the rate constant $k_0$, and by diffusion-influenced reactant encounters characterized by their corresponding rate coefficient $k_D$. In 3D, $k_D$ can be treated as essentially time independent (see detailed discussion and Figure S7A in Supporting Text, part II),[14] and so is $k_{on}$, which in this case is computed as $k_{on} = \frac{k_D k_0}{k_D + k_0}$.[15] As discussed above, simple mass action kinetics are not appropriate for 2D bimolecular reactions within a surface because $k_D$ becomes a function of time.[10,12,14,15] Specifically, in the diffusion-limited regimes with $k_D(t) \ll k_0$, the $k_{on}$ will be largely determined by $k_D(t)$ and thus will depend on time. For the idealized case of a single binding site tethered to the membrane by a single link, as in Table 1 and the first row of Table 2, we were able to adapt Smoluchowski theory for the rate of diffusion-limited encounters in 2D[10,12,14,15] to derive $k_D(t)$ for the case of tethered binders, as detailed in Supporting Text II. With $k_D(t)$ in hand for any $D_{mem}$, and taking the intrinsic reaction rate constant to be $k_{on}^{(h)}$, we could derive a theoretical time-weighted average rate constant, $\bar{k}$, for a desired level of completion (i.e., $\bar{k}(t_c)$, where $t_c$ is the time it takes to reach the level of completion); see the derivation leading up to Equation S14 in Supporting Text II. Figure 3 shows there is a good correlation between $\bar{k}$ and $k_{on}^{(mem)}$; Figure 3 includes all 10 cases in Table 1, an additional 8 intermediate cases of surface density and $D_{mem}$, and the first row of Table 2. This is all the more remarkable because different theoretical approaches to determine $\bar{k}$ were required for, respectively, the irreversible 5-nm structures (16 conditions), the irreversible 20-nm structure (Table 2, first row), and the 2 reversible 5-nm cases (Table 1, rows 9 and 10); all of these are described in Supporting Text II.

## Considerations for using $k_{on}^{(mem)}$ in models of cell signaling

The previous section examined the rate constant, $k_{on}^{(mem)}$, derived from the SpringSaLaD fits to Equation 1 in a variety of scenarios based on idealized structures. We showed that $k_{on}^{(mem)}$ follows theory, both qualitatively and quantitatively even for diffusion-limited reactions on membranes, where the results are far from the simple mass action rate law. In this section, we seek to understand how $k_{on}^{(mem)}$ may be a sufficiently good approximation to be useful in modeling cell signaling, even for situations where diffusion may influence the kinetics.

In a signaling model, the dynamic levels of molecular species feeds into other components of a reaction network. For each of the individual reactions in the network, if the concentrations of reactant and product are reasonably well estimated, the model can provide useful predictions. How well $k_{on}^{(mem)}$ for our mass action rate expression predicts the levels of reactant and product is given by the relative RMSD. Consider row 5 of Table 1. For this set of conditions, $k_{on}^{(mem)}$ (0.084 $\mu m^2 molecule^{-1}s^{-1}$) is significantly lower than $k_{on}^{(h)}$ (0.30 $\mu m^2 molecule^{-1}s^{-1}$), which can be taken as the rate coefficient in the limit of fast diffusion. Despite this, the fit to mass action kinetics appears to be reasonably good (Figure 2C) with a corresponding relative RMSD of 8.6%. While there is an underestimate of the fitted curve at short times and overestimate at long times, at any given time point, the estimated surface density of product is on average within 8.6% of the actual SpringSaLaD simulation.

Therefore, even when diffusion severely influences the rate, we might be able to use the derived $k_{on}^{(mem)}$ within a signaling model, where an uncertainty of 10% is often quite acceptable. We will now further analyze why the relative RMSD can be small even when diffusion is limiting and the true rate coefficient is time dependent.

The key to understanding the small relative RMSD for cases where diffusion is limiting lies in an examination of the time dependence of $k_D(t)$. This was derived from Smoluchowski theory for the idealized tethered binders in Supporting Text II and is illustrated in Figure 4 for the conditions of rows 5 and 7 of Table 1. We focus on rows 5 and 7 because the conditions are the same for each except for the initial concentrations (2,500 and 25 molecule/μm$^2$, respectively); furthermore, their $k_{on}^{(mem)}$ values (0.084 and 0.018 μm$^2$molecule$^{-1}$s$^{-1}$, respectively) are each quite far from $k_{on}^{(h)}$, indicating largely diffusion-limited kinetics. Strikingly, the relative RMSD for line 7 (3.9%) is smaller than for line 5 (8.6%) even though $k_{on}^{(mem)}$ for line 7 is much further from $k_{on}^{(h)}$. The results of Figure 4 show that most of the time dependence of $k_D(t)$ occurs during the very beginning of the kinetics. When the kinetics is lengthened simply by starting with lower initial surface densities (Figure 4, right panel), $k_D(t)$ stays relatively constant over much of the time course. This explains why the longer timescale associated with the kinetics of lower initial surface density results in a near constant contribution of $k_D(t)$ to the overall $k_{on}$ and the reasonable fit to a constant $k_{on}^{(mem)}$ in Equation 1. Importantly, because $k_{on}^{(mem)}$ is so dependent on initial conditions when diffusion becomes limiting, these must be specified in the SpringSaLaD simulations and be based on the corresponding conditions in the network model that is being targeted for parametrization.

Thus far, we have compared all our results for the same level of completion—chosen arbitrarily at 80%. But, it is important to appreciate that $k_{on}^{(mem)}$ will indeed be sensitive to the level of completion for diffusion-influenced kinetics. This is intuitive based on the idea that at the initial stages more tethered binding sites will be close enough to react with a reaction-limited rate, but as "depletion zones" develop, diffusion will increasingly limit the rate of reaction

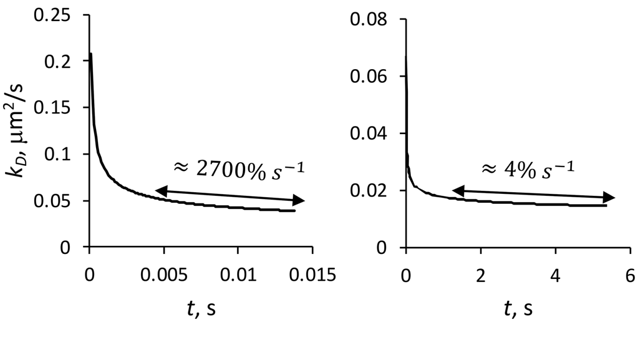


Figure 4. Time dependence of $k_D$ in 2D. Time dependence of $k_D$ in 2D for the conditions of rows 5 (*left*) and 7 (*right*). The abscissa is time in seconds.

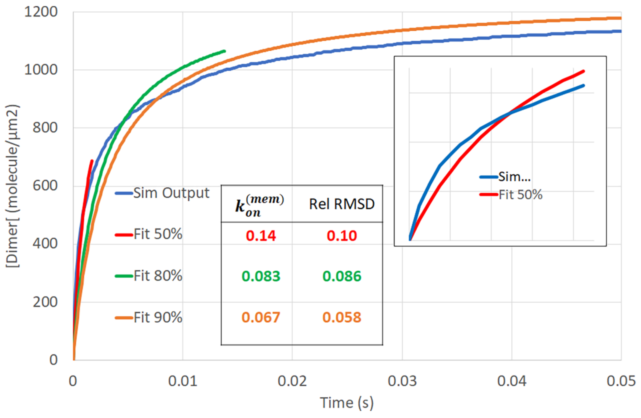


Figure 5. Fits to different levels of completion for the conditions of row 5 of Table 1. The inset is an expansion of the first 1.7 ms.

(see the results described in relation to Figure 2D and Video S1). Figure 5 examines this issue directly for the diffusion-influenced case of row 5 in Table 1, where in addition to the fit to 80% completion, we also examined the fits to 50% and 90%. The earlier the fit is terminated, the closer is $k_{on}^{(mem)}$ to $k_{on}^{(h)}$, the reaction-limited rate. On the other hand, the fits out to longer levels of completion display better relative RMSD; this is, of course, consistent with our discussion of Figure 4, which shows that at longer times the diffusion-limited component of the rate coefficient varies very slowly. These considerations further underline the importance of specifying the pertinent conditions as precisely as possible when determining a diffusion-influenced $k_{on}^{(mem)}$ for use in a reaction network model.

But, let us return to the situation where the kinetics are essentially reaction limited (i.e., where the diffusion of the membrane anchor is fast on the timescale of the intrinsic reaction rate). This is the case for the first 4 rows of Table 1 and for Table 2, where $k_{on}^{(mem)}$ is close to $k_{on}^{(h)}$. In such cases, it should be possible to use a single value of $k_{on}^{(mem)}$ for a large range of initial surface densities and levels of completion. Of course, in Tables 1 and 2, $k_{on}^{(h)}$ was computed from the value of $h$ assigned to these idealized structures. In a more realistic application of the method with coarse-grained molecular structures (e.g., in the next section), the value of $h$ may be difficult to estimate; furthermore, the reaction may be between sites on different molecules (as opposed to homodimerization as in Tables 1 and 2). However, it should be possible to determine whether $k_{on}^{(mem)}$ is close to the reaction-limited rate constant simply based on the criteria outlined in this section: that it is insensitive to initial surface density and to the level of completion. Indeed, comparing row 2 to row 4 (where anchor diffusion is high) shows that a 100-fold decrease in surface density does not appreciably affect $k_{on}^{(mem)}$; however, there is a difference between rows 1 and 3, where the $D_{mem}$ is 100-fold lower. Another criterion for how close $k_{on}^{(mem)}$ is to the reaction limit is its sensitivity to the level of

completion; Figure 5 demonstrates a high sensitivity for the condition of fast $k_{on}^{(h)}$ and slow $D_{mem}$. On the other hand, the opposite conditions in row 2 of Table 1 give the same $k_{on}^{(mem)}$ of 0.0031 for 50%, 80%, and 90% levels of completion (Supporting file Peterson Table 1 Fits.xlsx). Finally, a third test that could be performed is to see how sensitive the derived $k_{on}^{(mem)}$ is to a change in $D_{mem}$; this is exemplified by comparing rows 1 and 2, which show that this scenario is insensitive to $D_{mem}$ and therefore can be taken to be reaction limited (compare, on the other hand, rows 5 and 6). All of the above tests can be applied to complex coarse-grained structures where $k_{on}^{(h)}$ cannot be determined *a priori*.

We now illustrate our method by applying it to a real signaling module that has been widely investigated because of its importance in cancer biology.

## Application of the method to interaction of receptor-bound SOS with Ras

Until now, we have employed idealized molecular structures to validate our method and to learn more about biophysical principles that control the on-rate constants of binding sites tethered to membranes. We now illustrate the application of this approach to a biologically relevant example, namely the interaction of the GEF SOS with the lipid-anchored small G-protein Ras.[30]

SOS has two binding sites for Ras: an allosteric site and a catalytic site. When a Ras molecule binds to the allosteric site, it increases the GEF activity of the catalytic site.[44] Additionally, before SOS binds to Ras, it is first recruited to an active receptor tyrosine kinase (RTK) through an adapter protein; the adaptor binds to a PRM on SOS via a SH3 domain and to a phosphorylated tyrosine via a SH2 domain. One such RTK is the EGFR, and one such adaptor protein is Grb2.[45] Once SOS is bound to Grb2, it becomes membrane tethered, and its interaction with Ras is facilitated.[44] However the complex mechanistic details of this system are still emerging.[46]

We asked the limited question of how binding of Ras with the receptor-associated SOS catalytic site might depend on whether SOS is prebound to Ras at the allosteric site. Ras is a lipid-anchored protein, so we reasoned that binding of Ras to the allosteric site of SOS would bring the SOS catalytic site closer to the membrane to enhance binding to a second Ras and subsequent exchange of GDP for GTP. Just how large an effect this is may be estimated by the procedure developed above, with the results shown in Figure 6.

We developed molecular models with the aid of the mol2-sphere[33] utility within SpringSaLaD and were guided by AlphaFold 2 atomic structure predictions[31,32]; all the site diameters and linker lengths are available in the SpringSaLaD input file included in the supplemental information; snapshots of the structure are shown in Figure 6. The top of Figure 6 displays results for binding of SOS-Grb2-EGFR to Ras at the SOS catalytic site; the bottom shows results for the same reaction, except SOS-Grb2-EGFR had been first bound to a Ras molecule at the SOS allosteric site. The input rates shown at the top of Figure 6 are based on experimentally measured data[30] and are applied to both of the reactions. Special considerations were applied to ensure accuracy of the on-rate constant, which was parameterized via an initial cytosolic reaction where the output rate

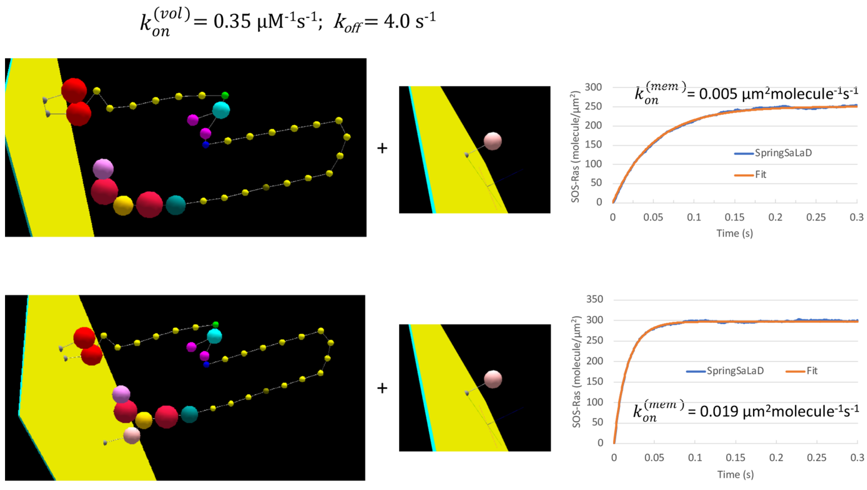


Figure 6. Membrane binding of Ras to the catalytic site of receptor-bound SOS. Top: direct binding. Bottom: after pre-binding of Ras to the allosteric site. The molecular structures are approximated by using the SpringSaLaD 3D editing utility based on atomic structures derived from AlphaFold2. The top left structure is an EGFR cytoplasmic domain anchored to the membrane (red kinase domain, followed by yellow disordered tail capped by a phosphotyrosine in green); the latter is linked to a cyan SH2 domain in Grb2; one of its magenta SH3 domains is linked to an olive PRM on the end of the disordered region of SOS; the violet SOS sphere is the binding site for the catalytic domain of Ras. The bottom left structure is identical, except that the pink allosteric site on SOS is prebound to Ras. The Ras structures are shown in the center with the pink spheres serving as the binding sites. The input rate constants for the SpringSaLaD simulations are shown at the top, corresponding to the volumetric on-rate constant for Ras binding to the catalytic site of SOS. The EGFR anchor diffusion coefficient is 0.01 μm$^2$/s. All other site diffusion coefficients are 1.0 μm$^2$/s. For each condition, 20 EGFR-Grb2-SOS molecules react with 200 Ras molecules on a 250 nm × 250 nm membrane surface to generate 100 SpringSaLaD trajectories. Their means were fitted to a deterministic 2D rate law to derive $k_{on}^{(mem)}$, fixing $k_{off}$ at 4.0 s$^{-1}$; results for the 2 conditions are shown on the right. The fits were carried out for the full 0.6-s simulations, which was close to steady state. For a Figure360 author presentation of this figure, see https://doi.org/10.1016/j.bpj.2026.04.015.

Figure360▷

constant was matched to the experimentally measured rate data.[30] This important step allows us to assure the SpringSaLaD volumetric on-rate constants reproduce measured reaction rates. The desired output rate corresponding to an experimentally observed rate constant of 0.27 $\mu M^{-1}s^{-1}$[30] was achieved with a slightly adjusted input rate constant of 0.35 $\mu M^{-1}s^{-1}$, which was then used in subsequent simulations of membrane-bound interactions in Figure 6. In these models, the EGFR membrane anchor site is assigned a diffusion coefficient of 0.01 $\mu m^2/s$ to represent a large transmembrane protein, while the Ras membrane anchor is assigned a diffusion coefficient of 1.0 $\mu m^2/s$ to represent a lipid anchor; all the sites that are dangling in the cytosol volume are given $D_{vol}$ of 1.0 $\mu m^2/s$, but since the binding reaction is not close to diffusion-limited, the precise values are not critical. Consistent with these being reaction-limited on rates, the SpringSaLaD simulation outputs (averages of 100 runs) are very well fitted to reversible mass action kinetic law, as shown in the plots on the right of Figure 6 (relative RMSDs are, respectively, 1.6% and 0.6%). Additionally, as discussed in relation to Figure 5, $k_{on}^{(mem)}$ is insensitive to the level of completion for both cases (see supplemental information spreadsheet Ras_SOS fits for Figure 6), which is consistent with fully reaction-limited mass action kinetics. The 2D on-rate constants ($k_{on}^{(mem)}$) derived from these fits are, respectively, $5.0 \times 10^{-3}$ $\mu m^2$molecules$^{-1}s^{-1}$ and $1.9 \times 10^{-2}$ $\mu m^2$molecules$^{-1}s^{-1}$. Likewise, the affinity of the catalytic site is increased by allosteric site pre-association: $K_D =$ 800 molecules/$\mu m^2$ for the top of Figure 6 and 210 molecules/$\mu m^2$ for the bottom pre-association case. Thus, SOS allosteric site association with Ras is estimated to enhance its catalytic site binding rate and affinity by a factor of ~4—even when SOS is already confined to the membrane through Grb2-mediated association with EGFR.

## A simple ODE model of receptor-mediated signaling

To illustrate the application of our approach, we built a small signaling model based on the values of $k_{on}^{(mem)}$ determined in the previous section. The ODE model was built in VCell,[37,38] and total concentrations of the various species were from measurements on HeLa cells.[34,35] In addition to the two reactions from Figure 6, we also modeled recruitment of Grb_SOS from the cytosol to the membrane by phosphorylated EGFR (EGFRPTyr) and direct stepwise binding of two Ras molecules to Grb_SOS; the model assumes that almost all the cytosolic SOS is prebound to Grb2, which is present in excess.[47] The VCell reaction diagram in Figure 7A displays the connectivity of the network, and Table 3 provides the corresponding mass action rate constants.

We used the data for EGFR measured in HeLa cells[34] to derive an initial surface density for EGFPtyr of 840 molecules/$\mu m^2$, which we took to be the fully activated receptor (we did not attempt to account for kinetics of inactivation, which would lower this number, or for the multiple phosphorylation sites available on the cytoplasmic domain of EGFR, which would raise this number). Figure 7B shows the approach to steady state for this "Full activation" condition. It shows that free EGFPtyr and free Ras are only partially depleted at steady state, while ~90% Grb_SOS is removed from the cytosol to become membrane associated.

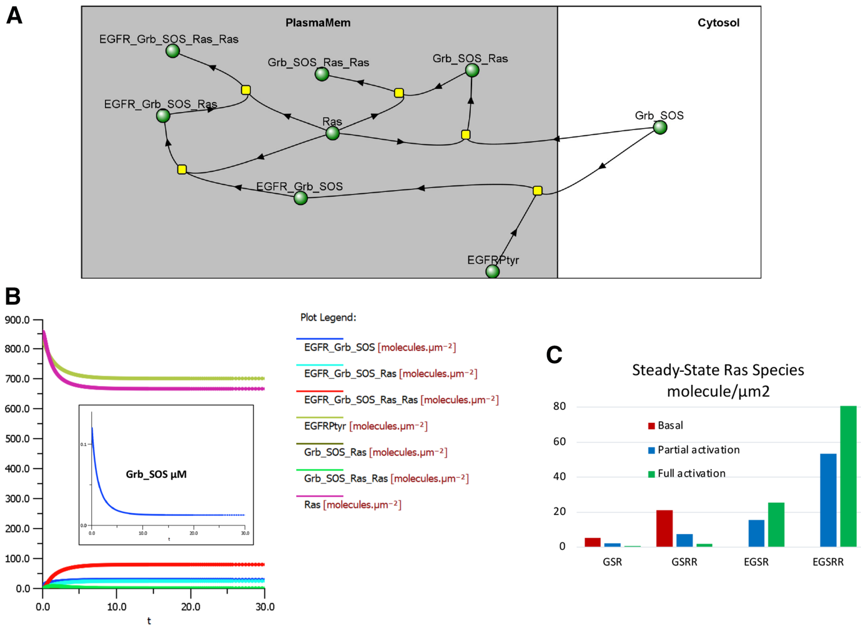


Figure 7. A simple VCell model and simulation results for EGFR signaling to Ras. (A) Reaction network identifying all the variables (see also the corresponding reactions in Table 3.). (B) The kinetics reach steady state within 30 s. The primary plot shows all the membrane species, and the inset shows Grb_SOS in the cytosol. Steady-state surface densities of the 4 different species of 1 or 2 Ras bound to Grb_SOS or EGFRPTyr_Grb_SOS under 3 conditions: "Basal," "Partial activation," and "Full activation" corresponding respectively to 0, 200, and 840 molecules/$\mu m^2$ initial EGFRPTyr.

**Table 3. Rate constants for the VCell model**

| Reaction | $k_{on}$ | $k_{off}$ | Source |
|---|---|---|---|
| EGFRPTyr + Grb_SOS -> EGFR_Grb_SOS | 1.0 μM$^{-1}$s$^{-1}$ | 0.3 s$^{-1}$ | Chook et al.[48] 1996 |
| Grb_SOS + Ras -> Grb_SOS_Ras | 0.27 μM$^{-1}$s$^{-1}$ | 4.0 s$^{-1}$ | Iversen et al.[30] 2014 |
| EGFR_Grb_SOS + Ras -> EGFR_Grb_SOS_Ras | 0.005 μm$^2$molecule$^{-1}$s$^{-1}$ | 4.0 s$^{-1}$ | Iversen et al.[30] 2014 with Figure 6 |
| Ras + EGFR_Grb_SOS_Ras -> EGFR_Grb_SOS_Ras_Ras | 0.019 μm$^2$molecule$^{-1}$s$^{-1}$ | 4.0 s$^{-1}$ | Iversen et al.[30] 2014 with Figure 6 |
| Ras + Grb_SOS_Ras -> Grb_SOS_Ras_Ras | 0.019 μm$^2$molecule$^{-1}$s$^{-1}$ | 4.0 s$^{-1}$ | Iversen et al.[30] 2014 with Figure 6 |

Figure 7C provides a more detailed look at the association of Grb_SOS with Ras. We chose three levels of initial EGFRPTyr, 0, 200 and 840 molecules/μm$^2$, to model, respectively, basal unstimulated cells, partial activation of EGFR, and full activation. The species composed of doubly bound Ras, Grb_SOS_Ras_Ras and EGFR_Grb_SOS_Ras_Ras (GSRR and EGSRR in Figure 5), are always a factor of ~3.5 higher than the corresponding species with one Ras. This is important because the binding of two Ras molecules is required for SOS to optimally express its GEF activity[49]; the second binding event is, of course, significantly enhanced by the proximity effect quantified in Figure 6; this enhancement at the membrane has been reported based on experiments for the binding of Ras to SOS alone.[50] Another conclusion from Figure 7C is that the overall levels of Ras doubly bound to SOS are significantly increased by EGFR activation; this is consistent with the high affinity of the SH2 domains on Grb2 for phosphotyrosine,[48] creating a higher local concentration of SOS near the membrane surface. Indeed, under the "Basal" condition without participation of EGFRPTyr, 80% of the Grb_SOS remains unbound in the cytosol (data not shown but available in the public VCell model "SOS_Recruitment_Ras_Binding").

## DISCUSSION

The kinetics of reactions at membranes have long fascinated biophysicists.[8,10,12,13,17,21,27,40,51] These studies have produced theoretical insights to illuminate how surface-associated reactions have distinct properties compared with reactions occurring in 3D solution. Which of these special properties are most pertinent to any given membrane-bound molecular interaction is difficult to ascertain *a priori*. Furthermore, experiments to measure bimolecular kinetics on membrane surfaces are complex,[6,7] so often only on-rate constants measured in 3D are accessible. Fundamentally, however, the kinetics of key membrane-associated reactions depend on the 2D surface densities and 2D rate constants, not on the bulk cellular concentrations and 3D rate constants. Indeed, because surface to volume ratios of different cell types vary tremendously, volumetric rate constants cannot be readily used to model and simulate cell signaling systems. Furthermore, because bimolecular reactions in 2D may have a special dependence on diffusion, a single mass action rate constant may not be applicable. To address these theoretical and practical problems, we describe a procedure (Figure 1) using experimentally accessible volumetric on-rate constants, $k_{on}^{(vol)}$ within the SpringSaLaD simulation software to estimate the $k_{on}^{(mem)}$, the 2-dimensional rate constant for a membrane-confined bimolecular reaction. Of course, the ideal would be to perform full atomistic molecular dynamics simulation within a realistic lipid bilayer using inter-molecular potentials and force fields.[21,25,26] But deriving typical binding kinetics would require prohibitively expensive multimolecular simulations on the seconds timescale. This is why more highly approximate simulations may be required. In our approach, the potentials are encapsulated in the experimentally determined on-rate constant, and intra- and inter-molecular dynamics are derived from diffusion coefficients using a Langevin dynamics formalism.[28] While the molecular structures are necessarily highly coarse grained, we believe they capture key factors that govern reactivity, namely 2D diffusion, proximity of binding sites to the membrane, and the steric environment of the binding sites.

To validate the method of Figure 1, we applied it to the dimerization of a single binding site tethered to a surface through a 5-nm stiff linker, where the membrane anchor acts as a pivot (Table 1). For the situation where the reaction is rate limiting, this system can be solved theoretically (see Supporting Text I); gratifyingly, $k_{on}^{(mem)}$ determined by our method is well reproduced by this solution. Interestingly, for these cases, $k_{on}^{(mem)}$ is well approximated by $k_{on}^{(h)} = k_{on}^{(vol)}/h/602.2$ (μm$^2$molecules$^{-1}$s$^{-1}$), where $h$ is the distance of the binding site from the membrane anchor (in μm) and 602.2 is a unit conversion factor. The parameter $h$ has also been referred to as the "confinement length,"[21] defining a thin volume above the membrane that concentrates the binding sites and directly produces the relationship between $k_{on}^{(h)}$ and $k_{on}^{(vol)}$.

While mass action kinetics are generally applicable for both encounter-limited and reaction-limited kinetics in 3D solution (but see Keizer[14] and Collins and Kimball[15]), it has long been appreciated that the situation may be more complex for 2D kinetics.[8,10,12,13,19,40] This is demonstrated by the results in Table 1 for situations where the diffusion coefficient of the anchor is slow, but the volumetric on-rate constant is fast (rows 5 and 7). For these cases, different estimates of $k_{on}^{(mem)}$ are obtained at different initial surface

density—clearly incompatible with the reaction-limited kinetics. Indeed, the third panel of Figure 2 shows that the SpringSaLaD kinetic data are only approximately fit by a mass action rate law; and Figure 2D displays a terrible fit. A video of one trajectory (Video S1) nicely illustrates how the initial rate is fast, while the binding sites are within "reach"[27] but fall off as binding sites are left orphaned outside the reach of the remaining slowly diffusing monomers. Because of this, reaction-limited kinetics apply better to high initial concentrations and to shorter time durations, before depletion zones develop. To address the reliability of our method theoretically, even for cases that are influenced by diffusion, we solved the Smoluchowski model[14] in 2D using VCell (Supporting Text II) to generate the relations plotted in Supporting Text II Figures S7 and S8 and Figure 4 in the main text for the time dependence of the diffusion-limited reaction coefficient. This allowed us to implement a pipeline for computing a weighted average of the on-rate coefficient, $\bar{k}(t_c)$ over time, for any intrinsic on-rate constant, $k_0$, membrane diffusion coefficient, initial surface density, and desired level of completion. We used this to show that for 19 idealized scenarios, a good correlation between $\bar{k}(t_c)$ and $k_{on}^{(mem)}$ was displayed (Figure 3).

Thus, as opposed to reaction-limited kinetics, where $k_{on}^{(mem)}$ can be considered a fixed constant for a broad range of conditions, when the system is "diffusion influenced," both the initial surface densities and the desired level of completion will strongly determine $k_{on}^{(mem)}$ (Table 1, rows 5 versus 7; Figure 5). As the system goes further to completion, with fewer of the remaining reactants within reach of the tethers, the system will transition to fully diffusion limited with rate constants much lower than reaction limited; under these conditions, the system will no longer be described by an invariant $k_{on}^{(mem)}$. Still, if the modeler can analyze the overall reaction network to assess, at least approximately, what the initial concentrations are and how far to completion the membrane-tethered reaction is likely to go, it may be possible to approximate the levels of reactant and product with a single constant $k_{on}^{(mem)}$ parameter and a mass action ODE; this is especially true for long times, when diffusion is limiting, because of the very slow decline in $k_D(t)$ (Figure 4 and Supporting Text II Figure S8). The best metric to assess this is the relative RMSD for the fit, which measures how significantly the SpringSaLaD mean trajectory deviates from the mass action fitted curve.

To explore how other molecular structural features might affect dimerization of the monomers tethered to the membrane, we looked at three additional idealized systems in Table 2. In all these, $h$ was 20.5 nm (as opposed to 5.5 nm in Table 1). As expected, the longer confinement length decreased the estimated $k_{on}^{(mem)}$ by a factor of $\sim$4. The insertion of steric sites between the anchor and the binding site or allowing for flexibility of the linker region has minor effects on $k_{on}^{(mem)}$, which is relatively well approximated by $k_{on}^{(h)}$.

For membrane binding of real biological molecules, the average location of binding sites relative to the membrane surface (i.e., $h$) will generally be not well defined, since it is influenced by the steric effects and the variable flexibility of neighboring protein domains. Also, the two binding sites may be parts of very different structures with different distances from the membrane surface. In situations like this, our approach has the potential to provide good estimates of rate constants that can be applied to larger cell signaling systems. Indeed, there may be direct insights that can be realized just by considering the structural details of the interacting membrane molecules. We have illustrated this in relation to adaptor-mediated protein kinase receptor signaling mechanisms, specifically for the interaction of the GEF SOS with its effector Ras (Figure 6). This system has been the subject of many modeling studies[46,52–58] because of the importance of Ras as a key oncogene.[59] It has been shown that direct catalysis by the SOS catalytic domain of Ras conversion from the GDP to the GTP states is relatively slow. However, binding of Ras to SOS at a site that is not catalytic (termed the "allosteric" site on SOS) significantly accelerates the catalytic activity,[49,50] where the catalysis becomes processive.[30,44,46] Here, we confine ourselves to the membrane recruitment of SOS mediated by active EGFR and ask how the specific details of membrane association might affect SOS activity. The fit of the SpringSaLaD simulations to a mass action rate law is excellent in both scenarios of Figure 6, indicating that this system is reaction limited, and mass action is applicable to a range of conditions. The results in Figure 6 suggest that at least part of the mechanism for allosteric activation is due to the close proximity of the SOS catalytic site to the membrane once it is bound to Ras at its allosteric site. Even though SOS is already localized to the membrane by initially binding to EGFR via Grb2 in our computational experiment, pre-binding of the SOS allosteric site to Ras brings it even closer to the membrane. Of course, there could be additional effects such as a direct allosteric enhancement through a conformational change or release of self-inhibition,[30,44,46] but here we focus on the significance of constricting the binding zone through membrane tethers of varying length and flexibility.

To illustrate the application of our approach to cell signaling, we constructed a simple model of EGFR signaling to Ras via SOS in HeLa cells (Figure 7). The results show that the proximity effect established in Figure 6 for the binding of SOS to the second Ras molecule results in the dominance of doubly bound Ras species. This no doubt contributes to the efficiency of the GEF activity associated with SOS. Our simple model also demonstrates the effectiveness of active EGFR in boosting the recruitment of Grb_SOS to the membrane where it presents an effectively high local concentration to Ras. The model, which

is available in the VCell database, can be further explored for the effect of varying Ras or SOS concentrations or the behavior of different cell types with different levels of the key signaling models; it could also be used as a starting point for more elaborate models that incorporate more detailed mechanisms and additional downstream signaling events.

In conclusion, we showed how to estimate a 2D rate constant for a mass action rate equation for membrane-tethered molecules, even when diffusion is limiting. While there are many approximations associated with our approach, we believe it represents a significant improvement over the typical simplifications that are used to parameterize large reaction network models. In ODE models, diffusion is assumed to be fast on the timescale of reaction rates; however, our approach is necessary to capture the special local membrane effects that allow conversion of a bulk measurement of the on-rate constant to $k_{on}^{(mem)}$. In cell-scale PDE (or spatial stochastic) models, where diffusion is considered explicitly, the membrane molecules are not resolved, and the distances are very large on the scale of molecular dimensions. Using $k_{on}^{(mem)}$ will allow these models to capture the role of molecular structural effects and the influence of membrane diffusion.

## DATA AVAILABILITY

All the data used to produce the results in Tables 1 and 2 and in Figures 2, 3, 4, 5, and 6 are available as Excel spreadsheets in the Supporting Data and Information deposited with this paper. The SpringSaLaD and VCell software used to build the models can be downloaded at vcell.org. The results displayed in Figure 7 are from a public VCell model "SOS_Recruitment_Ras_Binding," which is available on servers accessible through the VCell software.

## ACKNOWLEDGMENTS

This work was supported by NIH grants R24 GM137787 and R01 GM132859. We are pleased to acknowledge the advice of Aniruddha Chattaraj with some of the data analysis.

## AUTHOR CONTRIBUTIONS

K.J.P. created the computational models, performed the simulations, analyzed the data and wrote the paper; B.M.S. performed the calculations in Supporting Text I and II and wrote the paper; L.M.L. conceived the research, analyzed the data, and wrote the paper.

## DECLARATION OF INTERESTS

The authors declare no competing interests.

## SUPPLEMENTAL INFORMATION

Supplemental information can be found online at https://doi.org/10.1016/j.bpj.2026.04.015.

## REFERENCES


1. Slepchenko, B. M., J. C. Schaff, …, L. M. Loew. 2003. Quantitative cell biology with the Virtual Cell. *Trends Cell Biol.* 13:570–576. http://www.ncbi.nlm.nih.gov/entrez/query.fcgi?cmd=Retrieve&db=PubMed&dopt=Citation&list_uids=14573350.
2. Eungdamrong, N. J., and R. Iyengar. 2004. Modeling Cell Signaling Networks. *Biol. Cell.* 96:355–362. https://doi.org/10.1016/j.biolcel.2004.03.004. https://www.sciencedirect.com/science/article/pii/S0248490004000772.
3. Myers, P. J., S. H. Lee, and M. J. Lazzara. 2021. Mechanistic and data-driven models of cell signaling: tools for fundamental discovery and rational design of therapy. *Curr. Opin. Syst. Biol.* 28:100349. https://doi.org/10.1016/j.coisb.2021.05.010.
4. Banga, J. R., and A. F. Villaverde. 2025. Mechanistic dynamic modelling of biological systems: The road ahead. *Curr. Opin. Syst. Biol.* 42:100553. https://doi.org/10.1016/j.coisb.2025.100553. https://www.sciencedirect.com/science/article/pii/S2452310025000137.
5. Shinobu, A., A. Nagasato-Ichikawa, and M. Okada. 2026. Network structures and parameters in multiscale modeling in ErbB signaling networks. *Curr. Opin. Cell Biol.* 98:102603. https://doi.org/10.1016/j.ceb.2025.102603. https://www.sciencedirect.com/science/article/pii/S0955067425001413.
6. Groves, J. T., and M. L. Dustin. 2003. Supported planar bilayers in studies on immune cell adhesion and communication. *J. Immunol. Methods.* 278:19–32. https://doi.org/10.1016/S0022-1759(03)00193-5. https://www.sciencedirect.com/science/article/pii/S0022175903001935.
7. Gavutis, M., S. Lata, and J. Piehler. 2006. Probing 2-dimensional protein–protein interactions on model membranes. *Nat. Protoc.* 1:2091–2103. https://doi.org/10.1038/nprot.2006.270.
8. Adam, G., and M. Delbrück. 1968. Reduction of Dimensionality in Biological Diffusion. *In* Structural Chemistry and Molecular Biology. A. Rich and N. Davidson, eds W. H. Freeman and Co., San Francisco, pp. 198–215.
9. Pólya, G. 1921. Über eine Aufgabe der Wahrscheinlichkeitsrechnung betreffend die Irrfahrt im Straßennetz. *Math. Ann.* 84:149–160. https://doi.org/10.1007/BF01458701.de.
10. Torney, D. C., H. M. McConnell, and G. R. Porter. 1983. Diffusion-limited reaction rate theory for two-dimensional systems. *Proceedings of the Royal Society of London. A. Mathematical and Physical Sciences.* 387:147–170. https://doi.org/10.1098/rspa.1983.0055. https://royalsocietypublishing.org/doi/abs/10.1098/rspa.1983.0055.
11. Koudriavtsev, A. B., R. F. Jameson, W. Linert; SpringerLink. 2001. The Law of Mass Action. Springer, Berlin Heidelberg.
12. Yogurtcu, O. N., and M. E. Johnson. 2015. Theory of bi-molecular association dynamics in 2D for accurate model and experimental parameterization of binding rates. *J. Chem. Phys.* 143:084117. https://doi.org/10.1063/1.4929390.
13. Axelrod, D., and M. D. Wang. 1994. Reduction-of-dimensionality kinetics at reaction-limited cell surface receptors. *Biophys. J.* 66:588–600. https://doi.org/10.1016/s0006-3495(94)80834-3. https://www.ncbi.nlm.nih.gov/pubmed/8011892.
14. Keizer, J. 1987. Diffusion effects on rapid bimolecular chemical reactions. *Chem. Rev.* 87:167–180. https://doi.org/10.1021/cr00077a009.
15. Collins, F. C., and G. E. Kimball. 1949. Diffusion-controlled reaction rates. *J. Colloid Sci.* 4:425–437. https://doi.org/10.1016/0095-8522(49)90023-9. https://www.sciencedirect.com/science/article/pii/0095852249900239.
16. Szabo, A. 1989. Theory of diffusion-influenced fluorescence quenching. *J. Phys. Chem.* 93:6929–6939. https://doi.org/10.1021/j100356a011.
17. Haugh, J. M. 2002. A Unified Model for Signal Transduction Reactions in Cellular Membranes. *Biophys. J.* 82:591–604. http://www.biophysj.org/cgi/content/abstract/82/2/591.
18. Windisch, B., D. Bray, and T. Duke. 2006. Balls and Chains—A Mesoscopic Approach to Tethered Protein Domains. *Biophys. J.* 91:2383–2392. https://doi.org/10.1529/biophysj.105.078543. https://www.sciencedirect.com/science/article/pii/S0006349506719531.

19. Bell, G. I. 1978. Models for the Specific Adhesion of Cells to Cells. *Science.* 200:618–627. https://doi.org/10.1126/science.347575. https://www.science.org/doi/abs/10.1126/science.347575.

20. Bell, G. I., M. Dembo, and P. Bongrand. 1984. Cell adhesion. Competition between nonspecific repulsion and specific bonding. *Biophys. J.* 45:1051–1064. https://doi.org/10.1016/S0006-3495(84)84252-6. https://www.ncbi.nlm.nih.gov/pubmed/6743742.

21. Wu, Y., J. Vendome, …, B. Honig. 2011. Transforming binding affinities from three dimensions to two with application to cadherin clustering. *Nature.* 475:510–513. https://doi.org/10.1038/nature10183.

22. Weikl, T. R., J. Hu, …, R. Lipowsky. 2016. Binding equilibrium and kinetics of membrane-anchored receptors and ligands in cell adhesion: Insights from computational model systems and theory. *Cell Adh. Migr.* 10:576–589. https://doi.org/10.1080/19336918.2016.1180487. https://www.ncbi.nlm.nih.gov/pubmed/27294442.

23. Xu, G. K., J. Hu, …, T. R. Weikl. 2015. Binding constants of membrane-anchored receptors and ligands: A general theory corroborated by Monte Carlo simulations. *J. Chem. Phys.* 143:243136. https://doi.org/10.1063/1.4936134. https://www.ncbi.nlm.nih.gov/pubmed/26723621.

24. Jhaveri, A., S. Chhibber, …, M. E. Johnson. 2025. Binding affinities for 2D protein dimerization benefit from enthalpic stabilization. *bioRxiv.* https://doi.org/10.1101/2025.01.16.633485.

25. Xie, Z.-R., J. Chen, and Y. Wu. 2014. Linking 3D and 2D binding kinetics of membrane proteins by multiscale simulations. *Protein Sci.* 23:1789–1799. https://doi.org/10.1002/pro.2574. https://onlinelibrary.wiley.com/doi/abs/10.1002/pro.2574.

26. Hu, J., G.-K. Xu, …, T. R. Weikl. 2015. Binding kinetics of membrane-anchored receptors and ligands: Molecular dynamics simulations and theory. *J. Chem. Phys.* 143:243137. https://doi.org/10.1063/1.4936135.

27. Zhang, Y., L. Clemens, …, S. A. Isaacson. 2019. The Influence of Molecular Reach and Diffusivity on the Efficacy of Membrane-Confined Reactions. *Biophys. J.* 117:1189–1201. https://doi.org/10.1016/j.bpj.2019.08.023.

28. Michalski, P. J., and L. M. Loew. 2016. SpringSaLaD: A Spatial, Particle-Based Biochemical Simulation Platform with Excluded Volume. *Biophys. J.* 110:523–529. https://doi.org/10.1016/j.bpj.2015.12.026. http://www.ncbi.nlm.nih.gov/pubmed/26840718.

29. Chattaraj, A., M. Youngstrom, and L. M. Loew. 2018. The interplay of structural and cellular biophysics controls clustering of multivalent molecules. *bioRxiv.* https://doi.org/10.1101/373084. https://www.biorxiv.org/content/biorxiv/early/2018/12/26/373084.full.pdf.

30. Iversen, L., H.-L. Tu, …, J. T. Groves. 2014. Ras activation by SOS: Allosteric regulation by altered fluctuation dynamics. *Science.* 345:50–54. https://doi.org/10.1126/science.1250373. https://science.sciencemag.org/content/sci/345/6192/50.full.pdf.

31. Abramson, J., J. Adler, …, J. M. Jumper. 2024. Accurate structure prediction of biomolecular interactions with AlphaFold 3. *Nature.* 630:493–500. https://doi.org/10.1038/s41586-024-07487-w.

32. Jumper, J., R. Evans, …, D. Hassabis. 2021. Highly accurate protein structure prediction with AlphaFold. *Nature.* 596:583–589. https://doi.org/10.1038/s41586-021-03819-2.

33. Masison, J., P. J. Michalski, …, A. D. Schuyler. 2018. mol2sphere: spherical decomposition of multi-domain molecules for visualization and coarse grained spatial modeling. *Bioinformatics.* 34:3948–3950. https://doi.org/10.1093/bioinformatics/bty487. https://www.ncbi.nlm.nih.gov/pubmed/29931043.

34. Kamioka, Y., S. Yasuda, …, M. Matsuda. 2010. Multiple decisive phosphorylation sites for the negative feedback regulation of SOS1 via ERK. *J. Biol. Chem.* 285:33540–33548. https://doi.org/10.1074/jbc.M110.135517. https://www.ncbi.nlm.nih.gov/pubmed/20724475.

35. Fujioka, A., K. Terai, …, M. Matsuda. 2006. Dynamics of the Ras/ERK MAPK Cascade as Monitored by Fluorescent Probes. *J. Biol. Chem.* 281:8917–8926. https://doi.org/10.1074/jbc.M509344200. https://www.sciencedirect.com/science/article/pii/S0021925819565978.

36. Hoops, S., S. Sahle, …, U. Kummer. 2006. COPASI: a COmplex PAthway SImulator. *Bioinformatics.* 22:3067–3074. https://doi.org/10.1093/bioinformatics/btl485.

37. Schaff, J., C. C. Fink, …, L. M. Loew. 1997. A general computational framework for modeling cellular structure and function. *Biophys. J.* 73:1135–1146. https://doi.org/10.1016/S0006-3495(97)78146-3. http://www.ncbi.nlm.nih.gov/entrez/query.fcgi?cmd=Retrieve&db=PubMed&dopt=Citation&list_uids=9284281.

38. Cowan, A. E., I. I. Moraru, …, L. M. Loew. 2012. Spatial modeling of cell signaling networks. *Methods Cell Biol.* 110:195–221. https://doi.org/10.1016/B978-0-12-388403-9.00008-4. http://www.ncbi.nlm.nih.gov/pubmed/22482950.

39. Kopelman, R. 1986. Rate processes on fractals: Theory, simulations, and experiments. *J. Stat. Phys.* 42:185–200. https://doi.org/10.1007/BF01010846. https://www.scopus.com/inward/record.uri?eid=2-s2.0-30244438013&doi=10.1007%2fBF01010846&partnerID=40&md5=8220d250ae1bd74cb14dad318176663e.

40. Berry, H. 2002. Monte Carlo Simulations of Enzyme Reactions in Two Dimensions: Fractal Kinetics and Spatial Segregation. *Biophys. J.* 83:1891–1901. https://doi.org/10.1016/S0006-3495(02)73953-2. https://www.sciencedirect.com/science/article/pii/S0006349502739532.

41. Ovchinnikov, A. A., and Y. B. Zeldovich. 1978. Role of density fluctuations in bimolecular reaction kinetics. *Chem. Phys.* 28:215–218.

42. Toussaint, D., and F. Wilczek. 1983. Particle–antiparticle annihilation in diffusive motion. *J. Chem. Phys.* 78:2642–2647. https://doi.org/10.1063/1.445022.

43. Tachiya, M. 1983. Theory of diffusion-controlled reactions: Formulation of the bulk reaction rate in terms of the pair probability. *Radiat. Phys. Chem.* 21:167–175. https://doi.org/10.1016/0146-5724(83)90143-7. https://www.sciencedirect.com/science/article/pii/0146572483901437.

44. Bandaru, P., Y. Kondo, and J. Kuriyan. 2019. The Interdependent Activation of Son-of-Sevenless and Ras. *Cold Spring Harb. Perspect. Med.* 9:a031534. https://doi.org/10.1101/cshperspect.a031534. https://www.ncbi.nlm.nih.gov/pubmed/29610148.

45. Jorissen, R. N., F. Walker, …, A. W. Burgess. 2003. Epidermal growth factor receptor: mechanisms of activation and signalling. *Exp. Cell Res.* 284:31–53. https://doi.org/10.1016/S0014-4827(02)00098-8. http://www.sciencedirect.com/science/article/pii/S0014482702000988.

46. Ren, H., A. A. Lee, …, J. T. Groves. 2024. Positive feedback in Ras activation by full-length SOS arises from autoinhibition release mechanism. *Biophys. J.* 123:3295–3303. https://doi.org/10.1016/j.bpj.2024.07.014.

47. Giubellino, A., T. R. Burke, and D. P. Bottaro. 2008. Grb2 signaling in cell motility and cancer. *Expert Opin. Ther. Targets.* 12:1021–1033. https://doi.org/10.1517/14728222.12.8.1021.

48. Chook, Y. M., G. D. Gish, …, T. Pawson. 1996. The Grb2-mSos1 complex binds phosphopeptides with higher affinity than Grb2. *J. Biol. Chem.* 271:30472–30478. https://doi.org/10.1074/jbc.271.48.30472. https://www.ncbi.nlm.nih.gov/pubmed/8940013.

49. Margarit, S. M., H. Sondermann, …, J. Kuriyan. 2003. Structural Evidence for Feedback Activation by Ras·GTP of the Ras-Specific Nucleotide Exchange Factor SOS. *Cell.* 112:685–695. https://doi.org/10.1016/S0092-8674(03)00149-1. https://www.sciencedirect.com/science/article/pii/S0092867403001491.

50. Gureasko, J., W. J. Galush, …, J. Kuriyan. 2008. Membrane-dependent signal integration by the Ras activator Son of sevenless. *Nat. Struct. Mol. Biol.* 15:452–461. https://doi.org/10.1038/nsmb.1418.

51. McCloskey, M. A., and M. M. Poo. 1986. Rates of Membrane-associated Reactions: Reduction of Dimentionality Revisited. *J. Cell Biol.* 102:88–96.

52. Eungdamrong, N. J., and R. Iyengar. 2007. Compartment-specific feedback loop and regulated trafficking can result in sustained activation of Ras at the Golgi. *Biophys. J.* 92:808–815. http://www.ncbi.nlm.nih.gov/entrez/query.fcgi?cmd=Retrieve&db=PubMed&dopt=Citation&list_uids=17098795.

53. Tian, T., A. Harding, …, J. F. Hancock. 2007. Plasma membrane nanoswitches generate high-fidelity Ras signal transduction. *Nat. Cell Biol.* 9:905–914. https://doi.org/10.1038/ncb1615http://www.nature.com/ncb/journal/v9/n8/suppinfo/ncb1615_S1.html.

54. Das, J., M. Ho, …, J. P. Roose. 2009. Digital Signaling and Hysteresis Characterize Ras Activation in Lymphoid Cells. *Cell.* 136:337–351. https://doi.org/10.1016/j.cell.2008.11.051. https://www.sciencedirect.com/science/article/pii/S0092867408016310.

55. Coyle, S. M., and W. A. Lim. 2016. Mapping the functional versatility and fragility of Ras GTPase signaling circuits through in vitro network reconstitution. *eLife.* 5:e12435. https://doi.org/10.7554/eLife.12435.

56. Erickson, K. E., O. S. Rukhlenko, …, B. N. Kholodenko. 2019. New insights into RAS biology reinvigorate interest in mathematical modeling of RAS signaling. *Semin. Cancer Biol.* 54:162–173. https://doi.org/10.1016/j.semcancer.2018.02.008. https://www.sciencedirect.com/science/article/pii/S1044579X17302857.

57. Huang, W. Y. C., S. Alvarez, …, J. T. Groves. 2019. A molecular assembly phase transition and kinetic proofreading modulate Ras activation by SOS. *Science.* 363:1098–1103. https://doi.org/10.1126/science.aau5721. http://science.sciencemag.org/content/sci/363/6431/1098.full.pdf.

58. Huang, W. Y. C., S. Alvarez, …, J. T. Groves. 2021. Relating cellular signaling timescales to single-molecule kinetics: A first-passage time analysis of Ras activation by SOS. *Proc. Natl. Acad. Sci. USA.* 118:e2103598118. https://doi.org/10.1073/pnas.2103598118. https://www.pnas.org/content/pnas/118/45/e2103598118.full.pdf.

59. Pylayeva-Gupta, Y., E. Grabocka, and D. Bar-Sagi. 2011. RAS oncogenes: weaving a tumorigenic web. *Nat. Rev. Cancer.* 11:761–774. https://doi.org/10.1038/nrc3106.

Biophysical Journal, Volume *125*

## Supplemental information

# Mesoscale simulations of membrane-tethered reactions to parameter-ize cell-scale models of signaling

**Kelvin J. Peterson, Boris M. Slepchenko, and Leslie M. Loew**

# Supporting Text

## I. Derivation of an equivalent 2D dimerization rate constant for tethered binders in the limit of $D_{mem} \ll D_{vol}$ .

Here we derive $k_{on}^{(mem)}$ analytically for conditions pertaining to the first row in Table 1 of the main text. Our approach is conceptually similar to the one previously used in modeling the binding of bivalent haptens to an antibody (Dembo and Goldstein, 1978).

As in SpringSaLad (Michalski and Loew, 2016), we model a binder as a ball of diameter $d$. A link of length $L$ tethers the ball's center to a membrane-bound anchor. Because the anchor diffuses in the membrane much slower than the binder in the cytosol, the link restricts movements of the binder, so that at any given time, the binder is generally confined to a hemisphere, centered at a current position of the anchor, with the radius $R = L + \frac{d}{2}$ (in the main text, this parameter is termed $h$). The simulation results, which we seek to understand, were obtained for $d < \frac{1}{5}L$, so that the excluded volume effects due to the binder's finite size can be ignored. We disregard possible effects on $k_{on}^{(mem)}$ of the collisions or entanglement of the tethers, given that the links in SpringSaLad are not affected by each other.

Importantly, we assume that during dimerization, the monomers remain uniformly distributed. A formal analysis of the conditions underlying this assumption is provided in part II below.

With the assumptions outlined above, the effective 2D rate constant $k_{on}^{(mem)}$ for the dimerization of monomers tethered to the membrane can be obtained analytically in terms of the dimerization rate constant $k_{on}^{(vol)}$ of free binders.

Two binders can collide if the distance between their anchors does not exceed $2R$. As mentioned earlier, the anchors can be viewed as "well-mixed" with some surface density $\sigma$. Without loss of generality, we may assume that the anchor of one of the monomers is immobile (in what follows, we will call such a monomer 'a given monomer'). The number of binding partners of this monomer is then

$$n = 4\pi R^2 \sigma. \qquad \text{Eq (S1)}$$

Our goal is to derive the rate of dimerization of a given monomer.

We first consider cases where the links between a binder and its anchor are stiff. In such cases, given that $D_{mem} \ll D_{vol}$, the binder may be thought of as uniformly distributed at any time within a hemispherical shell centered at a current position of its anchor. The shell has the outer radius $R$, the inner radius $r = L - \frac{d}{2}$, and the volume $v_{shell} = \frac{2\pi R^3}{3}\left(1 - \left(\frac{r}{R}\right)^3\right)$ , so the binder 'concentration' (more precisely, the binder probability density function) is $\frac{1}{v_{shell}} = [\frac{2\pi R^3}{3}\left(1 - \left(\frac{r}{R}\right)^3\right)]^{-1}$.

The dimerization of a given monomer with a binding partner requires that their binders collide, which occurs at the intersection of their shells (the shared space in Figure S1). The collision probability for the binder of the given monomer, distributed within the shell shaded in Figure S1,

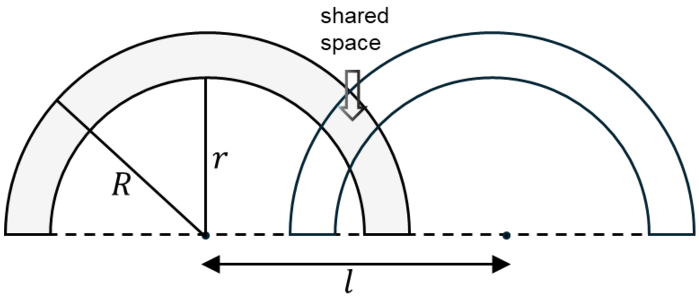


**Figure S1. Cross-section of overlapping hemispherical shells.**
The shells cover binder positions of two monomers with anchors separated by distance $l$.

$p_{coll} = \frac{v_{shared}}{v_{shell}}$, where $v_{shared}$ is the volume of the shared space. As $v_{shared}$ depends on distance $l$ separating the anchors, so does the collision probability. Because the anchors are well-mixed, the mobile anchor of the binding partner (the center of the unshaded shell in Figure S1) is uniformly distributed within the circle of radius $2R$ with the center at the fixed anchor. The average collision probability is found by integrating of $p_{coll}(l) = \frac{v_{shared}(l)}{v_{shell}}$ over this circle:

$$\bar{p}_{coll} = \frac{1}{\pi(2R)^2}\int_0^{2\pi} d\varphi \int_0^{2R} p_{coll}(l) l dl = \frac{2}{(2R)^2}\int_0^{2R} p_{coll}(l) l dl. \qquad \text{Eq (S2)}$$

Then the rate of dimerization of the given monomer with a single binding partner is the product of the rate constant of dimerization of free binders $k_{on}^{(vol)}$ and the 'concentration' of the single binder $\frac{1}{v_{shell}}$ modified by the average collision probability: $\bar{p}_{coll}\frac{k_{on}^{(vol)}}{v_{shell}}$.

We now recall that the given monomer has $n$ binding partners defined by Eq (S1). For anchor densities that are not too high, so that the events of three-binder collisions can be ignored, the rate of dimerization of a given monomer with any of the available binding partners is simply $n\bar{p}_{coll}\frac{k_{on}^{(vol)}}{v_{shell}} = \left(\frac{4\pi R^2 \bar{p}_{coll} k_{on}^{(vol)}}{v_{shell}}\right)\sigma$, where the expression in the parenthesis is the sought formula for the equivalent $k_{on}^{(mem)}$:

$$k_{on}^{(mem)} = \frac{4\pi R^2 \bar{p}_{coll} k_{on}^{(vol)}}{v_{shell}} = \frac{4\pi R^2}{v_{shell}^2} k_{on}^{(vol)} \int_0^1 v_{shared}(\rho) 2\rho d\rho, \qquad \text{Eq (S3)}$$

where we introduced the dimensionless variable $\rho = \frac{l}{2R}$.

We now turn to calculations of $v_{shared}(\rho)$. Introducing for brevity the notation $a = \frac{r}{R}$, we observe that for any $a, \rho \leq 1$, the space shared by two intersecting spherical shells is a combination of spherical caps. It is therefore convenient to introduce a function,

$$f(x) = 1 - \frac{3}{2}x + \frac{1}{2}x^3, \qquad \text{Eq (S4)}$$

which for $x = \cos\theta$ yields the volume fraction of a hemisphere that is occupied by a spherical cap with polar angle $\theta$ (Figure S2).

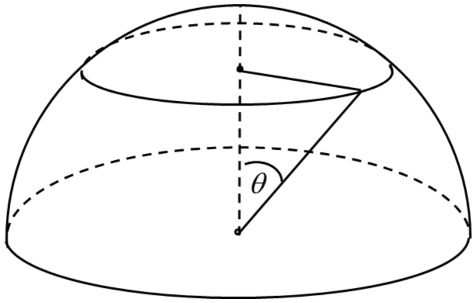


**Figure S2. A spherical cap defined by a polar angle $\theta$ .**
The cap is shown as a part of the corresponding hemisphere.

The structure of $v_{shared}(\rho)$ depends on the value of $a$. For $a \geq \frac{1}{3}$, the derivation based on stereometric considerations yields the following dependence:

$$\frac{v_{shared}(\rho)}{(2\pi R^3/3)} = \begin{cases} f(\rho) - a^3 f\left(-\frac{\rho}{a}\right), & \text{for } \rho \in \left[0,\ \frac{1}{2}(1-a)\right] \\ f(\rho) + a^3 f\left(\frac{\rho}{a}\right) - f\left(\frac{1-a^2}{4\rho} + \rho\right) - a^3 f\left(\frac{a^2-1}{4\rho a} + \frac{\rho}{a}\right), & \text{for } \rho \in \left[\frac{1}{2}(1-a),\ a\right] \\ f(\rho) - f\left(\frac{1-a^2}{4\rho} + \rho\right) - a^3 f\left(\frac{a^2-1}{4\rho a} + \frac{\rho}{a}\right), & \text{for } \rho \in \left[a, \frac{1}{2}(1+a)\right] \\ f(\rho), & \text{for } \rho \in \left[\frac{1}{2}(1+a), 1\right] \end{cases} \quad \text{. Eq (S5)}$$

Eq (S5) applies to the stiff-tether cases discussed in the main text. Indeed, for $L = 5$ nm and $d = 1$ nm,the outer and inner shell radii are $R = 5.5$ nm, $r = 4.5$ nm, resulting in $a = \frac{r}{R} = \frac{9}{11} > \frac{1}{3}$, and the monomers with larger $L$ and same $d$ are characterized by even larger $a$. Figure S3 illustrates $\frac{v_{shared}}{\frac{2\pi R^3}{3}}$ as a function of $\rho$ for $a = \frac{9}{11}$.

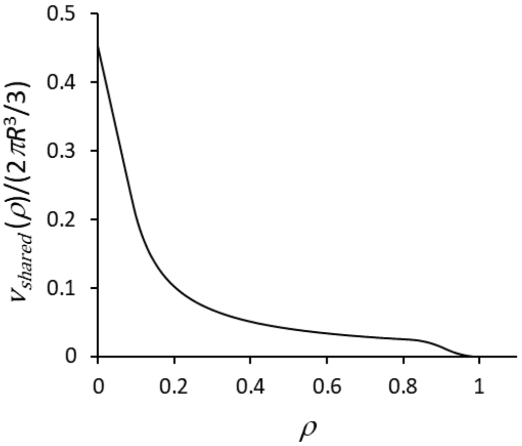


**Figure S3. Volume of intersection of two identical shells as a function of distance between their centers.**
The volume of the shared space, normalized to the volume of the hemisphere, is shown as a function of the normalized distance $\rho = \frac{l}{2R}$; $L = 5$ nm, $d = 1$ nm.

The integral in Eq(S3) is readily evaluated,

$$\int_0^1 v_{shared}(\rho)2\rho d\rho = \frac{2\pi R^3}{3}\{\frac{1}{5}(1+a^5)+\frac{a}{8}(1-a^2)^2-\frac{1}{80}((1+a)^5-(1-a)^5)\}. \qquad \text{Eq (S6)}$$

Substituting Eq (S6) into Eq (S3) and noting that $v_{shell} = \frac{2\pi R^3}{3}\left(1-\left(\frac{r}{R}\right)^3\right) = \frac{2\pi R^3}{3}(1-a^3)$, we find

$$k_{on}^{(mem)} = \phi(a)\frac{k_{on}^{(vol)}}{R}, \qquad \text{Eq (S7a)}$$

where

$$\phi(a) = \frac{\frac{6}{5}(1+a^5)+\frac{3a}{4}(1-a^2)^2-\frac{3}{40}((1+a)^5-(1-a)^5)}{(1-a^3)^2}. \qquad \text{Eq (S7b)}$$

In the terms of the main text, $\frac{k_{on}^{(vol)}}{R} = \frac{k_{on}^{(vol)}}{h} = k_{on}^{(h)}$, and from Eqs (S7a), $\phi(a) = \frac{k_{on}^{(mem)}}{k_{on}^{(h)}}$. For stiff tethers with length $L = 5$ nm and a binder's diameter $d = 1$ nm, $a = \frac{9}{11}$ and $\frac{k_{on}^{(mem)}}{k_{on}^{(h)}} = 1.06$. This is close to $\frac{k_{on}^{(mem)}}{k_{on}^{(h)}} = 0.967$ from the first row of Table 1 (main text). Thus, the analytical solution validates the simulation results obtained with SpringSaLaD.

Note that the ratio $\frac{k_{on}^{(mem)}}{k_{on}^{(h)}}$ are not particularly sensitive to tether lengths $L$ in the limit $D_{mem} \ll D_{vol}$ (Figure S4A), and it is nearly the same as in the case of $D_{mem} = D_{vol}$ (second row of Table 1). This appears counterintuitive, given that for $D_{mem} \ll D_{vol}$, the tethering causes the collision probability to decrease with $L$, whereas for equal diffusivities, the binders are effectively unconstrained by their anchors in their movements within the layer of height $L + \frac{d}{2}$, adjacent to the membrane.

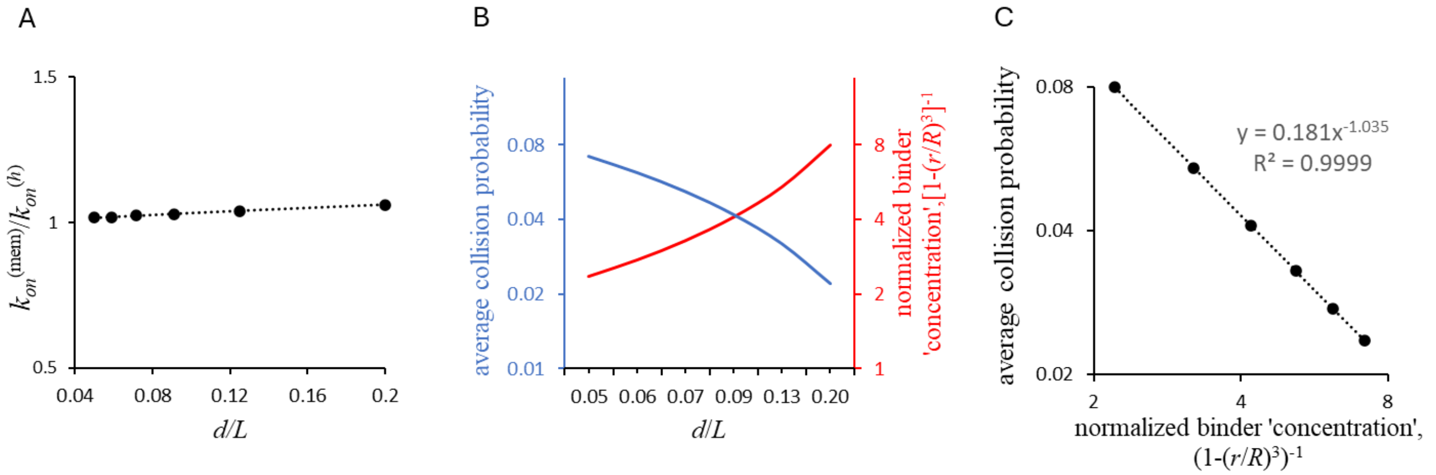


**Figure S4. Ratio $k_{on}^{(mem)}/k_{on}^{(h)}$ has low sensitivity to tether length $L$.**
(A) The ratio $k_{on}^{(mem)}/k_{on}^{(h)}$ as a function of the ratio of binder diameter $d$ and tether length $L$; $d = 1$ nm. (B) Interplay of main determinants of $k_{on}^{(mem)}/k_{on}^{(h)}$ as functions of $d/L$. (C) The log-log plot of collision probability as a function of binder 'concentration' in the shell, indicating that they are nearly reciprocal; this explains the low sensitivity of $k_{on}^{(mem)}/k_{on}^{(h)}$ to $L$.

The low sensitivity of the coefficient of proportionality between $k_{on}^{(mem)}$ and $k_{on}^{(h)}$ to $L$ in the limit $D_{mem} \ll D_{vol}$ is explained by the interplay of the collision probability and the normalized binder 'concentration' within the shell, another determinant of $\frac{k_{on}^{(mem)}}{k_{on}^{(h)}}$ (Figure S4B). Indeed, these factors vary with $\frac{d}{L}$ in a nearly reciprocal manner (Figure S4C).

That the value of $\frac{k_{on}^{(mem)}}{k_{on}^{(h)}}$ in the limit $D_{mem} \ll D_{vol}$ is close to that in the case of equal diffusivities is largely due to the assumption of well-mixed anchors: the averaging of $p_{coll}(l) = \frac{v_{shared}(l)}{v_{shell}}$ is done over the uniform anchor distribution (Eq (S3)), and the number of binding partners (Eq (S1)) assumes that the anchors are well-mixed.

We now turn to cases of flexible tethers, again assuming the diameter of a binder $d$ to be significantly less than the tether length $L$. If a tether is totally flexible, then the entire hemisphere, centered at the anchor, is accessible to the binder, so we have a case of $r = 0$ and, consequently, $a = 0$. For completeness, we will consider a 'semiflexible' tether model, where a portion of the tether adjacent to the anchor is stiff, whereas the remaining segment is flexible, so that the binder again is found with equal probability in a hemispherical shell with the inner radius $r$, though in this case $(R - r) \neq d$ .

We recall that Eq (S5) holds only for $a \geq \frac{1}{3}$. For $a < \frac{1}{3}$, the stereometry yields the following equation for $v_{shared}/(\frac{2\pi R^3}{3})$,

$$\frac{v_{\ shared}(\rho)}{(2\pi R^3/3)} = \begin{cases} f(\rho) - a^3 f\left(-\frac{\rho}{a}\right), & \text{for } \rho \in [0,\ a] \\ f(\rho) - 2a^3, & \text{for } \rho \in \left[a,\ \frac{1}{2}(1-a)\right] \\ f(\rho) - f\left(\frac{1-a^2}{4\rho} + \rho\right) - a^3 f\left(\frac{a^2-1}{4\rho a} + \frac{\rho}{a}\right), & \text{for } \rho \in \left[\frac{1}{2}(1-a),\ \frac{1}{2}(1+a)\right] \\ f(\rho), & \text{for } \rho \in \left[\frac{1}{2}(1+a), 1\right] \end{cases} \quad \text{. Eq (S8)}$$

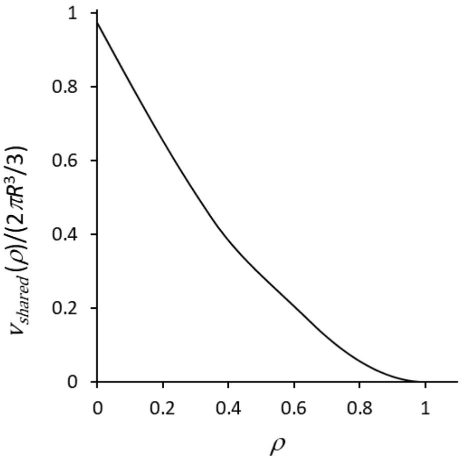


**Figure S5. Volume shared by two identical shells as a function of distance between their centers.**
The volume of the shared space, normalized by volume of the hemisphere, is shown as a function of $\rho = \frac{l}{2R}$; $\frac{r}{R} =$

Figure S5 illustrates $v_{shared}/(\frac{2\pi R^3}{3})$ as a function of $\rho$ for $a = 0.3$.

Interestingly, while Eq (S8) differs from Eq (S5), its integral is the same as in Eq (S6), so Eqs (S7) hold for all $a \in [0, 1]$. Thus, for the case of 'semiflexible' tethers, the ratio $k_{on}^{(mem)}/(\frac{k_{on}^{(vol)}}{R})$ as a function of $a = \frac{r}{R}$ is described by Eq (S7b); the graph of the ratio as a function of a flexible fraction of the tether is shown in Figure S6.

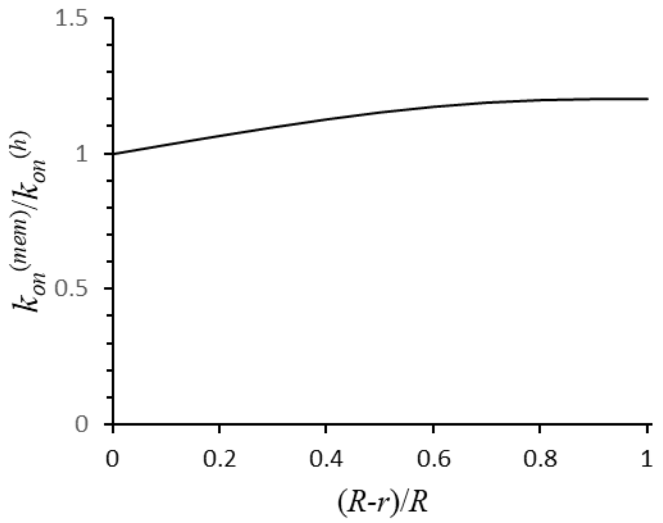


**Figure S6. Ratio $k_{on}^{(mem)}/k_{on}^{(h)}$ for the case of monomers with (semi)flexible tethers.** The ratio is shown as a function of a flexible fraction of the tether.

For the fully flexible tether, it follows from Eq (S7b) that $\phi(0) = 1.2$, therefore $k_{on}^{(mem)} = \phi(0)\frac{k_{on}^{(vol)}}{R} = 1.2k_{on}^{(h)}$.

## II. Applicability of mass-action kinetics to dimerization of molecules tethered to the membrane.

Determining the equivalent two-dimensional (2D) rate constant $k_{on}^{(mem)}$ for dimerization of monomers tethered to the membrane is based on the assumption that its kinetics can be accurately approximated as $k\sigma_A\sigma_B$, where $\sigma_A(t)$ and $\sigma_B(t)$ are the 2D densities of the binding partners $A$ and $B$, and $k$ is the rate constant, i.e. it is independent of time. In the case of homodimerization, the corresponding rate equation, $\partial_t\sigma = -2k\sigma^2$, has the exact solution,

$$\sigma(t) = \frac{\sigma_0}{1+2k\sigma_0 t}, \qquad \text{Eq (S9)}$$

where $\sigma_0$ is the initial monomer concentration, $\sigma_0 = \sigma(0)$. In part I of this text, we showed that if the binding partners remain distributed uniformly during the entire process, the dimerization is indeed described by mass-action kinetics and used this assumption to derive $k_{on}^{(mem)}$. If the mechanism of monomer mixing is the diffusion of their anchors, it is qualitatively clear that the

mass-action rate law would apply assuming high anchor diffusivity and slow association of the binding sites upon collision. Under these conditions, the mass-action rate constant essentially coincides with the rate constant of the intrinsic binding. However, this assumption is not always true, as some examples of the main text indicate. In this section, we use the idealized cases presented in Table 1 and the first row of Table 2 of the main text to provide insight into factors affecting applicability of mass-action kinetics and further validate our simulation results.

*Binding of molecules embedded in the membrane* ($L \to 0$).
It is instructive to first analyze the system in the limit of $L \to 0$ and then generalize the results to nonzero $L$. The criteria of applicability of mass-action kinetics in this limit can be studied by applying the Smoluchowski theory, which was extensively reviewed in the past, see, e.g., (Keizer, 1987); for more recent overview, see (Yogurtcu and Johnson, 2015) and references therein. The theory assumes that the association, or intrinsic binding, of the molecules occurs on collision with some rate constant $k_0$, whereas the observed rate coefficient, which is also influenced by molecular diffusion, could be time-dependent and therefore may not be accurately described by mass-action kinetics. However, for the reaction-limited binding characterized by high diffusivity of binding partners and slow initrinsic binding, the effects of diffusion are negligible, so that the observable rate coefficient coincides with $k_0$ and the mass-action approximation applies both in 3D and 2D.

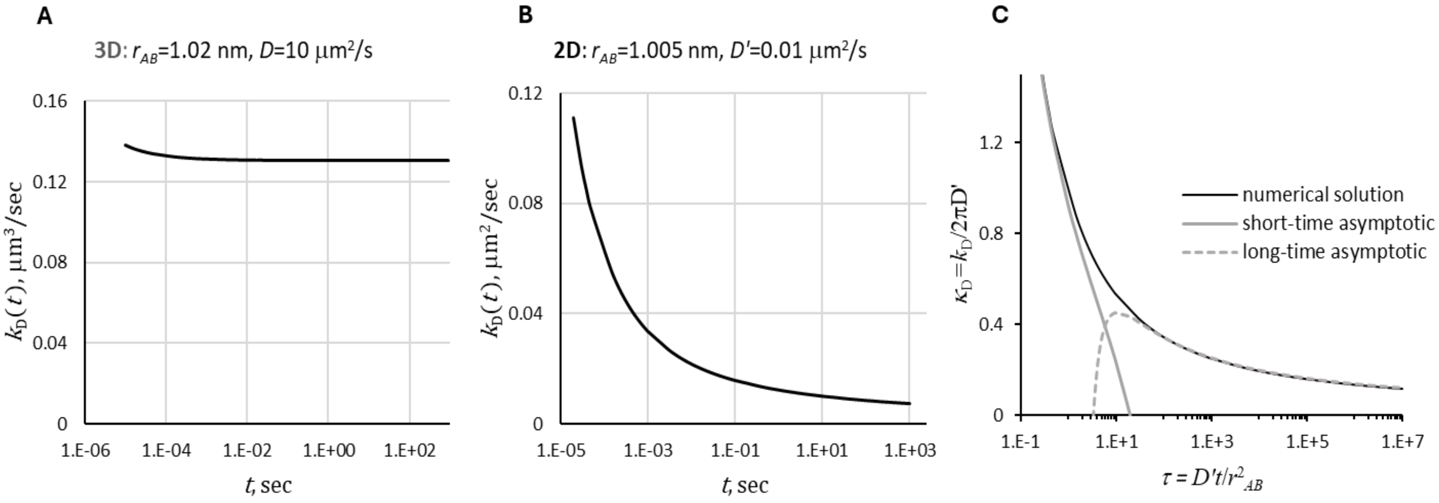


**Figure S7. Time dependence of $k_D$ for diffusion-limited binding in 3D and 2D.**
Time dependencies $k_D(t)$ in 3D (A) and 2D (B) shown for typical values of $r_{AB}$ and $D'$.
(C) Numerically obtained time dependence of the 2D binding rate coefficient, shown in dimensionless coordinates $\kappa_D = k_D/(2\pi)$ and $\tau = D't/r_{AB}^2$ (black solid curve), is compared with analytical short-time and long-time asymptotic solutions (Torney, McConnell, 1983; Barzykin and Tachiya, 1993): $\kappa_D = \frac{1}{\sqrt{\pi\tau}} + \frac{1}{2} - \frac{1}{4}\sqrt{\frac{\tau}{\pi}} + \ldots$ (grey solid curve) and $\kappa_D = 2\left(\xi - \gamma\xi^2 - \left(\frac{\pi^2}{6} - \gamma^2\right)\xi^3 + \cdots\right)$, where $\xi = \frac{1}{\ln(4\tau) - 2\gamma}$ and Euler's constant $\gamma = 0.5772157\ldots$ . (grey dashed curve).

In contrast, for diffusion-limited reactions characterized by fast initrinsic binding ($k_0 \to \infty$), the 3D kinetics differs significantly from that in 2D. While the theory yields time-dependent rate coefficients for both 2D and 3D, the rate coefficient of diffusion-limited binding in 3D relatively quickly approaches its steady value $k_D = 4\pi r_{AB} D'$, where $r_{AB}$ is the sum of effective radii of the

binding partners, $r_{AB} = r_A + r_B$ , and $D'$ is the sum of their diffusion coefficients, $D' = D_A + D_B$ (Figure S7A). Thus, bimolecular binding in 3D is in effect well described by mass-action kinetics both in the association- and diffusion-dominated limits.

In the intermediate regime with finite $k_0$ and $k_D$, sometimes termed “diffusion-influenced”, the 3D binding is also well approximated by mass-action kinetics (Collins and Kimball, 1949) with the rate constant

$$k = \frac{k_0 k_D}{k_0 + k_D} . \qquad \text{Eq (S10)}$$

The situation in 2D is significantly different (Figure S7B), as the rate coefficient $k_D(t)$ in this case does not stabilize after the intial steep decrease, but rather continues descending to zero (Torney and NcConnel, 1983). This is a consequence of the loss of dimensionality, which reducies to zero the probability for a molecular pair to escape an encounter, resulting in depletion zones around the binders that have not yet reacted. In Figure S7C, the black solid curve represents the “universal” time dependence of the rate coefficient for the diffusion-limited binding in 2D, with $k_D$ scaled by $2\pi D'$ and $t$ scaled by $r_{AB}^2/D'$, see *Computational details* below.

**Table S1. Normalized rate coefficient for diffusion-limited binding in 2D as a function of dimensionless time**

| $D't/r_{AA}^2$ | $k_D/2\pi D'$ | $D't/r_{AA}^2$ | $k_D/2\pi D'$ | $D't/r_{AA}^2$ | $k_D/2\pi D'$ | $D't/r_{AA}^2$ | $k_D/2\pi D'$ | $D't/r_{AA}^2$ | $k_D/2\pi D'$ | $D't/r_{AA}^2$ | $k_D/2\pi D'$ | $D't/r_{AA}^2$ | $k_D/2\pi D'$ | $D't/r_{AA}^2$ | $k_D/2\pi D'$ |
|---|---|---|---|---|---|---|---|---|---|---|---|---|---|---|---|
| 0.198015 | 1.766791 | 188.1142 | 0.313627 | 584.144 | 0.268346 | 980.1738 | 0.25157 | 39602.98 | 0.173029 | 79205.96 | 0.163375 | 2079156 | 0.129257 | 6039454 | 0.120976 |
| 0.297022 | 1.49689 | 198.0149 | 0.311271 | 594.0447 | 0.267768 | 990.0745 | 0.251264 | 40593.05 | 0.172666 | 80196.03 | 0.163212 | 2178164 | 0.128867 | 6138462 | 0.120858 |
| 0.39603 | 1.344426 | 207.9156 | 0.309059 | 603.9454 | 0.267202 | 1980.149 | 0.233219 | 41583.13 | 0.172312 | 81186.11 | 0.163051 | 2277171 | 0.128501 | 6237469 | 0.120742 |
| 0.495037 | 1.241926 | 217.8164 | 0.306978 | 613.8462 | 0.266647 | 2970.224 | 0.222209 | 42573.2 | 0.171969 | 82176.18 | 0.162892 | 2376179 | 0.128154 | 6336477 | 0.120628 |
| 0.990075 | 1.007724 | 227.7171 | 0.305014 | 623.7469 | 0.266103 | 3960.298 | 0.21527 | 43563.28 | 0.171636 | 83166.26 | 0.162734 | 2475186 | 0.127822 | 6435484 | 0.120517 |
| 1.485112 | 0.879671 | 237.6179 | 0.303155 | 633.6477 | 0.26557 | 4950.373 | 0.210245 | 44553.35 | 0.171311 | 84156.33 | 0.162579 | 2574194 | 0.127505 | 6534492 | 0.120407 |
| 1.980149 | 0.806328 | 247.5186 | 0.301392 | 643.5484 | 0.265047 | 5940.447 | 0.206333 | 45543.43 | 0.170994 | 85146.41 | 0.162427 | 2673201 | 0.127201 | 6633499 | 0.120298 |
| 2.475186 | 0.75647 | 257.4194 | 0.299724 | 653.4492 | 0.264534 | 6930.522 | 0.203148 | 46533.5 | 0.170685 | 86136.48 | 0.162278 | 2772209 | 0.126909 | 6732507 | 0.120192 |
| 2.970224 | 0.719406 | 267.3201 | 0.298137 | 663.3499 | 0.264031 | 7920.596 | 0.20047 | 47523.58 | 0.170384 | 87126.56 | 0.16213 | 2871216 | 0.12663 | 6831514 | 0.120087 |
| 3.465261 | 0.690319 | 277.2209 | 0.296615 | 673.2507 | 0.263537 | 8910.671 | 0.198167 | 48513.65 | 0.170089 | 88116.63 | 0.161984 | 2970224 | 0.126361 | 6930522 | 0.119985 |
| 3.960298 | 0.666675 | 287.1216 | 0.295158 | 683.1514 | 0.263052 | 9900.745 | 0.196154 | 49503.73 | 0.169801 | 89106.71 | 0.16184 | 3069231 | 0.126101 | 7029529 | 0.119883 |
| 4.455335 | 0.646923 | 297.0224 | 0.293763 | 693.0522 | 0.262575 | 10890.82 | 0.194369 | 50493.8 | 0.169521 | 90096.78 | 0.161696 | 3168238 | 0.125851 | 7128536 | 0.119784 |
| 4.950373 | 0.630062 | 306.9231 | 0.292425 | 702.9529 | 0.262107 | 11880.89 | 0.192767 | 51483.87 | 0.169246 | 91086.85 | 0.161555 | 3267246 | 0.12561 | 7227544 | 0.119685 |
| 5.44541 | 0.615425 | 316.8238 | 0.291141 | 712.8536 | 0.261647 | 12870.97 | 0.191317 | 52473.95 | 0.168977 | 92076.93 | 0.161417 | 3366253 | 0.125377 | 7326551 | 0.119589 |
| 5.940447 | 0.602536 | 326.7246 | 0.289907 | 722.7544 | 0.261194 | 13861.04 | 0.189994 | 53464.02 | 0.168715 | 93067 | 0.16128 | 3465261 | 0.125151 | 7425559 | 0.119493 |
| 6.435484 | 0.591071 | 336.6253 | 0.288719 | 732.6551 | 0.26075 | 14851.12 | 0.188779 | 54454.1 | 0.168458 | 94057.08 | 0.161144 | 3564268 | 0.124932 | 7524566 | 0.1194 |
| 6.930522 | 0.580771 | 346.5261 | 0.287574 | 742.5559 | 0.260312 | 15841.19 | 0.187655 | 55444.17 | 0.168206 | 95047.15 | 0.16101 | 3663276 | 0.12472 | 7623574 | 0.119307 |
| 7.425559 | 0.571452 | 356.4268 | 0.28647 | 752.4566 | 0.259882 | 16831.27 | 0.186613 | 56434.25 | 0.16796 | 96037.23 | 0.160878 | 3762283 | 0.124515 | 7722581 | 0.119216 |
| 7.920596 | 0.562964 | 366.3276 | 0.285405 | 762.3574 | 0.259459 | 17821.34 | 0.185639 | 57424.32 | 0.167718 | 97027.3 | 0.160746 | 3861291 | 0.124315 | 7821589 | 0.119126 |
| 8.415633 | 0.555171 | 376.2283 | 0.284374 | 772.2581 | 0.259042 | 18811.42 | 0.184728 | 58414.4 | 0.167481 | 98017.38 | 0.160617 | 3960298 | 0.124122 | 7920596 | 0.119038 |
| 8.910671 | 0.548035 | 386.1291 | 0.283378 | 782.1589 | 0.258632 | 19801.49 | 0.183873 | 59404.47 | 0.167249 | 99007.45 | 0.160489 | 4059305 | 0.123933 | 8019603 | 0.11895 |
| 9.405708 | 0.541435 | 396.0298 | 0.282412 | 792.0596 | 0.258229 | 20791.56 | 0.183066 | 60394.54 | 0.167021 | 198014.9 | 0.152737 | 4158313 | 0.12375 | 8118611 | 0.118864 |
| 9.900745 | 0.535248 | 405.9305 | 0.281477 | 801.9603 | 0.257831 | 21781.64 | 0.182303 | 61384.62 | 0.166798 | 297022.4 | 0.147872 | 4257320 | 0.123571 | 8217618 | 0.118779 |
| 19.80149 | 0.467884 | 415.8313 | 0.28057 | 811.8611 | 0.25744 | 22771.71 | 0.18158 | 62374.69 | 0.166579 | 396029.8 | 0.144723 | 4356328 | 0.123397 | 8316626 | 0.118695 |
| 29.70224 | 0.428993 | 425.732 | 0.27969 | 821.7618 | 0.257054 | 23761.79 | 0.180893 | 63364.77 | 0.166364 | 495037.3 | 0.142402 | 4455335 | 0.123227 | 8415633 | 0.118613 |
| 39.60298 | 0.405744 | 435.6328 | 0.278835 | 831.6626 | 0.256674 | 24751.86 | 0.180238 | 64354.84 | 0.166153 | 594044.7 | 0.140573 | 4554343 | 0.123062 | 8514641 | 0.118531 |
| 49.50373 | 0.38948 | 445.5335 | 0.278004 | 841.5633 | 0.2563 | 25741.94 | 0.179614 | 65344.92 | 0.165946 | 693052.2 | 0.139067 | 4653350 | 0.122901 | 8613648 | 0.11845 |
| 59.40447 | 0.377121 | 455.4343 | 0.277195 | 851.4641 | 0.255931 | 26732.01 | 0.179018 | 66334.99 | 0.165742 | 792059.6 | 0.137791 | 4752358 | 0.122743 | 8712656 | 0.118371 |
| 69.30522 | 0.367247 | 465.335 | 0.276409 | 861.3648 | 0.255568 | 27722.09 | 0.178447 | 67325.07 | 0.165541 | 891067.1 | 0.136687 | 4851365 | 0.122589 | 8811663 | 0.118292 |
| 79.20596 | 0.359101 | 475.2358 | 0.275643 | 871.2656 | 0.255209 | 28712.16 | 0.177899 | 68315.14 | 0.165344 | 990074.5 | 0.135714 | 4950373 | 0.122438 | 8910671 | 0.118215 |
| 89.10671 | 0.352197 | 485.1365 | 0.274897 | 881.1663 | 0.254856 | 29702.24 | 0.177373 | 69305.22 | 0.165151 | 1089082 | 0.134848 | 5049380 | 0.122291 | 9009678 | 0.118138 |
| 99.00745 | 0.346229 | 495.0373 | 0.27417 | 891.0671 | 0.254507 | 30692.31 | 0.176866 | 70295.29 | 0.16496 | 1188089 | 0.134066 | 5148387 | 0.122147 | 9108685 | 0.118062 |
| 108.9082 | 0.340991 | 504.938 | 0.27346 | 900.9678 | 0.254163 | 31682.38 | 0.176379 | 71285.36 | 0.164773 | 1287097 | 0.133355 | 5247395 | 0.122006 | 9207693 | 0.117988 |
| 118.8089 | 0.336338 | 514.8387 | 0.272768 | 910.8685 | 0.253824 | 32672.46 | 0.175909 | 72275.44 | 0.164589 | 1386104 | 0.132704 | 5346402 | 0.121868 | 9306700 | 0.117914 |
| 128.7097 | 0.332161 | 524.7395 | 0.272092 | 920.7693 | 0.253489 | 33662.53 | 0.175456 | 73265.51 | 0.164407 | 1485112 | 0.132103 | 5445410 | 0.121733 | 9405708 | 0.117841 |
| 138.6104 | 0.328384 | 534.6402 | 0.271432 | 930.67 | 0.253159 | 34652.61 | 0.175018 | 74255.59 | 0.164228 | 1584119 | 0.131546 | 5544417 | 0.121601 | 9504715 | 0.117769 |
| 148.5112 | 0.324943 | 544.541 | 0.270787 | 940.5708 | 0.252833 | 35642.68 | 0.174595 | 75245.66 | 0.164052 | 1683127 | 0.131028 | 5643425 | 0.121471 | 9603723 | 0.117697 |
| 158.4119 | 0.32178 | 554.4417 | 0.270157 | 950.4715 | 0.252511 | 36632.76 | 0.174185 | 76235.74 | 0.163879 | 1782134 | 0.130542 | 5742432 | 0.121344 | 9702730 | 0.117627 |
| 168.3127 | 0.318856 | 564.3425 | 0.26954 | 960.3723 | 0.252194 | 37622.83 | 0.173787 | 77225.81 | 0.163708 | 1881142 | 0.130087 | 5841440 | 0.121219 | 9801738 | 0.117557 |
| 178.2134 | 0.316148 | 574.2432 | 0.268937 | 970.273 | 0.25188 | 38612.91 | 0.173402 | 78215.89 | 0.163541 | 1980149 | 0.129663 | 5940447 | 0.121096 | 9900745 | 0.117488 |

It was obtained by solving the Smoluchowski model numerically with *Virtual Cell* (VCell), a suite of numerical tools for solving models arising in cell biology (Slepchenko and Loew, 2010; Resasco et al., 2012). The values of $k_D/2\pi D'$ as a function of $D't/r_{AB}^2$ are shown in Table S1.

---

*Computation details*

The Smoluchowski model defines the binding rate coefficient $k(t)$ in terms of pair survival probability $u(r,t)$, the probability that an isolated pair of binding partners, initially separated by distance $r$, does not react by time $t$. Here and below, we assume uniformity of space, system axial symmetry, and that irreversible binding occurs on encounter. Without loss of generality, one of the binding partners (molecule A) can be fixed at the origin and assigned the effective reaction radius, $r_{AB} = r_A + r_B$, whereas the center of the other binding partner (molecule B) diffuses with the effective diffusion coefficient $D' = D_A + D_B$ in the domain $r \in [r_{AB}, \infty)$. The survival probability $u$ is governed by the equation, $u_t = D'\Delta u$, where $\Delta$ is the 2D diffusion operator, $\Delta = \frac{1}{r}\partial_r(r\partial_r\ )$, see, e.g., (Barzykin and Tachiya, 1993).The governing equaton is solved with the flux boundary condition, $2\pi r_{AB}\partial_r u(r_{AB},t) = k_0 u(r_{AB},t)$, the initial condition $u(r,0) = 1$, and the boundary condition $u(\infty,t) = 1$ at $r_{AB} \to \infty$. The 'observed' reacton rate coefficient $k(t)$ is then defined as $k(t) = 2\pi r_{AB}\partial_r u(r_{AB},t)$. For diffusion-limited reactions, $k_0 \to \infty$, so the boundary condition at $r = r_{AB}$ becomes $u(r_{AB},t) = 0$. Thus, $k_D(t) = 2\pi r_{AB}\partial_r u(r_{AB},t)$ for the reactive boundary condition $u(r_{AB},t) = 0$.

To solve this model with VCell, the governing equation must be rewritten in a 1D 'Cartesian' diffusion-advection form: using the change of variables, $u = U/r$, the equation becomes $U_t = D'\partial_{rr}^2 U - \partial_r(vU)$, with $v = D'/r$. To avoid numerical spatial differentiation, which is lower order of accuracy than numerical integration, we solved the model both outside and inside the reaction boundary. Outside the reactive boundary, i.e., for $r \in [r_{AB}, r_{max}]$ , the equation for $U$ was solved using the flux density $f_U(r_{AB},t) = -k_0 U(r_{AB},t)$ with sufficiently large $k_0$, the initial condition $U(r,0) = r$, and the boundary condition $f_U(r_{max},t) = 0$ at $r = r_{max}$. Inside the reactive boundary, i.e., for $r \in [0, r_{AB})$, we integrate the flux across the reactive boundary over time, $I(t) = \int_0^t k_D(t)dt$ . For this, we solve there the 1D diffusion equation, $u_t = D\partial_{rr}^2 u,$ with sufficiently high diffusivity $D$, so that $u(r,t)$ is equal to its average $\bar{u}(t)$ with high precision. The equation was solved with the flux boundary condition, $f_u(r_{AB},t) = f_U(r_{AB},t)/r$ and $f_u(0,t) = 0$, and the intial condition $u(r,0) = 0$. Thus, $\bar{u}(t)r_{AB}$ yields the integrated flux density and the integrated flux $I(t)$ is given by $2\pi r_{AB}^2\bar{u}(t)$. Finally we determine $k_D(t)$ by numerical time differentiation of $\bar{u}(t)$, $k_D(t) = \frac{dI}{dt} = 2\pi r_{AB}^2\bar{u}_t$. The computations were performed using VCell fully-implicit finite-volume solver (Slepchenko et al, 2018) for $r_{AB} = 0.001005$ μm, $r_{max} = 10$ μm, $D' = 0.01$ μm²/s. Results obtained with the increasing $k_0$ and decreasing space discretization parameter $\Delta r$, showed that the relative numerical errors of the solution with $k_0 = 10^6$ μm²/s and $\Delta r = 10^{-5}$ μm were within few percent. Comparison against the short-time and long-time analytical asymptotic solutions (Figure S7C) indicated relative errors in the 2-4% range. We nondimensionalized the solution obtained with $k_0 = 10^6$ μm²/s and $\Delta r = 10^{-5}$ μm to obtained the universal dependence of $k_D/2\pi D'$ on normalized time $\tau = D't/r_{AB}^2$ (Table S1).VCell implementation of the model can be found in the public VCell MathModel database under 'boris'. Model name: Diffusion_limited_binding_2D'; simulations: Simulation3 and its copies.

---

Since the function $\kappa_D(\tau)$ in Figure S7C approaches zero as $\tau \to \infty$ and diverges to infinity when $\tau \to 0$, the 2D bimolecular binding in the intermediate regime with finite intrinsic rate constants $k_0$ always begins as reaction-limited and thus initially is well approximated by mass-action kinetics, but eventually crosses over to the diffusion-limited mode with a time-dependent rate coefficient. It is therefore clear that in 2D, the mass-action kinetics given by Eq (S9) with a single-valued $k$ cannot adequately describe the kinetics of diffusion-influenced binding over arbitrary time periods. However, as we explain later, under certain conditions mass-action kinetics can be a reasonably accurate approximation even for diffusion-limited reactions.

The time dependence of $k_D$ in 2D renders the corresponding observed rate coefficient time-dependent as well, due to the general relation between the pair survival probabilities in the diffusion-limited and intermediate regimes (Pedersen, 1980), which holds for the systems of all dimensions (see Appendix in (Tachiya, 1983)). This property, originating from linearity of the Smoluchowski model and the fact that the two regimes differ only by conditions at the reactive boundary and not by the equation itself, allows one to connect the kinetic coefficients of the two regimes (Szabo, 1989),

$$\hat{k}(s) = k_0\hat{k}_D(s)/(k_0 + s\hat{k}_D(s)), \qquad \text{Eq (S11)}$$

where $\hat{k}_D(s) = \int_0^\infty k_D\,(t)e^{-st}dt$ is the Laplace transform of $k_D(t)$, and $\hat{k}(s) = \int_0^\infty k(t)e^{-st}dt$ is the Laplace transform of the observed rate coefficient $k(t)$ in the intermediate regime with a finite $k_0$; the Laplace variable $s$ has units of the inverse time. Note that Eq (S11) reduces to Eq (S10), in 3D, where both $k$ and $k_D$ are effectively constant on the time scale of interest.

In lower dimensions, the meaning of $s\hat{k}_D(s) = \frac{1}{s^{-1}}\int_0^\infty k_D(t)\exp\left(-\frac{t}{s^{-1}}\right)dt$ is that of a weighted average of $k_D(t)$ over time $t\sim s^{-1}$, given that the main contributions to the integral come from the times $t$ that do not significantly exceed $s^{-1}$; we therefore denote $s\hat{k}_D(s)$ as $\bar{k}_D(s^{-1})$,

$$\bar{k}_D(s^{-1}) = \frac{1}{s^{-1}}\int_0^\infty k_D(t)\exp\left(-\frac{t}{s^{-1}}\right)dt. \qquad \text{Eq (S12)}$$

We define time to completion $t_c$ as the time required for a reaction to reach a desired level of completion $p$, $p \in (0,1)$; so for irreversible reactions, $\sigma(t_c)/\sigma_0 = 1 - p$ (for reversible reactions, see subsection *Reversible binding of tethered monomers*). While Eq (S11) holds for arbitrary $s$, we are interested in $s^{-1} = t_c$, so from Eqs (S12),

$$\bar{k}_D(t_c) = \frac{1}{t_c}\int_0^\infty k_D\,(t)\exp\left(-\frac{t}{t_c}\right)dt\ , \qquad \text{Eq (S13)}$$

and from Eq (S11),

$$\bar{k}(t_c) = \frac{1}{t_c}\int_0^\infty k\,(t)\exp\left(-\frac{t}{t_c}\right)dt\ = k_0\bar{k}_D(t_c)/(k_0 + \bar{k}_D(t_c)); \qquad \text{Eq (S14)}$$

(note the structural similarity between Eq (S14) and Eq (S10)).

Yogurtcu and Johnson introduced a time-dependent parameter $\delta(t) = 1 - k(t)/k_0$ as a measure of deviation from reaction-limited kinetics (Yogurtcu and Johnson, 2015). We define a somewhat different measure, $\delta = \bar{k}(t_c)/\bar{k}_D(t_c)$, which for given $t_c$ quantitatively identifies reaction kinetics in the spectrum between the reaction-contolled ($\delta = 0$) and diffusion-limited ($\delta = 1$) regimes. Our analysis yields an exact explicit formula for $\delta$ in terms of $k_0$ and $k_D(t)$,

$$\delta = \frac{\bar{k}(t_c)}{\bar{k}_D(t_c)} = \left(1 + \frac{\bar{k}_D(t_c)}{k_0}\right)^{-1}, \qquad \text{Eq (S15)}$$

which follows from Eqs (S13, S12).

Note that while the condition $\delta \ll 1$, which indicates the association-influnced binding, is sufficient for the mass-action approximation, it is not necessary. As we demonstrate later, the mass-action approximation can be reasonably accurate under certain conditions for $\delta \approx 1$ as well.

*Irreversible binding of tethered monomers.*
Generalizing the theory to dimerization of tethered molecules depends on dimensionless parameter $\sigma_0\pi h^2$ determining how many neighboring binders are initially within the reach of a given monomer.

We first consider cases with $\sigma_0\pi h^2 < 1$ (exemplified by first eight rows in Table 1 of the main text). For these cases, the reactive boundary is a hemisphere with radius $h$ centered at a monomer's anchor.Then the corresponding effective 2D radius, which is the average of tether projections on the membrane, is $r_A^{(2D)} = h/2$; therefore $r_{AA}^{(2D)} = 2r_A^{(2D)} = h$. One can also show that upon projection onto the membrane, the effective membrane diffusivity of the binder is $D_A^{\text{eff}} = D_{mem}/2$, and therefore $D' = 2D_A^{\text{eff}} = D_{mem}$ .Given these parameters, we can determine $k_D(t)$ by utilizing $\kappa_D(\tau)$ from Table S1 (see also the corresponding Excell worksheet in the supplimentary files): $k_D(t) = 2\pi D_{mem} \cdot \kappa_D(D_{mem}t/h^2)$.

Noting that the association rate constant is now defined as $k_{on}^{(h)} = k_{on}^{(vol)}/h$, we can evaluate the integral in Eq (S13) and then use Eqs (S14) and (S15) to compute weighted averages of the irreversible-binding rate coefficient $\bar{k}_{irr}(t_c)$ and the corresponding $\delta$, for any combination of $k_{on}^{(h)}$, $h$, $D_{mem}$, and $t_c$ (note that, as we discuss later, $t_c$ is affected by an initial monomer density $\sigma_0$, level of reaction completion $p$, as well as by either $k_{on}^{(h)}$ or $D_{mem}$, depending on whether the kinetics is association- or diffusion-influenced).

Table S2 below presents values of $\bar{k}_{irr}(t_c)$ obtained with $h$ = 5.5 nm for varying parameter sets, in comparison with the corresponding values of $k_{on}^{(mem)}$, as the latter are also averages of $k_{irr}(t)$ yielded by fitting Eq (S9) to results of SpringSaLaD simulations with the same parameter sets, see also Figure 3 in the main text. The times to completion $t_c$ (column 7), corresponding to $p =$

**Table S2. $k_{on}^{(mem)}$ from SpringSaLaD simulations vs. $\bar{k}_{irr}(t_c)$ based on Smoluchowski theory for cases with $\sigma_0\pi h^2 < 1$**

| row ## | $\sigma_0$, µm$^{-2}$ | $D_{mem}$, µm$^2$/s | $k_{on}^{(h)}$, µm$^2$/s | $k_{on}^{(mem)}$, µm$^2$/s | Relative RMSD | $t_c$, s | $\bar{k}_{irr}(t_c)$, µm$^2$/s | δ |
|---|---|---|---|---|---|---|---|---|
| 1 | 2500 | 0.01 | 0.003 | 0.0029 | 0.0055 | 0.2695 | 0.0025 | 0.16 |
| 2 | 2500 | 1 | 0.003 | 0.0031 | 0.0055 | 0.2645 | 0.0030 | 0.0019 |
| 3 | 25 | 0.01 | 0.003 | 0.0024 | 0.0053 | 33.78 | 0.0023 | 0.23 |
| 4 | 25 | 1 | 0.003 | 0.0030 | 0.0048 | 26.62 | 0.0030 | 0.0030 |
| 5 | 2500 | 0.01 | 0.3 | 0.084 | 0.086 | 0.0138 | 0.023 | 0.97 |
| 6 | 2500 | 1 | 0.3 | 0.26 | 0.016 | 0.0033 | 0.27 | 0.085 |
| 7 | 25 | 0.01 | 0.3 | 0.018 | 0.039 | 5.372 | 0.011 | 0.96 |
| 8 | 25 | 1 | 0.3 | 0.20 | 0.022 | 0.425 | 0.25 | 0.092 |
| 9 | 25 | 0.01 | 0.03 | 0.012 | 0.02 | 4.0000 | 0.0086 | 0.71 |
| 10 | 25 | 1 | 0.03 | 0.029 | 0.009 | 2.8300 | 0.029 | 0.024 |
| 11 | 2500 | 1 | 0.03 | 0.029 | 0.01 | 0.0264 | 0.029 | 0.030 |
| 12 | 2500 | 0.01 | 0.03 | 0.020 | 0.03 | 0.0436 | 0.012 | 0.59 |
| 13 | 250 | 0.01 | 0.003 | 0.0025 | 0.008 | 2.0000 | 0.0024 | 0.19 |
| 14 | 250 | 0.01 | 0.3 | 0.031 | 0.05 | 0.3130 | 0.015 | 0.95 |
| 15 | 250 | 1 | 0.003 | 0.003 | 0.009 | 2.5000 | 0.0030 | 0.0024 |
| 16 | 250 | 1 | 0.3 | 0.23 | 0.01 | 0.0374 | 0.26 | 0.13 |

0.8 in all rows, except for rows 10 ($p = 0.69$), 13 ($p = 0.72$), and 15 ($p = 0.795$), were retrieved from the simulated dimer densities $[dimer]_t$.The accuracy of fitting was characterized by computing the relative root-mean-square deviation (relative RMSD) defined as $\left(\sum_i([dimer]_{t_i}^{sim} - ([dimer]_{t_i}^{appr})\right)^2 / \sum_i([dimer]_{t_i}^{sim})^2)^{1/2}$, where the summation was perfomed over all time points $t_i \leq t_c$. The difference in the first sum is between the simulated and approximated by mass-action kinetics dimer densities corresponding to same $t_i$.

The comparison of the data in columns 5 and 8 (highlighted) indicates similar trends in responses to parameter changes. As expected, more pronounced differences between $k_{on}^{(mem)}$ and $\bar{k}_{irr}(t_c)$ correlate with higher relative RMSD (rows 5, 7, 9, 12, and 14), but even in these cases, the respective values are of same order of magnitude. Overall, the degree of correspondence gives credence to both the simulations and theory.

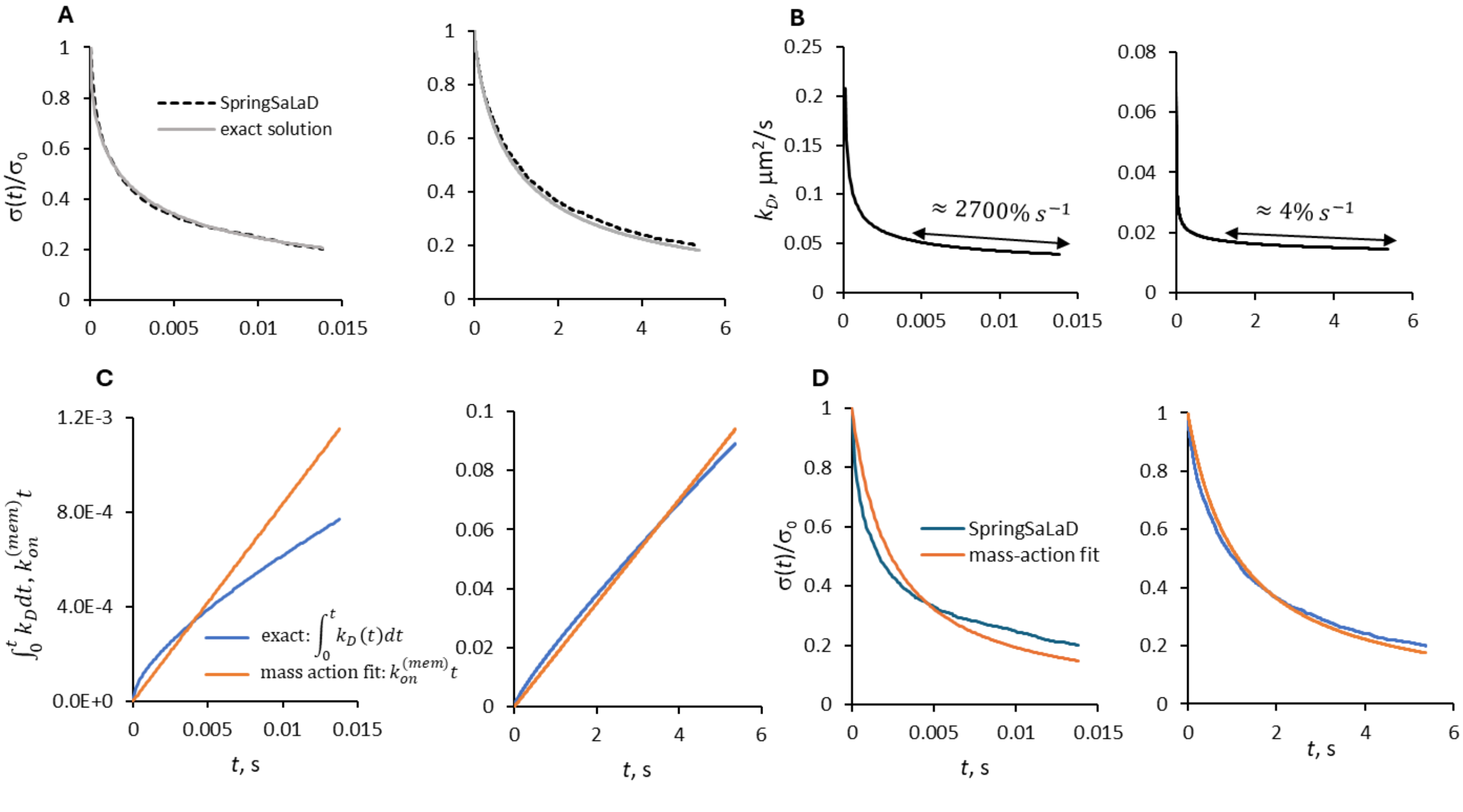


**Figure S8. Comparing conditions of rows 5 and 7 of Table 1: Slow diffusion-limited binding is accurately described by mass-action kinetics.**

(A) SpringSaLaD solutions (black dashed curves) are consistent with exact diffusion-limited kinetics, $\sigma(t)/\sigma_0 = \left(1 + 2\sigma_0 \int_0^t k_D(t)dt\right)^{-1}$ (grey solid curves), both for the fast reaction with $\sigma_0 = 2500$ μm$^{-2}$ (left panel) and the slow one with $\sigma_0 = 25$ μm$^{-2}$ (right panel). (B) Because of the same $r_{AA}^{(2D)} = h$ and $D' = D_{mem}$ in both rows, the two reactions have the same $k_D(t)$ which is shown for the times to 80% completion of the fast (left) and slow (right) reactions. Average grades of $k_D(t)$ decline during the logarithmic phase, shown for the time intervals [0.005 s, 0.0138 s] (left) and [1 s, 5.372 s] (right), differ by more than two orders of magnitude on the two timescales, with the slow reaction having nearly constant rate coefficient. (C) As the fitting to mass-action kinetics (Eq(S9)) replaces the exact function $\int_0^t k_D(t')dt'$ with the approximate linear function $k_{on}^{(mem)} t$ , the two functions are compared using the best-fit values of $k_{on}^{(mem)}$ , for the fast (left) and slow (right) reactions. (D) Best mass-action fits of $\sigma(t)/\sigma_0$ for the fast (left) and slow (right) reactions.

Remarkably, the conditions described in rows 7 and 14 are effectively diffusion-limited ($\delta = 0.96$ and $0.95$, respectively), yet they are approximated relatively well by mass-action kinetics. To explain this, it is instructive to compare the reaction of row 7 to the one described in row 5. The latter is also diffusion-limited ($\delta = 0.97$) but its mass-action fit is much less accurfate. For both reactions, SpringSaLaD results are indeed close to the diffusion-limited kinetics $\sigma(t)/\sigma_0 = (1 + 2\sigma_0 \int_0^t k_D(t)dt)^{-1}$ (Figure S8A), which in and of itself provides yet another validation of the simulaiton results. The function $k_D(t)$ used in both cases is the same, as it is determined by the same $r_{AA}^{(2D)} = h = 5.5$ nm and $D' = D_{mem} = 0.01$ μm²/s. The only parameter that is different in the conditons of rows 5 and 7 is $\sigma_0$, which affects the time to complition $t_c$. Indeed, it follows from Eq (S9) that $\sigma(t)/\sigma_0 = 1 - p = (1 + 2\sigma_0 k t_c)^{-1}$, yielding $t_c = \frac{1}{2k\sigma_0}\frac{p}{(1-p)}$ . Thus, for a given $p$, higher values of $\sigma_0$ result in shorter $t_c$, and this makes the difference.

The effect of $t_c$ on applicability of mass-action kinetics has to do with biphasic behavior of $k_D(t)$ illustrated by its plots in linear coordinates in Figure S8B: after the initial steep descent, the function nearly plateaus as its decline becomes logarithmically slow. Thus, the slow diffusion-limited reaction operating on a long-time scale (large $t_c$) is characterized for most of the time by a nearly constant rate coefficient order $D_{mem}$ and, therefore, lends itself to more accurate approximation by mass-action kinetics (Figure S8C-D).

In cases with $\sigma_0 \pi h^2 > 1$, exemplified by row 1 in Table 2 of the main text, geometric constraints imposed by the tethers and slow anchor diffusion are less limiting in two aspects. First, multiple binding sites may now interact under the hemisphere of radius $h$. As a result, the tether length no longer represents the effective reaction radius, which is now determined by the radii of the binding sites $d/2$. Second, the main mixing mechanism is now volumetric diffusion. Because the area of the hemisphere is twice the area of its projection on the membrane, and both are covered by diffusion within the same time, the corresponding effective 2D diffusivity is $D_{vol}/2$. Thus, to compute $\bar{k}_{irr}(t_c)$ and $\delta$ for these cases, one can again use Eqs (S14) and (S15) with $r_{AA} = d/2$ and $D' = D_{vol}$.

The value of $\bar{k}_{irr}(t_c)$ in Table S3 was obtained with the same parameters as in Table 2, row 1 of the main text. It is in good agreement with the $k_{on}^{(mem)}$, obtained by fitting Eq (S9) to the SpringSaLaD simulations results, and characterized by low $\delta$, i.e. the kinetics in this example is well approximated by the mass-action rate law.

**Table S3. $k_{on}^{(mem)}$ from SpringSaLaD simulations vs. $\bar{k}_{irr}(t_c)$ based on Smoluchowski theory for cases with $\sigma_0 \pi h^2 > 1$**

| $\sigma_0$, μm$^{-2}$ | $D_{mem}$, μm²/s | $h$, nm | $k_{on}^{(h)}$, μm²/s | $k_{on}^{(mem)}$, μm²/s | Relative RMSD | $r_{AA}$, μm | $D'$, μm²/s | $t_c$, s | $\bar{k}_{irr}(t_c)$, μm²/s | δ |
|---|---|---|---|---|---|---|---|---|---|---|
| 2500 | 0.01 | 20.5 | 0.081 | 0.078 | 0.03 | 0.0005 | 1.0 | 0.0113 | 0.078434 | 0.03 |

Note that the inequality $\sigma(t)\pi h^2 > 1$ may not hold for all levels of completion $p$. Indeed, the left-hand side of the inequality changes as $\sigma(t)$ drops and the effective reach of the binding side increases due to anchor diffusion ($h_{\text{eff}}(t) = h + \Delta r_{\text{diff}}$) . Therefore, the maximum $\bar{p}$ for which $\sigma(t)\pi h_{\text{eff}}^2(t) > 1$ holds true can be estimated by solving the equation, $h + 2(D_{mem} t(\bar{p}))^{1/2} = (\pi(1 - \bar{p})\sigma_0)^{-1/2}$. Function $t(p)$ is the inverse of the dimer density time dependence, which can be retrieved from the SpringSaLaD simulation results for the example in Table S3. The equation

can also be solved directly assuming applicability of Eq (S9), from which $t(p) = \frac{1}{2k_{on}^{(h)}\sigma_0}\frac{p}{(1-p)}$. The substitution yields the equation, $h(\pi(1-\bar{p})\sigma_0)^{1/2} + (2\pi D_{mem}\bar{p}/k_{on}^{(h)})^{1/2} = 1$, which can be solved exactly. For the parameters in Table S3, $\bar{p} = 0.995$. Thus, the completion level $p$ =0.8, used for this example in SpringSaLaD and in estimating the corresponding $\bar{k}(t_c)$, satisfies the inequality $\sigma(t)\pi h_{\text{eff}}^2(t) > 1$ for all $t < \frac{1}{2k_{on}^{(h)}\sigma_0}\frac{p}{(1-p)}$.

*Reversible binding of tethered monomers.*
The mass-action rate law for reversible homodimerization is $\partial_t\sigma = -2k\sigma^2 + kK(\sigma_0 - \sigma)$, where $K$ is the equilibrium dissociation constant. Its exact solution $x(t) \equiv \frac{\sigma(t)}{\sigma_0}$ satisfies the following relation,

$$\frac{x(t)-x_1}{x(t)-x_2} = \frac{1-x_1}{1-x_2}\exp(-2k\sigma_0(x_1 - x_2)t), \qquad \text{Eq (S16)}$$

where $x_1$ is the normalized steady state, $x_1 = \sigma_{stedy\ state}/\sigma_0 = \frac{1}{2}(\sqrt{\alpha^2 + 4\alpha} - \alpha)$ and $x_2 = -\frac{1}{2}(\sqrt{\alpha^2 + 4\alpha} + \alpha)$, where $\alpha = K/2\sigma_0$. The level of completion $p$ in the reversible case reflects how close the reaction is to its steady state, i.e. $1 - p = (x(t_c) - x_1)/(1 - x_1)$.

Based on the theory of diffusion-influenced reversible reactions (Agmon and Szabo, 1990), the weighted average of a reversible-binding reaction rate coefficient, $\bar{k}_{rev}(t_c)$, satisfies the following equation,

$$(\bar{k}_{rev}(t_c))^{-1} = (\bar{k}_{irr}(t_c))^{-1} + t_cK = (\bar{k}_D(t_c))^{-1} + \left(k_{on}^{(h)}\right)^{-1} + t_cK. \qquad \text{Eq (S17)}$$

In the second equality of Eq (S17), we used Eq (S14). It follows from the first equality of Eq (S17) that $\bar{k}_{rev}(t_c) = \bar{k}_{irr}(t_c)(1 + t_cK\cdot\bar{k}_{irr}(t_c))^{-1}$; this formula was implemented in the corresponding supplementary Excell worksheet for computing $\bar{k}_{rev}(t_c)$ based on values of $\bar{k}_{irr}(t_c)$, $K$, and $t_c$. It was used to obtained values of $\bar{k}_{rev}(t_c)$ for the examples of reversible homodimerization of rows 9 and 10 in Table 1 of the main text.
Note that the last two terms of the right-hand side of Eq (S17) are independent of diffusion. By introducing an auxiliary constant $\tilde{k}_0$ such that $(\tilde{k}_0)^{-1} = \left(k_{on}^{(h)}\right)^{-1} + t_cK$, the equation for $\bar{k}_{rev}(t_c)$ can be written in the form similar to Eq (S14): $\bar{k}_{rev}(t_c) = \tilde{k}_0\bar{k}_D(t_c)/(\tilde{k}_0 + \bar{k}_D(t_c))$, and in similarity to Eq (15), $\delta_{rev} = \bar{k}_{rev}(t_c)/\bar{k}_D(t_c)$; this formula was used for computing $\delta_{rev}$ (see the worksheet for reversible binding in the collection of supplementary files). For the reversible reactions of rows 9 and 10 in Table 1 of the main text, $t_c$ = 0.00171 s and 0.8636 s, respectively, and for both cases $\delta_{rev} = 0.7$.

## References

M. Dembo, B. Goldstein, A thermodynamic model of binding of flexible bivalent haptens to antibody. *Immunochemistry* **15**, 307-313 (1978).
P. J. Michalski, L. M. Loew, SpringSaLaD: A Spatial, Particle-Based Biochemical Simulation Platform with Excluded Volume. *Biophys J* **110**, 523-529 (2016).
O. N. Yogurtcu, M. E. Johnson, Theory of bi-molecular association dynamics iin 2D for accurate model and experimental parameterization of binding rates. *J Chem Phys* **143**, 084117 (2015).

J. Keizer, Diffusion Effects on Rapid Bimolecular Chemical Reactions. *Chem Rev* **87**, 167-180 (1987).
F. C. Collins, G. E. Kimball, Diffusion-Controlled Reaction Rates. *J Colloid Sci* **4**, 425-437 (1949).
D. C. Torney, H. M. McConnell, Diffusion-limited reaction rate theory for two-dimensional systems. *Proc Soc* A **387**, 147-170 (1983).
A. V. Barzykin, M. Tachiya, Diffusion-influenced reaction kinetics on fractal structures. *J Chem Phys* **99**, 9591-9597 (1993).
B. M. Slepchenko, L. M. Loew, Use of Virtual Cell in Studies of Cellular Dynamics. Int Rev Cell Mol Biol 283, 1-56 (2010).
D. C. Resasco, F. Gao, F. Morgan, I. L. Novak, J. C. Schaff, B. M. Slepchenko, Virtual Cell: computational tools for modeling in cell biology. *WIREs: Syst Biol Med* **4**, 129-140 (2012).
B. M. Slepchenko, J. C. Schaff, L. M. Loew, Spatial Modeling of Cellular Systems with VCell. In *Quantitative Biology. Theory, Computational Methods, and Examples of Models*, edited by B. Munsky, W. S. Hlavacek, and L. S, Tsimring. (MIT Press, Cambridge MA, 2018), Chapter 22, p. 455-468.
J. B. Pedersen, The reactivity dependence of the recombination probability. *J Chem Phys* **72**, 3904-3908 (1980).
M. Tachiya, Theory of diffusion-controlled reaction: formulation of the bulk reaction rate in terms pof the pair probability. *Radiat Phys Chem* 21, 167-175 (1983).
A. Szabo, Theory of Diffusion-Influenced Fluorescence Quenching. *J Phys Chem* **93**, 6929-6939 (1989).
N. Agmon, A. Szabo, Theory of reversible diffusion-influenced reactions. . *J Chem Phys* **92**, 5270-5284 (1990).
